# Novel Loci for Adiponectin Levels and Their Influence on Type 2 Diabetes and Metabolic Traits: A Multi-Ethnic Meta-Analysis of 45,891 Individuals

**DOI:** 10.1371/journal.pgen.1002607

**Published:** 2012-03-29

**Authors:** Zari Dastani, Marie-France Hivert, Nicholas Timpson, John R. B. Perry, Xin Yuan, Robert A. Scott, Peter Henneman, Iris M. Heid, Jorge R. Kizer, Leo-Pekka Lyytikäinen, Christian Fuchsberger, Toshiko Tanaka, Andrew P. Morris, Kerrin Small, Aaron Isaacs, Marian Beekman, Stefan Coassin, Kurt Lohman, Lu Qi, Stavroula Kanoni, James S. Pankow, Hae-Won Uh, Ying Wu, Aurelian Bidulescu, Laura J. Rasmussen-Torvik, Celia M. T. Greenwood, Martin Ladouceur, Jonna Grimsby, Alisa K. Manning, Ching-Ti Liu, Jaspal Kooner, Vincent E. Mooser, Peter Vollenweider, Karen A. Kapur, John Chambers, Nicholas J. Wareham, Claudia Langenberg, Rune Frants, Ko Willems-vanDijk, Ben A. Oostra, Sara M. Willems, Claudia Lamina, Thomas W. Winkler, Bruce M. Psaty, Russell P. Tracy, Jennifer Brody, Ida Chen, Jorma Viikari, Mika Kähönen, Peter P. Pramstaller, David M. Evans, Beate St. Pourcain, Naveed Sattar, Andrew R. Wood, Stefania Bandinelli, Olga D. Carlson, Josephine M. Egan, Stefan Böhringer, Diana van Heemst, Lyudmyla Kedenko, Kati Kristiansson, Marja-Liisa Nuotio, Britt-Marie Loo, Tamara Harris, Melissa Garcia, Alka Kanaya, Margot Haun, Norman Klopp, H.-Erich Wichmann, Panos Deloukas, Efi Katsareli, David J. Couper, Bruce B. Duncan, Margreet Kloppenburg, Linda S. Adair, Judith B. Borja, DIAGRAM+ Consortium, MAGIC Consortium, GLGC Investigators, MuTHER Consortium, James G. Wilson, Solomon Musani, Xiuqing Guo, Toby Johnson, Robert Semple, Tanya M. Teslovich, Matthew A. Allison, Susan Redline, Sarah G. Buxbaum, Karen L. Mohlke, Ingrid Meulenbelt, Christie M. Ballantyne, George V. Dedoussis, Frank B. Hu, Yongmei Liu, Bernhard Paulweber, Timothy D. Spector, P. Eline Slagboom, Luigi Ferrucci, Antti Jula, Markus Perola, Olli Raitakari, Jose C. Florez, Veikko Salomaa, Johan G. Eriksson, Timothy M. Frayling, Andrew A. Hicks, Terho Lehtimäki, George Davey Smith, David S. Siscovick, Florian Kronenberg, Cornelia van Duijn, Ruth J. F. Loos, Dawn M. Waterworth, James B. Meigs, Josee Dupuis, J. Brent Richards

**Affiliations:** 1 Department of Epidemiology, Biostatistics, and Occupational Health, Jewish General Hospital, Lady Davis Institute, McGill University, Montreal, Canada; 2 Department of Medicine, Université de Sherbrooke, Sherbrooke, Canada; 3 General Medicine Division, Massachusetts General Hospital, Boston, Massachusetts, United States of America; 4 MRC CAiTE Centre and School of Social and Community Medicine, University of Bristol, Bristol, United Kingdom; 5 Wellcome Trust Centre for Human Genetics, University of Oxford, Oxford, United Kingdom; 6 Genetics of Complex Traits, Peninsula Medical School, University of Exeter, Exeter, United Kingdom; 7 Genetics, GlaxoSmithKline, King of Prussia, Pennsylvania, United States of America; 8 MRC Epidemiology Unit, Institute of Metabolic Science, Addenbrooke's Hospital, Cambridge, United Kingdom; 9 Department of Human Genetics, Leiden University Medical Center, Leiden, The Netherlands; 10 Department of Epidemiology and Preventive Medicine, Regensburg University Medical Center, Regensburg, Germany; 11 Departments of Medicine and Public Health, Weill Cornell Medical College, New York, New York, United States of America; 12 Department of Clinical Chemistry, University of Tampere and Tampere University Hospital, Tampere, Finland; 13 Center for Statistical Genetics, Department of Biostatistics, University of Michigan, Ann Arbor, Michigan, United States of America; 14 Clinical Research Branch, National Institute on Aging, Baltimore, Maryland, United States of America; 15 Department of Twin Research and Genetic Epidemiology, King's College London, London, United Kingdom; 16 Wellcome Trust Sanger Institute, Wellcome Trust Genome Campus, Hinxton, United Kingdom; 17 Genetic Epidemiology Unit, Department of Epidemiology, Erasmus Medical Center, Rotterdam, The Netherlands; 18 Centre for Medical Systems Biology, Leiden, The Netherlands; 19 Section of Molecular Epidemiology, Leiden University Medical Center and The Netherlands Genomics Initiative, The Netherlands Consortium for Healthy Aging, Leiden, The Netherlands; 20 Division of Genetic Epidemiology, Innsbruck Medical University, Innsbruck, Austria; 21 Wake Forest University School of Medicine, Winston-Salem, North Carolina, United States of America; 22 Harvard School of Public Health, Boston, Massachusetts, United States of America; 23 Division of Epidemiology and Community Health, University of Minnesota, Minneapolis, Minnesota, United States of America; 24 Medical Statistics and Bioinformatics, Leiden University Medical Center, Leiden, The Netherlands; 25 Department of Genetics, University of North Carolina, Chapel Hill, North Carolina, United States of America; 26 Cardiovascular Research Institute, Morehouse School of Medicine, Atlanta, Georgia, United States of America; 27 Department of Preventive Medicine, Feinberg School of Medicine, Northwestern University, Chicago, Illinois, United States of America; 28 Lady Davis Institute for Medical Research, Department of Oncology, McGill University, Montreal, Canada; 29 Department of Human Genetics McGill University, Montreal, Canada; 30 Department of Medicine, Harvard Medical School, Boston, Massachusetts, United States of America; 31 Department of Biostatistics, Boston University School of Public Health, Boston, Massachusetts, United States of America; 32 Cardiology, Ealing Hospital National Health Service (NHS) Trust, London, United Kingdom; 33 Department of Internal Medicine, University of Lausanne, Lausanne, Switzerland; 34 Department of Medical Genetics, University of Lausanne, Lausanne, Switzerland; 35 Epidemiology and Biostatistics, Imperial College London, London, United Kingdom; 36 Deptartment of Clinical Genetics and Department of Epidemiology, Erasmus Medical Center, Rotterdam, The Netherlands; 37 Cardiovascular Health Research Unit, Departments of Medicine and Epidemiology, University of Washington, Seattle, Washington, United States of America; 38 Group Health Research Institute, Group Health Cooperative, Seattle, Washington, United States of America; 39 Departments of Pathology and Biochemistry, University of Vermont, Burlington, Vermont, United States of America; 40 Cardiovascular Health Research Unit, University of Washington, Seattle, Washington, United States of America; 41 Medical Genetics Research Institute, Cedars Sinai Medical Center, Los Angeles, California, United States of America; 42 Department of Medicine, University of Turku and Turku University Hospital, Turku, Finland; 43 Department of Clinical Physiology, University of Tampere and Tampere University Hospital, Tampere, Finland; 44 Center for Biomedicine, European Academy Bozen/Bolzano (EURAC) (Affiliated Institute of the University of Lübeck, Lübeck, Germany), Bolzano, Italy; 45 Department of Neurology, General Central Hospital, Bolzano, Italy; 46 Department of Neurology, University of Lübeck, Lübeck, Germany; 47 School of Social and community medicine, University of Bristol, Bristol, United Kingdom; 48 British Heart Foundation Glasgow Cardiovascular Research Centre, University of Glasgow, Glasgow, United Kingdom; 49 Geriatric Unit, Azienda Sanitaria Firenze (ASF), Florence, Italy; 50 Laboratory of Clinical Investigation, National Institute of Aging, Baltimore, Maryland, United States of America; 51 Gerontology and Geriatrics, Leiden University Medical Center, Leiden, The Netherlands; 52 First Department of Internal Medicine, St. Johann Spital, Paracelsus Private Medical University Salzburg, Salzburg, Austria; 53 Public Health Genomics Unit, Department of Chronic Disease Prevention, National Institute for Health and Welfare, and Institute for Molecular Medicine Finland FIMM, University of Helsinki, Helsinki, Finland; 54 Population Studies Unit, Department of Chronic Disease Prevention, National Institute for Health and Welfare, Turku, Finland; 55 Intramural Research Program, Laboratory of Epidemiology, Demography, and Biometry, National Institute on Aging, National Institutes of Health, Bethesda, Maryland, United States of America; 56 Division of General Internal Medicine, Women's Health Clinical Research Center, University of California San Francisco, San Francisco, California, United States of America; 57 Institute of Epidemiology, Helmholtz Zentrum München, German Research Center for Environmental Health, Munich, Germany; 58 Institute of Medical Informatics, Biometry and Epidemiology, Ludwig-Maximilians-Universität, Munich, Germany; 59 Klinikum Großhadern, Munich, Germany; 60 Harokopio University, Athens, Greece; 61 Collaborative Studies Coordinating Center, Department of Biostatistics, University of North Carolina at Chapel Hill, Chapel Hill, North Carolina, United States of America; 62 School of Medicine, Federal University of Rio Grande do Sul, Porto Alegre, Brazil; 63 Department of Epidemiology, University of North Carolina at Chapel Hill, Chapel Hill, North Carolina, United States of America; 64 Department of Rheumatology and Department of Clinical Epidemiology, Leiden, The Netherlands; 65 Department of Nutrition, University of North Carolina, Chapel Hill, North Carolina, United States of America; 66 Office of Population Studies Foundation, University of San Carlos, Cebu City, Philippines; 67 Department of Physiology and Biophysics, University of Mississippi Medical Center, Jackson, Mississippi, United States of America; 68 Department of Medicine, University of Mississippi Medical Center, Jackson, Mississippi, United States of America; 69 Medical Genetics Institute, Cedars-Sinai Medical Center, Los Angeles, California, United States of America; 70 University Institute of Social and Preventative Medicine, Centre Hospitalier Universitaire Vaudois (CHUV) and University of Lausanne, Lausanne, Switzerland; 71 Swiss Institute of Bioinformatics, Lausanne, Switzerland; 72 Metabolic Research Laboratories, Institute of Metabolic Science, University of Cambridge, Addenbrooke's Hospital, Cambridge, United Kingdom; 73 Department of Family and Preventive Medicine, University of California San Diego, La Jolla, California, United States of America; 74 Brigham and Women's Hospital, Boston, Massachusetts, United States of America; 75 Jackson Heart Study Coordinating Center, Jackson State University, Jackson, Mississippi, United States of America; 76 Baylor College of Medicine and Methodist DeBakey Heart and Vascular Center, Houston, Texas, United States of America; 77 Molecular Epidemiology, Leiden University Medical Center, Leiden, The Netherlands; 78 Research Centre of Applied and Preventive Cardiovascular Medicine, University of Turku and the Department of Clinical Physiology, Turku University Hospital, Turku, Finland; 79 Program in Medical and Population Genetics, Broad Institute, Cambridge, Massachusetts, United States of America; 80 Center for Human Genetic Research, Massachusetts General Hospital, Boston, Massachusetts, United States of America; 81 Diabetes Research Center, Diabetes Unit, Massachusetts General Hospital, Boston, Massachusetts, United States of America; 82 Chronic Disease Epidemiology and Prevention Unit, Department of Chronic Disease Prevention, National Institute for Health and Welfare, Helsinki, Finland; 83 Diabetes Prevention Unit, Department of Chronic Disease Prevention, National Institute for Health and Welfare, Helsinki, Finland; 84 Unit of General Practice, Helsinki University Central Hospital, Helsinki, Finland; 85 Folkhalsan Research Centre, Helsinki, Finland; 86 Vaasa Central Hospital, Vaasa, Finland; 87 Department of General Practice and Primary Health Care, University of Helsinki, Helsinki, Finland; 88 University of Washington, Seattle, Washington, United States of America; 89 National Heart, Lung, and Blood Institute's Framingham Heart Study, Framingham, Massachusetts, United States of America; 90 Departments of Medicine, Human Genetics, Epidemiology, and Biostatistics, Lady Davis Institute, Jewish General Hospital, McGill University, Montreal, Canada; The University of Queensland, Australia

## Abstract

Circulating levels of adiponectin, a hormone produced predominantly by adipocytes, are highly heritable and are inversely associated with type 2 diabetes mellitus (T2D) and other metabolic traits. We conducted a meta-analysis of genome-wide association studies in 39,883 individuals of European ancestry to identify genes associated with metabolic disease. We identified 8 novel loci associated with adiponectin levels and confirmed 2 previously reported loci (*P* = 4.5×10^−8^–1.2×10^−43^). Using a novel method to combine data across ethnicities (N = 4,232 African Americans, N = 1,776 Asians, and N = 29,347 Europeans), we identified two additional novel loci. Expression analyses of 436 human adipocyte samples revealed that mRNA levels of 18 genes at candidate regions were associated with adiponectin concentrations after accounting for multiple testing (*p*<3×10^−4^). We next developed a multi-SNP genotypic risk score to test the association of adiponectin decreasing risk alleles on metabolic traits and diseases using consortia-level meta-analytic data. This risk score was associated with increased risk of T2D (*p* = 4.3×10^−3^, n = 22,044), increased triglycerides (*p* = 2.6×10^−14^, n = 93,440), increased waist-to-hip ratio (*p* = 1.8×10^−5^, n = 77,167), increased glucose two hours post oral glucose tolerance testing (*p* = 4.4×10^−3^, n = 15,234), increased fasting insulin (*p* = 0.015, n = 48,238), but with lower in HDL-cholesterol concentrations (*p* = 4.5×10^−13^, n = 96,748) and decreased BMI (*p* = 1.4×10^−4^, n = 121,335). These findings identify novel genetic determinants of adiponectin levels, which, taken together, influence risk of T2D and markers of insulin resistance.

## Author Summary

Serum adiponectin levels are highly heritable and are inversely correlated with the risk of type 2 diabetes (T2D), coronary artery disease, stroke, and several metabolic traits. To identify common genetic variants associated with adiponectin levels and risk of T2D and metabolic traits, we conducted a meta-analysis of genome-wide association studies of 45,891 multi-ethnic individuals. In addition to confirming that variants at the *ADIPOQ* and *CDH13* loci influence adiponectin levels, our analyses revealed that 10 new loci also affecting circulating adiponectin levels. We demonstrated that expression levels of several genes in these candidate regions are associated with serum adiponectin levels. Using a powerful novel method to assess the contribution of the identified variants with other traits using summary-level results from large-scale GWAS consortia, we provide evidence that the risk alleles for adiponectin are associated with deleterious changes in T2D risk and metabolic syndrome traits (triglycerides, HDL, post-prandial glucose, insulin, and waist-to-hip ratio), demonstrating that the identified loci, taken together, impact upon metabolic disease.

## Introduction

Adiponectin is a highly abundant adipocyte-derived plasma protein whose levels correlate inversely with a range of important clinical parameters including blood glucose, indices of insulin resistance, proatherogenic dyslipidemia, and risk of type 2 diabetes (T2D), stroke and coronary artery disease [1], [2], [3], [4]. Collectively these conditions account for most of the burgeoning pandemic of obesity-related morbidity and mortality that poses a severe and global healthcare challenge [5]. Murine studies suggest that adiponectin plays a mediating role in at least some of these obesity-related complications, and although less clearly established in humans, this suggests that understanding the pathophysiology of adiponectin may uncover novel therapeutic targets in major, highly prevalent human disease.[6], [7].

Twins and family studies have revealed moderate to high estimates of heritability (30–70%) for plasma adiponectin levels [8], [9], [10], [11]. However, until recently, few genes associated with adiponectin levels have been identified. Candidate and genome-wide association studies (GWAS) have shown pronounced associations between common polymorphisms in the adiponectin gene (*ADIPOQ*) and adiponectin levels [12], [13], [14], [15]. A recent meta-analysis of three GWAS for adiponectin levels identified variants in a novel candidate gene, *ARL15*, that were associated with adiponectin levels, coronary heart disease (CHD), T2D and other metabolic traits [16]. Furthermore, *CDH13* and *KNG1* genes were found to be associated with adiponectin levels in two studies involving East Asian populations [17], [18]. Although part of the variance explained by the *ADIPOQ* locus, most of the heritability of adiponectin levels remains unaccounted for. Therefore, we sought to identify novel common variants influencing adiponectin levels and test their association with risk of T2D and related metabolic traits within the framework of a large multi-ethnic consortium of GWAS.

We combined genome-wide association results of 35,355 individuals from three different ethnicities (white Europeans (n = 29,347), African American s(n = 4,232) and East Asians (n = 1,776)), applying a novel meta-analytic method to allow for heterogeneity in allelic effects between populations of different ethnic backgrounds. We next examined whether identified genome-wide significant single nucleotide polymorphisms (SNPs) also associated with expression of their nearest gene in human adipocytes, the main source of adiponectin. Since adiponectin has been associated with T2D, insulin resistance and metabolic traits we next investigated whether a multi-SNP genotypic risk, comprising genome-wide significant SNPs for adiponectin levels, also influenced risk of T2D and related traits measured in the DIAbetes Genetics Replication and Meta-analysis (DIAGRAM+) [19], Meta-Analysis of Glucose and Insulin Related Traits Consortium (MAGIC) [20], Genetic Investigation of ANthropometric measures Traits (GIANT) [21] , Global Lipids Genetic Consortium (GLGC) [22], and Body Fat GWAS consortia [23].

## Results

### Results of Meta-Analysis of GWAS

The meta-analysis was divided into four phases 1) Discovery phase, which involved cohorts providing GWAS results, 2) In-silico replication phase which included additional GWAS cohorts joining our meta-analysis after the completion of the discovery phase, 3) De-novo genotyping in cohorts without GWAS genotyping and 4) Multi-Ethnic meta-analysis applying a novel method for complex trait mapping using different ethnicities.

#### Discovery phase in individuals of white European origin

**Figure 1:**
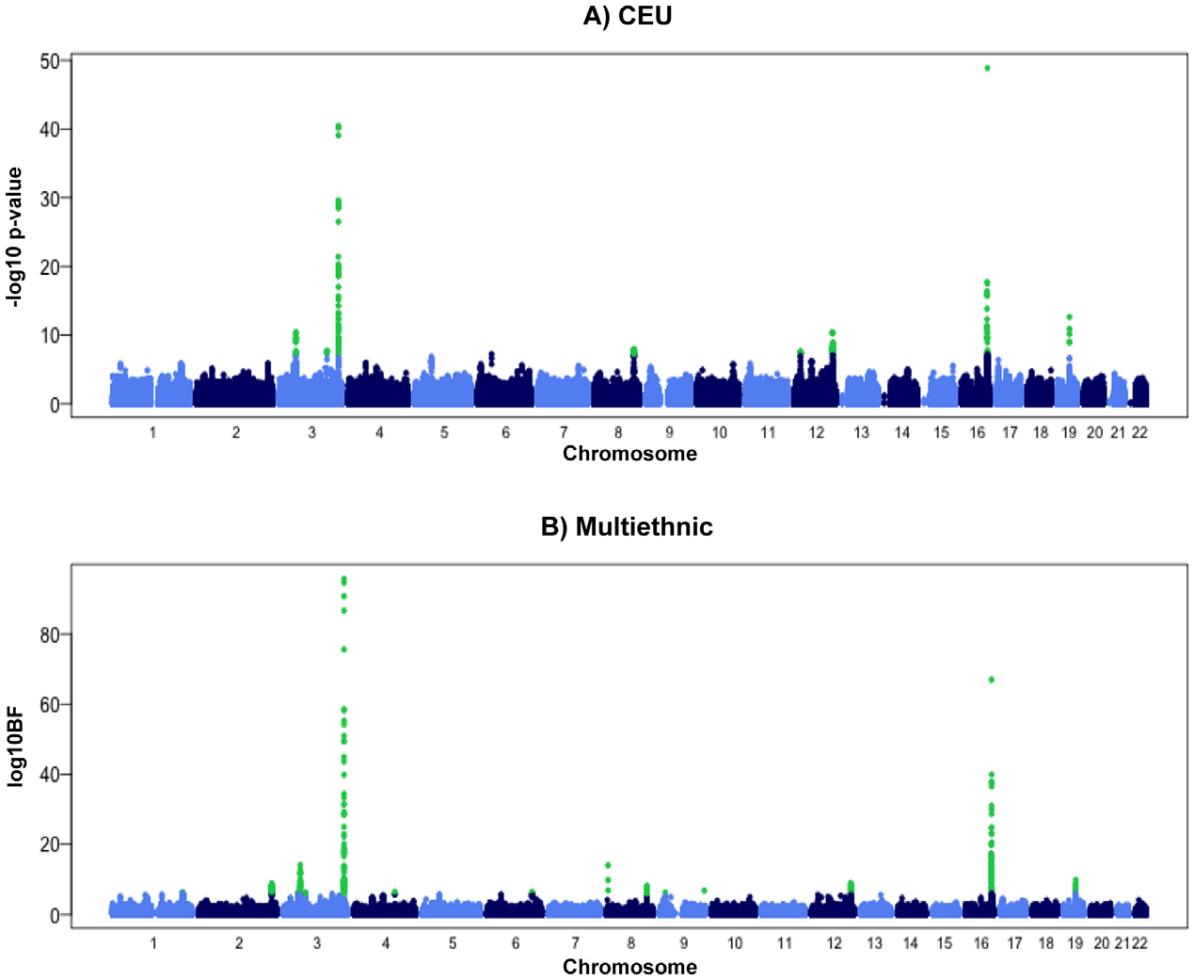
Manhattan plots for meta-analyses in the discovery phase. A) Combined sex analysis in European populations, B) Meta-Analysis of Multiple Ethnicities. The Manhattan plots show −Log_10_ (*p*-value) measures for association between single nucleotide polymorphisms (SNPs) and chromosomal position. The SNPs that achieved genome-wide significance are highlighted in green.

**Figure 2:**
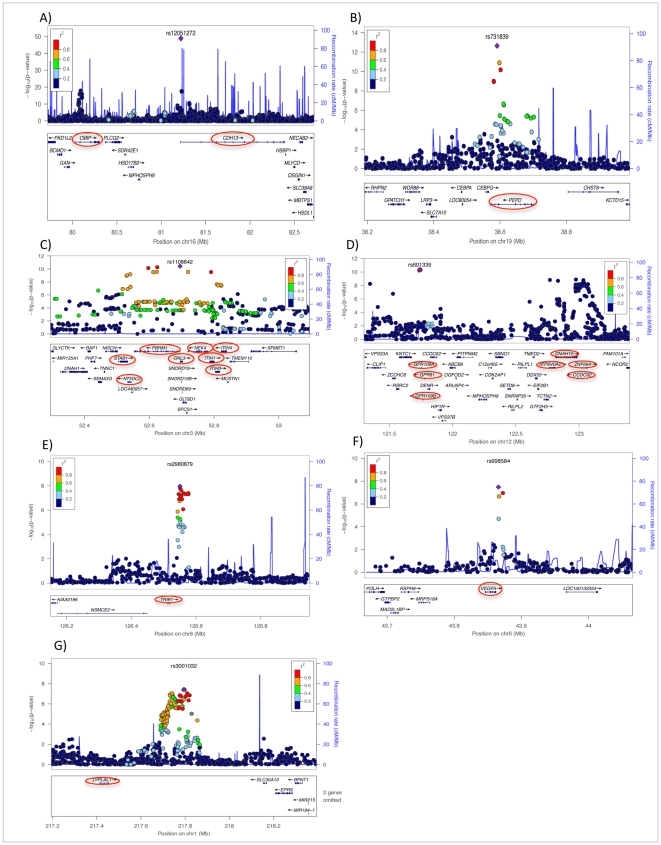
Regional plots of eight newly discovered genome-wide significant chromosomal regions associated with adiponectin concentrations in European populations. A) chromosome 16q23.2, B) chromosome 19 q13.11 C) Chromosome 3p21.1, D) two loci on chromosome 12q24.31, E) chromosome 8q24.13, F) chromosome 6p21.1, and G) chromosome 1q41. In each panel, purple diamonds indicate the top SNPs, which have the strongest evidence of association. Each circle shows a SNP with a color scale relating the r^2^ value for that SNP and the top SNP from HapMap CEU. Blue lines indicate estimated recombination rates from HapMap. The bottom panels illustrate the relative position of genes near each locus. Candidate genes are indicated by red ovals.

The meta-analysis of sex-combined data from 16 GWAS (n = 29,347) of individuals of white European descent identified ten loci associated with adiponectin levels at *p*≤5.0×10^−8^ (Table 1 and Figure 1A and Figure S1, Table S2). These results include the previously described associations with adiponectin at *ADIPOQ* (rs6810075[T]; ß = 0.06, *p*-value = 3.60×10^−41^), *KNG1* (rs2062632[T]; ß = 0.05, *p*-value = 2.52×10^−19^) on 3q27.3, and *CDH13* (rs12922394[T; ß = −0.1, *p* = 3.16×10^−18^) on 16q23.3 (Table 1). Furthermore, we identified variants that showed genome-wide significant association in eight novel independent loci including rs9853056 (within the *STAB1 gene*, rs4282054 (within the *NT5DC2 gene*), rs13083798 (within the *PBRM1* gene), rs1108842 (within the *GNL3* gene), rs11235 (within the *NEK4* gene), rs2710323 (within the *ITIH1* gene), rs3617 (within the *ITIH3* gene), and rs2535627 (within 200 Kb of *ITIH4* gene) at 3p21.1; rs1597466 (within 1 Mb of *TSC22D2* gene) at 3q25.1; rs2980879 (within 1 Mb of *TRIB1* gene) at 8q24.13; rs7955516 (within 1.3 Mb *PDE3A* gene) at 12p12.2; rs601339 (within the *GPR109A* gene) at 12q24.31; rs6488898 (within the *ATP6V0A2* gene), rs7133378 (within *the DNAH10* gene), rs7305864 (within the *CCDC92* gene), and rs7978610 (within *the ZNF664* gene at 12q24.31, which is 1.3 Mb away from *GPR109A*); rs2925979 (within the *CMIP* at 16q23.2 gene); and rs731839 (within *the PEPD* gene) at 19q13.11. (Figure 2A–2E, Table 1).

**Table 1:** Lead SNP per Locus for Genome-Wide Significant SNPs Arising from the Sex-Combined Meta-Analysis in European Populations.

*Nearest* ** *Gene*	*Lead SNP* ‡	*Region*	*Chr/position†*	*EA/NEA* ¶	*EAF* ¶¶	*Beta* §	*SE*	*P*	*I2*	*n*	*Beta* §	*SE*	*P*	*I2*	*n*
						Discovery Phase Results					Joint Analysis Phase*				
*LYPLAL1*	*rs3001032*	*1q41*	*1/217794402*	*T/C*	*0.7*	*−0.02*	*0.005*	*1.98E-06*	*0*	*29,321*	*−0.02*	*0.004*	*3.60E-08*	*0*	*35,930*
*GNL3*	*rs1108842*	*3p21.1*	*3/52695120*	*C/A*	*0.50*	*0.03*	*0.004*	*3.66E-11*	*0.33*	*29,338*	*0.03*	*0.004*	*1.39E-13*	*0.2*	*35,962*
TSC22D2	rs1597466	3q25.1	3/151538251	T/G	0.1	−0.04	0.008	1.88E-08	0	29,319	−0.03	0.007	1.62E-06	0.1	35,794
*ADIPOQ*	*rs6810075*	*3q27.3*	*3/188031259*	*T/C*	*0.6*	*0.06*	*0.005*	*3.60E-41*	*0*	*29,140*	*0.06*	*0.004*	*1.19E-43*	*0*	*35,749*
*VEGFA*	*rs998584*	*6q21.1*	*6/43865874*	*C/A*	*0.5*	*0.03*	*0.005*	*5.84E-08*	*0.3*	*28,167*	*0.03*	*0.005*	*3.25E-08*	*0.2*	*34,108*
*TRIB1*	*rs2980879*	*8q24.13*	*8/126550657*	*T/A*	*0.7*	*0.03*	*0.005*	*1.08E-08*	*0*	*24,084*	*0.03*	*0.005*	*7.13E-09*	*0*	*30,708*
*PDE3A*	*rs7955516*	*12q12.2*	*12/20389303*	*C/A*	*0.4*	*0.03*	*0.005*	*2.43E-08*	*0.1*	*29,178*	*0.02*	*0.004*	*4.45E-08*	*0*	*38,276*
*GPR109A*	*rs601339*	*12q24.31*	*12/121740696*	*G/A*	*0.2*	*0.04*	*0.006*	*3.87E-11*	*0*	*29,325*	*0.03*	*0.005*	*7.81E-10*	*0.3*	*35,947*
DNAH10	rs7133378	12q24.31	12/122975455	G/A	0.7	−0.03	0.005	1.29E-09	0	29,223	−0.02	0.004	6.21E-07	0.5	35,697
*CMIP*	*rs2925979*	*16q23.2*	*16/80092291*	*T/C*	*0.3*	*−0.04*	*0.005*	*1.87E-18*	*0*	*29,347*	*−0.04*	*0.005*	*1.21E-20*	*0*	*35,970*
*CDH13*	*rs12922394*	*16q23.3*	*16/81229828*	*T/C*	*0.1*	*−0.10*	*0.011*	*3.16E-18*	*0.3*	*24,466*	*−0.08*	*0.010*	*1.99E-15*	*0.4*	*31,089*
*PEPD*	*rs731839*	*19q13.11*	*19/38590905*	*G/A*	*0.35*	*−0.04*	*0.005*	*2.20E-13*	*0.03*	*29,166*	*−0.03*	*0.004*	*7.97E-12*	*0.4*	*35,771*

*All SNPs achieving genome-wide significance in the joint analysis phase are marked in italics.*

*: Joint analysis indicates results from the meta-analysis of discovery and follow-up *in-silico* and *de-novo* phases.

**: When possible, plausible biological candidate genes have been listed; otherwise, the closest gene is designated.

**‡:** Lead SNP is the SNP with the lowest *p*-value for each locus.

**§:** Betas are estimated from models using the natural log transformed adiponectin.

**¶:** EA: Effect allele, NEA: Non-effect allele.

**¶¶:** EAF: Effect allele frequency.

In our analysis a common variant (rs601339, MAF = 0.18, allele G) downstream of the GPR109A gene (the putative niacin receptor) was associated with adiponectin (ß = 0.04, p = 7.94×10^−10^) and HDL-C (ß = 0.03, p = 5.59×10^−7^) in the global lipid analysis. In a coincident candidate gene analysis 11 SNPs were typed in GPR109A/B in CoLaus and LOLIPOP cohorts, containing individuals of European descent. A single nominally significant coding SNP R311C (rs7314976, MAF = 0.14) within the GPR109A gene was taken forward for replication and found to be consistently associated with adiponectin in the three cohorts (CoLaus, Fenland and MRC Ely study, n = 8285, p = 4.6×10^−8^) and HDL-cholesterol (HDL-C) in four cohorts (CoLaus, Fenland, Ely study and Lolipop, n = 18425, p = 2.9×10^−8^) (Figure S2A, S2B). However R311C and rs601339 were not in linkage disequilibirium with each other (r2 = 0.04). Therefore the two variants represent two independent signals from the same locus but with similar effects on HDL-cholesterol and adiponectin.

#### In silico follow-up phase

In the *in-silico* follow-up phase 468 SNPs demonstrating genome-wide significant (*p*<5×10^−8^ ) or suggestive (*p*<5×10^−6^) association with adiponectin in the discovery phase were tested for association in additional European cohorts. (Table S3). These SNPs were tested in seven additional GWAS datasets (n = 6,623 from NHS, HPFS, HABC, ERF2, LLS, GARP and ARIC studies) and the combined meta-analysis of the discovery and follow-up *in-silico* GWAS datasets detected additional loci on chromosomes 1q41 near the *LYPLAL1* gene (rs3001032, *p* = 3.6×10^−8^) and chromosome 6p21.1 near the *VEGFA* gene (rs998584, *p* = 5.8×10^−12^) that reached genome-wide significance. While we confirmed seven loci that had reached genome-wide significance at the discovery stage (Table 1, Figure 2F and 2G, Table S2), two identified loci (3q25.1 and 12q24.31) did not remain genome-wide significant in the joint analysis of discovery and follow-up results.

#### De novo follow-up phase

Next, in the *de-novo* genotyping follow-up phase, we genotyped 10 SNPs with suggestive evidence of association (5×10^−8^<*p*<5×10^−6^) from the meta-analysis of the discovery and *in-silico* follow-up phases in an additional 3,913 individuals. Meta-analyzing the discovery and 2 follow-up stages identified a SNP in *ARL15* (rs6450176 [G]; ß = 0.026, *p* = 5.8×10^−8^), which was initially described in a previous GWAS for adiponectin levels (Table S3) [16].

#### Multi-ethnic meta-analysis

To identify loci influencing adiponectin levels in non-European individuals we performed an additional analysis in 4,232 individuals from an African American population and 1,776 individuals from an East Asian population. In the African American populations, only associations at the *ADIPOQ* locus reached genome-wide significance, while in the East Asian population there was evidence of association at the *ADIPOQ* and *CDH13* loci (Table S4). Subsequently, we performed a meta-analysis that combined all available GWAS including those of white European origin, African American and East Asian ancestry using novel method MANTRA [24]. This analysis identified two additional loci in or near *IRS1 gene* on 2q36.3 and at 6q24.1 within a gene desert. (Table 2, Figure 1B).

**Table 2:** Genome-Wide Significant SNPs from the Sex-Combined Multi-Ethnic Meta-Analysis.

Nearby* Gene	Lead SNP‡	Gene region	chr/position†	EA/NEA¶	EAF¶¶ (CEU/EA/AA)	Multi-Ethnic Fixed Effects Meta-analysis				Multi-Ethnic Random Effects Meta-analysis		MANTRA		N
						Beta (SE)	pvalue	Q-Value	I2	Beta (SE)	pvalue	BF§	phet††	
*LYPLAL1*	rs2791553	1q41	1/217742665	G/A	0.6/0.46/0.54	−0.02(0.004)	4.91E-07	25.18	0	−0.02(0.004)	4.91E-07	6.3	0.06	37,665
***IRS1***	**rs925735**	**2q36.3**	**2/226887874**	**G/C**	**0.64/0.89/0.74**	**−0.02(0.004)**	**1.88E-08**	**22.15**	**0.01**	**−0.02(0.004)**	**2.12E-08**	**8.1**	**0.06**	**37,638**
*GNL3*	rs2590838	3p21.1	3/52597126	G/A	0.5 1/0.34/0.54	−0.03(0.004)	4.08E-15	28.85	0.06	−0.03(0.004)	1.88E-13	14.1	0.05	37,680
*ADIPOQ*	rs6810075	3q27.3	3/188031259	T/C	0.93/1/0.86	0.06(0.004)	1.10E-43	27.44	0.02	0.06(0.004)	2.41E-42	43.6	0.16	31,533
***-***	**rs592423**	**6q24.1**	**6/139882386**	**C/A**	**0.54/0.36/0.41**	**0.02(0.004)**	**3.59E-07**	**15.46**	**0**	**0.02(0.004)**	**3.59E-07**	**6.5**	**0.03**	**37,430**
*TRIB1*	rs2980879	8q24.13	8/126550657	T/A	0.69/0.77/0.67	0.03(0.004)	9.91E-10	21.08	0	0.03(0.004)	9.91E-10	8.2	0.04	32,426
*GPR109A*	rs601339	12q24.31	12/121740696	G/A	0.19/0.39/0.31	0.03(0.005)	3.77E-09	36.11	0.25	0.03(0.006)	4.31E-06	8.3	0.09	37666
*CMIP*	rs2925979	16q23.2	16/80092291	T/C	0.3 0/0.43/0.31	−0.04(0.004)	3.12E-21	23.12	0	−0.04(0.004)	3.12E-21	19.8	0.31	37,687
*CDH13*	rs12051272	16q23.3	16/81220789	T/G	0.03/0.33/0.03	−0.26(0.017)	4.74E-51	39.17	0.62	−0.26(0.032)	1.10E-14	66.0	1.00	24,216
*PEPD*	rs4805885	19q13.11	19/38597963	T/C	0.39/0.64/0.41	−0.03(0.004)	1.65E-11	34.94	0.23	−0.03(0.005)	2.05E-08	9.9	0.05	37,479

The novel loci identified using Multi-Ethnic Meta-analysis (that were not identified in the European only analysis) are listed in **bold**.

*: When possible, plausible biological candidate genes have been listed; otherwise, the closest gene is designated.

**‡:** Lead SNP is the SNP with the lowest *p*-value for each locus.

**†:** Positions are relative to Human Genome NCBI Build 36.

**§:** log_10_ Bayes factor (BF) from the MANTRA analysis. A log_10_ BF of 6 and higher was considered as a conservative threshold for genome-wide significance.

**††:** The posterior probability of heterogeneity between studies.

**¶:** EA: effect allele, NEA: non-effect allele.

**¶¶:** EAF: Frequency of effect allele in CEU, East Asian, and AA, populations respectively.

#### Secondary GWAS analyses

We next performed meta-analysis of the GWAS data in women (n = 16,685) and men (n = 12,662) separately (Figure S2A, S2B, Tables S5 and S6). Although no novel loci reached genome-wide significance in women or men separately, three loci (chromosome 3p, 8 and 12) associated with adiponectin levels in the sex-combined analysis showed evidence of association (p value<5×10−8) in women (Figure S3). Since different assays were used to measure adiponectin levels, we next tested whether stratification by assay rendered similar results and found the results were highly concordant with the combined analysis. GWAS for high molecular weight adiponectin in the CHS study (n = 2,718) identified 2 SNPs in *ADIPOQ* (rs17300539, *p* = 3.0×10^−16^) and *CMIP* (rs2927307, *p* = 2.7×10^−8^). These two genes are located within the loci identified in our discovery meta-analysis of adiponectin levels.

### Gene Expression Studies

Through gene expression studies we sought to address two questions: First, are any of the SNPs that were genome-wide significant for adiponectin levels associated with expression of their nearest transcripts (*cis*-eQTLs) and second, whether mRNA levels of loci identified through the GWAS for adiponectin levels are associated with circulating adiponectin levels. To address the first question, we examined whether SNPs within 1 Mb of the SNPs achieving genome-wide significance in the discovery stage were associated with the expression levels of nearby genes in human adipocytes from 776 participants of the MuTHER Consortium [25]. We identified 74 SNPs in three eQTLs to be associated with the expression of five genes in adipocytes, using an array-wide level of statistical significance for eQTLs (P<5.1×10^−5^. See Materials and Methods for details). These genes included: *NT5DC2* on chromosome 3; *CCDC92*, *GPR109A*, and *ZNF664* on chromosome12; and *PEPD* on chromosome 19 (Table 3). The *cis*-eQTL SNPs often are proxies for the lead SNPs from the GWAS, however, this relationship may also be influenced through mechanisms that are independent from gene expression, such as gene function.

**Table 3:** The Association of Lead Genome-Wide Significant SNPs for Adiponectin with mRNA Levels of Their Nearest Gene.

Gene	Lead SNP-Cis-eQTL‡	Chr	Transcript Start Site	Transcript End Site	EA¶	EAF¶¶	Beta (SE)§	P-Exp*	P-GWAS**	lead SNP-GWAS‡‡	r^2^ $
*NT5DC2*	rs13081028	3	52533424	52544133	G	0.444	0.14(0.02)	1.32E-19	1.05E-09	rs1108842	0.84
*GPR109A*	rs2454722*	12	121778105	121781082	G	0.166	−0.15(0.03)	1.71E-09	3.87E-11	rs601339	1
*CCDC92*	rs10773049	12	122986907	123023116	T	0.611	0.15(0.02)	8.09E-22	2.67E-08	rs7133378	0.02
*ZNF664*	rs825453	12	123074711	123065922	T	0.615	−0.04(0.01)	4.51E-05	4.03E-08	rs7978610	0.03
PEPD	rs8182584	19	38569694	38704639	T	0.364	−0.13(0.02)	9.96E-10	6.64E-11	rs731839	1

**‡:** Lead SNP is the SNP with the lowest *p*-value for each gene in gene expression data.

**‡‡:** Lead SNP is the SNP with the lowest *p*-value for each locus in meta-analysis from discovery phase.

**¶:** EA: Effect allele.

**¶¶:** EAF: Frequency of effect allele.

**§:** Betas are estimated expression levels of the genes.

*: P value for lead SNP is the SNP in gene expression data.

**: P value for lead SNP in meta-analysis from discovery phase.

$ r^2^ LD between lead SNP from expression and lead SNP from meta-analysis.

We next identified that mRNA levels of 18 genes arising from six candidate loci were correlated with circulating adiponectin levels (Table 4). Since circulating adiponectin levels may be associated with a surplus of adipocyte transcripts we next tested for enrichment of signal from the candidate loci. There were 133 transcripts in the identified candidate regions, of which 8.2% (11/133) were associated with adiponectin levels at an array-wide level of significance (*p*<2×10^−6^), while 7.5% of the 24k probes on the entire array exceeded the same p-value threshold, indicating there was therefore no additional enrichment of signal at these candidate loci.

**Table 4:** The Association of mRNA Levels from Genes in Candidate Loci in Human Adipocytes with Circulating Adiponectin Levels.

Gene	*Gene region*	GeneStart	GeneEnd	Beta§	Pvalue
*GLYCTK*	3p21.1	52296875	52304311	0.060	1.77E-20
*SEMA3G*	3p21.1	52442307	52454083	−0.018	9.28E-06
*STAB1*	3p21.1	52504395	52533551	−0.039	2.26E-14
*PBRM1*	3p21.1	52554407	52688779	0.007	2.49E-04
*SFMBT1*	3p21.1	52913666	53055110	0.010	2.53E-08
*DNAJB11*	3q27.3	187771160	187786283	−0.014	3.31E-07
*EIF4A2*	3q27.3	187984054	187990379	0.021	1.53E-08
*ADIPOQ*	3q27.3	188043156	188058944	0.054	1.03E-13
*MAD2L1BP*	6q21.1	43711554	43716666	0.009	4.09E-04
*VEGFA*	6q21.1	43845923	43862199	0.012	2.15E-09
*ZCCHC8*	12q24.31*	121523387	121551471	0.011	2.60E-04
*GPR109B*	12q24.31	121765255	121767392	0.010	3.74E-06
*GPR109A*	12q24.31	121778105	121781082	0.026	1.80E-11
*PITPNM2*	12q24.31*	122033979	122160928	−0.010	5.09E-06
*U1SNRNPBP*	12q24.31	122508604	122516894	0.011	1.72E-04
*ATP6V0A2*	12q24.31	122762817	122812252	−0.008	2.86E-04
*ZNF664*	12q24.31	123023622	123065922	0.010	8.28E-06
*SLC7A10*	19q13.11	38391409	38408596	0.072	1.66E-14

**§:** Betas are estimated from log transformed and quantile-quantile normalized values.

*: These two loci are independent loci.

### T2D and Metabolic Traits

Using data from several large-scale GWAS consortia, some of the significantly associated variants identified here demonstrated associations with T2D and its related traits (Table S7A, S7B, S7C, and S7D). Several individual SNPs showed evidence for association with T2D and various metabolic traits after accounting for the number of statistically independent SNPs (p-value threshold of 5×10^−4^) among the SNPs that were genome-wide significant for adiponectin. These include associations with HDL-C (n = 104 SNPs), triglycerides (TG) (n = 65 SNPs), total cholesterol (TC, n = 12 SNPs), LDL-cholesterol (LDL–C, n = 11 SNPs), and waist-hip ratio (WHR) (n = 65 SNPs) [26]. (However, we note that since sample sizes are different across different consortia power to identify associations is not consistent.) Among these, coding and intronic variants in *STAB1* and *NT5DC2* genes were associated with WHR and HDL-C, while the variants 1 Mb near *TRIB1* were associated with all lipid traits. The coding and intronic variants ariants in the locus on chromosome 12 harboring *ZNF664*, *CCDC92*, and *DNAH10* showed evidence of association with WHR, HDL-C, and TG. Finally, variants in the *PEPD* gene were associated with TG.

We next calculated a multi-SNP genotypic risk score based genome-wide significant SNPs from the discovery phase. This multi-SNP genotypic risk score explained 5% of the variance of natural log-transformed adiponectin levels. We then tested the association of this risk score with risk of T2D and metabolic related traits. The multi-SNP genotypic risk score was associated with increased risk for T2D (ß = 0.3, *p* = 4.3×10^−3^), where ß is the average additive effect of adiponectin-decreasing risk alleles on the log odds ratio of T2D), increased TG (ß = 0.25, *p* = 2.6×10^−14^), increased WHR adjusted for BMI (ß = 0.18, *p* = 1.8×10^−5^), increased post-prandial glucose (ß = 0.25, *p* = 0.01), increased fasting insulin (ß = 0.05, *p* = 0.01), homeostatic model assessment- insulin resistance (HOMA-IR) (ß = 0.04, *p* = 0.047), and with lower HDL-C concentrations (ß = −0.24, *p* = 4.5×10^−13^) and decreased BMI (ß = −0.16, p = 1.4×10^−4^). (Table 5).

**Table 5:** Results of Association of Multi-SNP Genotypic Risk Score with Diabetes and Related Traits.

Trait	N	Effect§ (95% CI)	P	Consortium
**T2D** **	**22,044**	**0.301 (0.09, 0.51)**	**4.3E-03**	**DIAGRAM+**
**BMI (SD units)**	**121,335**	**−0.162 (−0.25, −0.08)**	**1.4E-04**	**GIANT**
**WHR** *	**77,167**	**0.177 (0.1, 0.26)**	**1.8E-05**	**GIANT**
Percent Fat	34,853	−0.052 (−0.15, 0.05)	0.31	Body Fat Percent
Fasting Glucose (mmol/L)	46,186	0.011 (−0.03, 0.05)	0.58	MAGIC
**Fasting Insulin** ** **(pmol/L)**	**38,238**	**0.05 (0.01, 0.09)**	**1.5E-02**	**MAGIC**
HomaB	36,466	0.033 (0, 0.07)	5.1E-02	MAGIC
**Homa IR**	**37,037**	**0.042 (0, 0.08)**	**4.7E-02**	**MAGIC**
**2hr Glucose** ** **(mmol/L)**	**15,234**	**0.245 (0.06, 0.44)**	**1.1E-02**	**MAGIC**
HbA1C (%)	35,908	−0.002 (−0.04, 0.03)	0.91	MAGIC
**TG** ** **(SD units)**	**93,440**	**0.248 (0.18, 0.31)**	**2.6E-14**	**GLGC**
**HDL-C** ** **(SD units)**	**96,748**	**−0.243 (−0.31, −0.18)**	**4.5E-13**	**GLGC**
LDL-C (SD units)	92,348	0.023 (−0.05, 0.09)	0.52	GLGC
TC (SD units)	97,021	0.0003 (−0.07, 0.07)	0.99	GLGC

T2D: Type 2 diabetes, BMI: Body mass Index, WHR: Waist to hip ratio, HbA1C: hemoglobin A1C, TG: Triglyceride, HDL-C: High Density Lipoprotein Cholesterol, LDL-C: Low Density Lipoprotein Cholesterol, TC: Total Cholesterol.

**§:** Effect is mean change in trait or disease per adiponectin-decreasing allele.

*: Waist to hip ratio adjusted for BMI.

**: Significantly associated trait is coded in bold.

## Discussion

In this comprehensive multi-ethnic analysis of the genetic influences on adiponectin levels and their impact on metabolic traits and T2D, we have identified 10 novel loci and confirmed the associations of variants in the *ADIPOQ* and *CDH13* loci with adiponectin levels. The adiponectin risk alleles were associated with T2D and related metabolic traits such as BMI, WHR, TG, HDL-C, 2-hour glucose, HOMA-IR and fasting insulin. These findings demonstrate that adiponectin, T2D and metabolic syndrome have a shared allelic architecture.

### Biological Relevance of the GWAS Loci

In the first step toward understanding the biological relevance of the identified regions, we examined the genes harbored by the associated loci using human disease and animal databases. Although some of the genes in these loci do not have a known function, several signify diverse biological functions.

On chromosome 1, the lead SNP was located 300 kb from the *LYPLAL1*, a protein that regulates phospholipids on cellular membranes. Independent efforts have also identified this locus in other metabolic/obesity related traits GWAS: first with WHR (rs2605100; *r* ^2^ = 0.49 [21] and rs4846567; *r* ^2^ = 0.55 [27] respectively with the lead adiponectin SNP, rs3001032), and more recently with fasting insulin by a joint meta-analysis including the interaction between SNP and BMI (MF Hivert for the MAGIC investigators, personal communication). In the same report by MAGIC, variants near *IRS1* (insulin receptor substrate 1) and *PEPD* (a protein that hydrolyzes dipeptides and tripeptides) have also been associated with fasting insulin at genome wide significant levels, demonstrating a close link between adiponectin regulation and insulin resistance pathways. Moreover, both *IRS1* and *PEPD* have been associated with T2D (*IRS1* in DIAGRAM [28] and *PEPD* in a Japanese population [29]; *p* = 9.3×10^−12^ and *p* = 1.4×10^−5^, respectively).

The lead SNP at 3p21.1 falls within *GNL3* that is located in a genomic region containing many genes which could have potential functions in metabolism. Our data provide evidence that adiponectin levels were correlated with human adipocyte mRNA levels of many genes in this region (*GLYCTK*, *SEMA3G*, *STAB1*, *PBRM1*, *SFMBT1*; see Table 4). However, this association does not imply a direct influence of these genes on adiponectin level. Among those genes, *STAB1* encodes for stabilin 1, described as an endocytic receptor for advanced glycation end products and may have a function in angiogenesis, lymphocyte homing, cell adhesion, or receptor scavenging for acetylated low-density lipoprotein [30].

Interestingly, several of the genes near lead genome-wide significant SNPs have been implicated in angiogenesis, which might be important for adipose tissue expansion, highlighting the recurring theme of “adipose tissue expandability” in the genetic origins of obesity-related complications [31]. For example, *VEGFA* is the vascular endothelial growth factor A gene, a known gene in a variety of vascular endothelial cell functions, such as angiogenesis and maintenance of the glomerular endothelium in nephrons [32]. Variants in this gene are also associated with diabetic retinopathy and WHR [27], [33]. Moreover, the product of *VEGFA* interacts with resveratrol, which has been shown to have a beneficial influence in some metabolic traits, including diabetes [34]. Rodent studies show that resveratrol decreases blood glucose, blood insulin, and glycated hemoglobin, as well as increases insulin sensitivity in animals with hyperglycemia (reviewed in [35]). Resveratrol also inhibits TNF-α-induced reductions in adiponectin levels in 3T3-L1 adipocytes [36]. Furthermore, it has been shown that resveratrol modulates adiponectin expression and improves insulin sensitivity, likely through the inhibition of inflammatory-like response in adipocytes [37]. At this locus, *VEGFA* mRNA levels in adipocytes were the strongest association with adiponectin levels (Table 4). Also likely involved in vascular biology, *TRIB1* encodes a G protein-coupled receptor-induced protein interacting with MAP kinases that regulates proliferation and chemotaxis of vascular smooth muscle cells [38]. *TRIB1* expression was shown to be elevated in human atherosclerotic arteries [39]. Several variants (rs2954029, rs2954021, rs17321515; all in moderate LD with our lead SNP) in the *TRIB1* gene have been associated with HDL-C, LDL-C and CHD risk in European and Asian populations [22], [40], [41], [42], [43]. These two loci (*TRIB1* and *VEGFA*) argue for the importance of vascular biology in adiponectin regulation as underlined previously by findings of adiponectin levels associated with variants near *CDH13* (a receptor for adiponectin expressed by endothelial smooth muscle) [44].

All three homologous genes *GPR109A/B/81* located on chromosome 12 are predominantly expressed in adipocytes and mediate antilipolytic effects [45]. Our eQTL results (Table 3) and the correlation between mRNA and adiponectin levels (Table 4) argue strongly for a role of *GPR109A* at this locus. *GPR109A* (also known as *NIACR1*) is a receptor with a high-affinity, concentration-dependent response to nicotinic acid (niacin) [45]. Treatment by niacin increases serum adiponectin levels by up to 94% in obese men with metabolic syndrome in a time- and dose-dependent manner [46]. Functional studies in *GPR109A* receptor knockout mice demonstrate that niacin increases serum total and HMW adiponectin concentrations and decreases lipolysis following *GPR109A* receptor activation [47]. Moreover, a recent meta-analysis on cohorts containing extremes of HDL-C provided evidence suggestive of association in *GPR109A/B/81* [48].

Finally, variants in *ZNF664* have been associated with CHD, HDL-C and TG levels in a large meta-analysis of over 100,000 individuals of European ancestry [22]. The sex heterogeneity observed in this study is comparable to our finding that the more loci associated with adiponectin at genome wide significance level have been shown in female stratified analysis.

Taken together, the loci identified in this large-scale GWAS for adiponectin levels highlight many genes with demonstrated relationships with metabolic disease.

### Shared Allelic Architecture of Adiponectin Levels and Metabolic Traits

Using a multi-SNP genotypic risk score we attempted to understand if the allelic architecture of adiponectin levels was shared with T2D and metabolic traits. This risk score was associated with increased risk of T2D and traits associated with insulin resistance and the metabolic syndrome. However, unexpectedly, adiponectin decreasing alleles were associated with a decrease in BMI. In our adiponectin GWAS, BMI was included as a covariate in order to avoid direct identification of obesity SNPs since BMI is strongly related to adiponectin levels [49], [50]. Furthermore, this unexpected direction of effect was entirely explained by SNPs at the *ZNF664* and *PEPD* loci; when these loci were removed from the analysis, the association of the genotypic risk score with BMI disappeared (results not shown). Therefore, adiponectin risk alleles at *ZNF664* and *PEPD* are of considerable interest since they impart deleterious changes on aspects of the metabolic syndrome (increased TC, TG, LDL-C and WHR and decreased HDL-C), but also act to decrease BMI and percent fat.

Our data do not provide direct evidence as to whether the genetic determinants of adiponectin levels influence these traits through adiponectin itself, or through pleiotropic pathways and therefore do not constitute a Mendelian randomization study. These findings provide a note of caution for Mendelian randomization studies, which may be prone to erroneous conclusions if pleiotropic effects of tested variants are not considered. Nonetheless, in aggregate, these results provide strong evidence that the genetic determinants of adiponectin levels are shared with metabolic disease, and in particular, traits related to insulin resistance.

We note that there are several strengths and limitations of this study. Our main findings, identifying genetic determinants of adiponectin levels, are based on the largest meta-analysis to date and include results from three ethnicities. The availability of expression data from human adipose tissue permitted the association of identified SNPs with mRNA levels at candidate genes and, in turn, correlation of these mRNA levels with circulating adiponectin itself. While access to the data from large consortia permitted assessment of the relevance of the identified SNPs to T2D and components of the metabolic syndrome, we note that a subset of the cohorts included in our GWAS were also included in these external consortia. However, we note that even if we assume that all ADIPOGen study participants were included in the external consortia, for cohorts participating in both studies, that the majority of data in these external consortia still arises from study participants not present in ADIPOGen (minimum percent of non-overlapping subects: 86.8%, 85.5%, 86.4% and 82.5% for MAGIC, GLGC, GIANT, and DIAGRAM+ consortia, respectively). Therefore, since a substantial majority of participants are independent between ADIPOGen and these consortia, it is unlikely that our findings demonstrating a shared allelic architecture between adiponectin levels and these traits are spurious.

Further, we suggest that locus, 6q24.1, identified only through multi-ethnic meta-analysis using MANTRA and not confirmed through fixed and random effects meta-analysis, be replicated for confirmation of this finding.

In conclusion, the data presented in this study provide strong evidence of association for 10 novel loci for adiponectin levels. Further analyses confirmed that the level of expression of some of these candidate genes in human adipocytes correlated directly with adiponectin levels. A multi-SNP genotypic risk score, and several of the identified variants, directly influence parameters of the metabolic syndrome and, in particular, markers of insulin resistance. These findings identify novel genetic determinants of adiponectin levels, which, taken together, influence risk of T2D and markers of insulin resistance.

## Materials and Methods

### Ethical Consideration

All participants provided informed written consent. The research protocol of all studies were reviewed and approved by institutional ethics review committees at the involved institutions.

### Study Design

**Figure 3:**
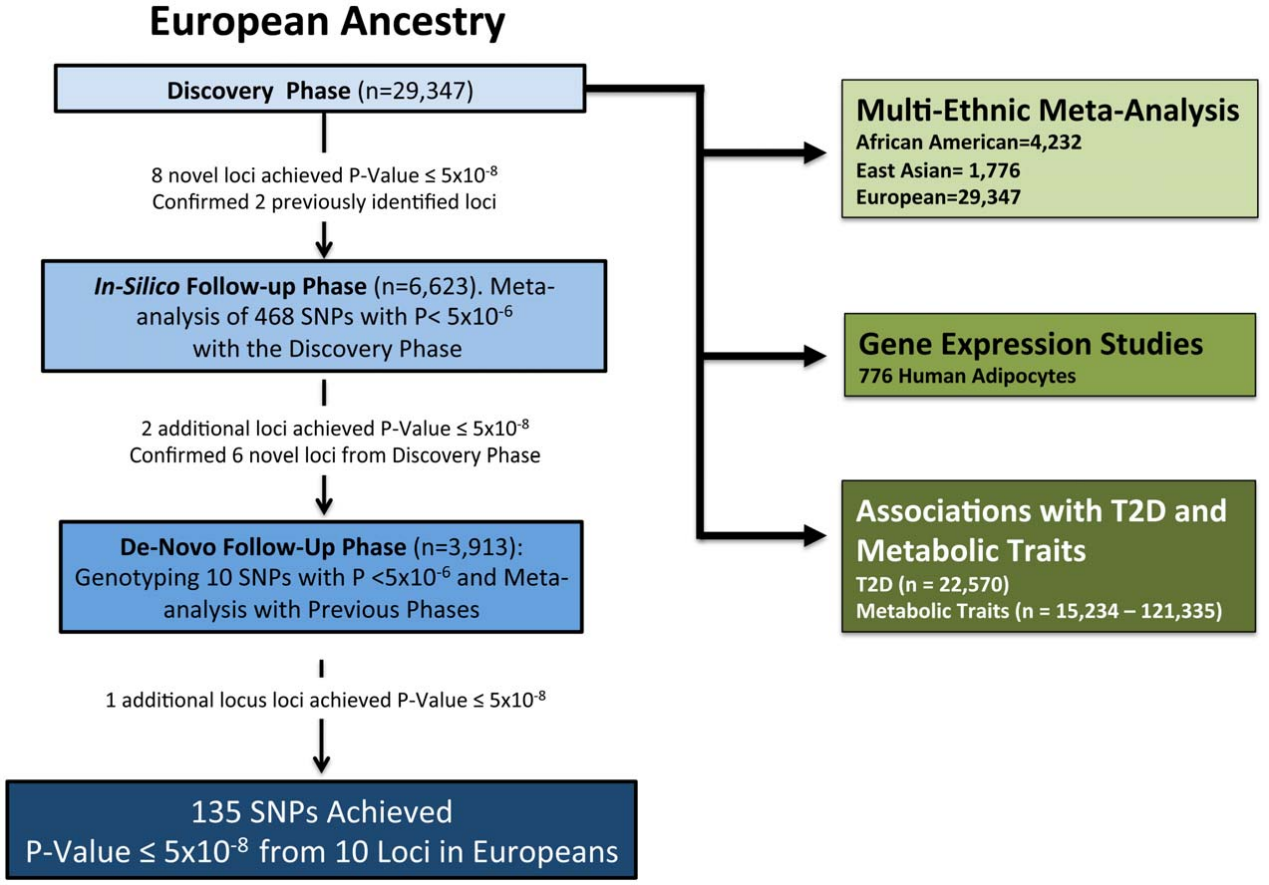
Flow chart of study design.

Our study consisted of three stages. *First*, in the discovery stage we performed a meta-analysis of the GWAS summary statistics of 16 studies involving 29,347 participants of white European origin to detect SNPs that are associated with adiponectin levels. All signals with p<5×10^−6^ were followed up in seven additional cohorts (n = 6,623) with GWAS data (*in-silico* phase) that later joined the consortium and then a subset of SNPs (n = 10) by *de-novo* genotyping in 3,913 additional participants from three cohorts (n = 39,883 for the combined analysis in Europeans). We also performed a multi-ethnic meta-analysis by combining summary statistics from the 16 studies of individuals of white European discovery cohorts (n = 29,347) with those of five cohort studies that included African Americans subjects (n = 4,232) and one East Asian cohort (n = 1,776) to obtain a total 35,355 individuals for the GWAS meta-analysis involving different ethnicities. After identifying variation near two genes of pharmaceutical importance (*GPR109A* and *GPR109B*), which encode the putative niacin receptors, we typed additional rare coding and tagging variants in a subset of cohorts. *Second*, we examined whether the identified SNPs of the first stage also associate with mRNA levels of nearest gene(s) expressed using adipose tissue of 776 European women. We also tested for association between adiponectin levels and mRNA levels of the genes in our candidate loci in adipose tissue of a subgroup of 436 individuals [25]. *Third*, we calculated a multi-SNP genotypic risk score using genome-wide significant adiponectin-lowering alleles and tested the association of this risk score with T2D and related metabolic traits. Figure 3 shows a flow chart detailing the study design.

### Study Populations

In total, 45,891 individuals from 26 European and 7 non-European cohorts participated in the different phases of this meta-analysis. Participating cohorts were either population-based (n = 23), family-based (n = 4), or case-control (n = 4) studies. The age of participants ranged from 10 to 95 years. Adiponectin levels were measured using ELISA or RIA methods. More details on the study cohorts and adiponectin measurement are presented in the Text S1 and Table S1. In addition, genotyping of four coding and tagging SNPs in the candidate genes, *GRP109A* and *GPR109B*, was undertaken in samples from the Lausanne, Lolipop, MRC Ely, and Fenland cohorts.

### Genotyping and Imputation

All cohorts were genotyped using commercially available Affymetrix or Illumina genome-wide genotyping arrays. Quality control was performed for each study independently and genotype imputation was carried out using IMPUTE, MACH, BimBam or Beagle with reference to either the Phase II CEU, CEU+YRI, or CHB+JPT+CEU HapMap according to the origin of population. Imputation of East Asian genotypes was undertaken by first masking genotypes of 200 SNPs and then imputing them based on the CEU+CHB+JPT panel from HapMap. This resulted in an allelic concordance rate of ∼96.7%. For the African Americans, a combined CEU+YRI reference panel was created. This panel included SNPs segregating in both CEU and YRI, as well as SNPs segregating in one panel and monomorphic and non-missing in the other (2.74 million SNPs). Due to the overlap of African American individuals on the Affymetrix 6.0 and IBC arrays [51], it was possible to analyze imputation performance at SNPs not genotyped on Affymetrix 6.0. For imputation based on Affymetrix data, the use of the CEU+YRI panel resulted in an allelic concordance rate of ∼95.6% (calculated as 1−0.5 * [imputed_ dosage– chip_dosage]). This rate is comparable to rates calculated for individuals of African descent imputed with the HapMap 2 YRI individuals. Table S1 summarizes the genotyping methods used for each cohort, genotype-calling algorithms, imputation algorithms and exclusion thresholds. SNP-level quality control metrics were applied prior to meta-analysis for each cohort. These were: call rate ≥95%, minor allele frequency (MAF)≥1%, Hardy-Weinberg equilibrium (HWE) *p*>10^−6^, and quality measures for imputed SNPs (r^2^≥0.3, or proper info ≥0.4, for cohorts imputing their data with MACH and IMPUTE, respectively).

Eleven coding and tagging variants in two candidate genes of pharmaceutical importance (*GPR109A* encoding the niacin receptor and *GPR109B*) were genotyped in a parallel study in Lausanne, Lolipop, MRC Ely, and Fenland white subjects. Genotyping was performed using a KASPar-On-Demand SNP Genotyping Assay (KBioscience Ltd., Hoddesdon, UK). In Lausanne and Lolipop samples the genotyping assay was carried out on 3.75 ng of genomic DNA in 1 µl 1536-well plate reactions, dispensed with a Meridian, microfluidic dispenser (KBioscience Ltd., Hoddesdon, UK), thermocycled using a Hydrocycler (KBioscience Ltd., Hoddesdon, UK). A Pherastar (BMG GmbH, Germany) was used for end-point detection and Kraken-LIMS (KBioscience Ltd., Hoddesdon, UK) was used for automated allele calling. In MRC Ely and Fenland samples, the genotyping assay was carried out on 10 ng of genomic DNA in 5 µl 384-well plate reactions using a G-Storm GS4 Thermal Cycler (GRI, Rayne, UK). The ABI PRISM 7900HT Sequence Detection System (Applied Biosystems, Warrington, UK) was used for end-point detection and allele calling.

### Statistical Analysis

#### Genome-wide association studies

All cohorts independently tested for the additive genetic association of common (MAF>1%) genotyped and imputed SNPs with natural log transformed adiponectin levels, while adjusting for age, sex, body mass index (BMI), principal components of population stratification and study site (where appropriate), and for family structure in cohorts with family members [49], [50], [52]. The analyses were performed for men and women combined, as well as for men and women separately. The Cardiovascular Health Study cohort (CHS) also provided GWA results for high molecular weight (HMW) adiponectin using the same methods as described above.

#### Meta-analysis of GWAS

The meta-analysis was performed by two analysts independently each using different methods; inverse variance-weighted methods using both fixed and random effect models available through either the METAL (http://www.sph.umich.edu/csg/abecasis/metal/) or GWAMA version 2.0.5 (http://www.well.ox.ac.uk/gwama/) software packages [53]. Summary statistics were crosschecked to ensure consistency of results. Prior to the meta-analysis, study-specific summary statistics were corrected using genomic control (lambda range = 0.99–1.25) and the overall meta-analytic results were additionally corrected for genomic control (lambda = 1.06). To examine whether associations with adiponectin were sex-specific, we performed meta-analyses for men and women separately. A *p*-value threshold of 5×10^−8^ was considered to be genome-wide significant. Ethnicity-specific meta-analyses were performed for white European and non-European populations separately, using the same methods as described above.

Presence of heterogeneity in the meta-analysis was assessed by the I^2^ statistic and Q-test [54]. Since cohorts measured adiponectin concentrations using either RIA or ELISA methods, we also performed a GWA meta-analysis stratified by the method of measurement to test whether this contributed to heterogeneity.

#### Follow-up phase

The follow-up phase comprised two stages; *in-silico follow-up* and *de-novo follow-up*.

##### In silico follow-up

468 SNPs with *p*<5×10^−6^ from the discovery phase (which includes both genome-wide significant [n = 196, p<5×10^−8^] and “suggestive” [n = 272, 5×10^−8^<*p*<5×10^−6^] SNPs Table S3) were tested for their association in 6,623 individuals from seven additional cohorts with GWAS data that joined the consortium after the discovery stage had been finalized.

##### De novo follow-up

We next selected the lead SNP arising from selected loci from the joint analysis of the discovery and *in-silico* follow-up phase with p-values greater than 5×10^−8^ but less than 5×10^−6^ and genotyped 10 SNPs in 3,164 samples from the SAPHIR cohort and an additional subgroup of the KORA cohort. Finally, these same SNPs, or their proxy SNPs (n = 2), were tested for association in the THISEAS cohort (n = 738), which had been genotyped using the Metabochip [55]. Study-level summary statistics from the follow-up phases were meta-analyzed with the data from the discovery phase.

#### Multi-ethnic meta-analysis

In order to perform a meta-analysis of GWAS data from cohorts of different ethnic backgrounds, we utilized the novel MANTRA (Meta-ANalysis of Trans-ethnic Association studies) software [24]. This method combines GWAS from different ethnic groups by taking advantage of the expected similarity in allelic effects between the most closely related populations. Fixed-effects meta-analysis assumes the allelic effect to be the same in all populations, and cannot account for heterogeneity between ethnic groups. Conversely, random effects meta-analysis assumes that each population has a different underlying allelic effect, however, populations from the same ethnic group would be more homogeneous than those that are more distantly related. To address this challenge we accounted for the expected similarity in allelic effects between the most closely related populations by means of a Bayesian partition model. For each variant, allelic effects and corresponding standard errors are estimated within each population under the assumption of an additive model. Populations are then clustered according to their similarity in terms of relatedness as measured by the mean allele frequency difference at 10,000 independent SNPs, and to their allelic effects at the variant. If all populations are assigned to the same cluster, this is equivalent to a fixed allelic effect across all populations (i.e. no trans-ethnic heterogeneity). The posterior distribution of the allelic effect in each population under the Bayesian partition model is approximated by means of a Monte-Carlo Markov chain algorithm. Evidence in favor of association of the trait with the variant was assessed by means of a Bayes' factor (BF). A log10 BF of 6 or higher is considered a relatively conservative threshold for genome-wide significance. We also performed meta-analysis by using both random and fixed effects models including all ethnicities. Those loci that achieved both a BF>6 in MANTRA and a P-value less than 5×10^−7^ in multiethnic analysis are presented in Table 2.

### Association of Genome-Wide Significant SNPs with Gene Expression (Stage 2)

In order to identify *cis*-expression quantitative trait loci (*cis*-eQTLs) and test whether mRNA levels of candidate genes arising from our GWAS were associated with adiponectin levels, we used expression profiles in human adipocytes from the Multiple Tissue Human Expression Resource (MuTHER) Consortium, (856 female twins from the UK) [25]. mRNA expression profiles from subcutaneous fat and genome-wide genotypes were available for 776 individuals and circulating adiponectin levels for 436 of these women. We note that while adiponectin levels were measured at an earlier time point than the fat biopsies, the BMI at time of adipose expression measurement and time of adiponectin measurement was highly correlated (r^2^ = 0.9).

*cis*-eQTLs were defined as associations between SNPs and a transcript within 1 Mb of the identified SNP. To correct for multiple testing, we used QVALUE software [56], and estimated that a genome-wide false discovery rate of 1% corresponds to a *p*-value threshold of 5.06×10^−5^ (this conservative threshold accounts for all multiple arising from the use of the array, rather than multiple testing arising from assessing only transcripts in the genome-wide significant regions). To test whether mRNA levels of candidate genes identified in the GWAS meta-analysis are associated with circulating adiponectin levels, we applied a Bonferoni corrected threshold of *p*<3×10^−4^ (where 3×10^−4^ = 0.05/133 and 133 was the number of transcripts tested at the candidate loci).

### Association of Genome-Wide Significant SNPs with T2D and Metabolic Traits (Stage 3)

The DIAGRAM+ (effective n = 22,044) [19], MAGIC (n = up to 46,186) [20], GLGC (n = up to 97,021) [22], GIANT (n = up to 121,335) [21], and Body Fat GWAS (n = up to 36,625) consortia provided summary statistics for the association of each SNP that was genome-wide significant in the discovery phase. Since 196 SNPs (which were estimated to be equivalent to 96 independent statistical tests due to linkage disequilibrium [LD]) [26] were tested for their association, we employed a Bonferroni-corrected threshold of α = 0.0005 (where 0.0005 = 0.05/96) to define the threshold of association for any individual SNP association with T2D and related traits.

While any individual SNP may demonstrate a relationship with T2D or related traits, it can be more informative to test whether a multi-SNP genotypic risk score is associated with the outcome of interest. In the absence of pleiotropic effects arising from loci other than *ADIPOQ*, such a multi-SNP genotypic risk score would enable testing of whether adiponectin levels are causally related to risk of T2D or metabolic traits through a Mendelian randomization framework. Since most of the SNPs that we identified to be genome-wide significant for adiponectin levels were not in the *ADIPOQ* locus, the presence of such pleiotropy precluded a formal Mendelian randomization study. To create a multi-SNP genotypic risk score we implemented a novel method that approximates the average effect of adiponectin decreasing alleles on T2D or related traits. Further, this method allows the use of consortium-level meta-analytic results for a set of SNPs, rather than requiring the re-analysis of individual-level data in each cohort, thereby providing more accurate effects of each allele (due to the larger sample size in the consortium-level meta-analysis). The weighted sum of the individual SNP coefficients leads not only to an estimate of the average combined allelic effect, but also to an approximate estimate of the explained variance (when scaled by the inverse of the total meta-analysis sample size) from a multivariate regression model containing these SNPs.

Specifically, suppose *m* SNPs have shown association in the discovery phase, and effects are denoted *w_i_*. However, suppose that the goal of interest is to estimate the joint effect of these SNPs on an outcome of interest, *y*. Let *j* index the individuals in the outcome of interest dataset and let
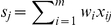
be a risk score based on the discovery data SNPs, and their associated parameter estimates *w_i_*. Therefore, the desired goal is to estimate the parameter in the following equation: 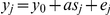 in the outcome of interest dataset. The proportion of variance in *y* explained by the previous equation, (i.e. the R^2^) attributable to the risk score can be estimated. Standard linear model theory shows that the change in log likelihood is proportional to the R^2^,
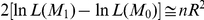
If the SNPs are uncorrelated, and if the total percentage of variance explained is small, then the change in log likelihood can be approximated by
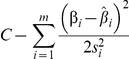
where β_i_ now refers to the effect of SNP *i* in the outcome data, 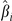 is the outcome data estimate, and *s_i_* is the associated standard error estimate. Assuming that this log likelihood difference approximation is maximized with an appropriate value of C, then it can be shown that *a* can be estimated by:
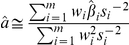
with a standard error estimate of
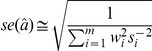
Therefore, under the assumption of uncorrelated SNPs, their joint effect can be estimated in external data by a weighted mean of the individual SNP effects, weighted by the estimates from the discovery data. All these quantities can be obtained from meta-analysis or summary data, so that individual-level data are not required to obtain these results.

To implement this method, we first selected LD-independent adiponectin associated alleles by LD pruning the set of genome-wide significant adiponectin SNPs from the discovery phase with an LD threshold of r^2^≤0.05 in the HapMap CEU population, yielding 20 independent LD blocks from the 196 SNPs in Table S2. (We also applied the method using an LD threshold of r^2^≤0.01 and found no relevant change in results). Since many SNPs from the same independent blocks were associated with adiponectin, we selected the SNP from the LD block that explained the most variance in adiponectin levels. Next, we approximated the effect of the multi-SNP genetic risk score using *β* and its standard error as derived from the consortium-level meta-analysis in DIAGRAM+, MAGIC, GLGC, GIANT and Body Fat GWAS consortium.

## Supporting Information

Figure S1 The comparison between two independent meta-analyses performed in different centers for quality control purposes. The −log10 *p*-value of all SNPS with MAF≥0.01 in the first analysis are plotted against the −log10 *p*-value from the second analysis.
(TIF)

Figure S2 The Manhattan plots of sex-stratified meta-analyses in the discovery phase in the European population. The meta-analysis shown in panel a) is stratified for women and that in panel b) is stratified for men. Manhattan plots demonstrate −Log _10_(*p*-value) measures for association between single nucleotide polymorphisms (SNPs) and chromosomal position. The SNPs that achieved genome-wide significance are highlighted in green in the plots. The red ovals identify loci found only in women.
(TIF)

Figure S3 Association Results Near Peaks for Sex-specific Analysis of Adiponectin. SNPs in regions near peak associations are shown for a) chromosome 8 female, b) chromosome 8 males, c) chromosome 12 females and d) chromosome 12 males. Purple diamonds indicate the top SNPs, which have the strongest evidence of association in women. Each circle shows a SNP with a color scale proportional to the r^2^ value for that SNP and the top SNP from HapMap CEU. Blue lines show the estimated recombination rates from HapMap. The bottom panels illustrate the relative position of each gene in the locus.
(TIF)

Table S1 Cohort characteristics.
(XLSX)

Table S2 Comparing the Genome-Wide Significant SNPS from fixed effect model with random effect model. *SNP with I^2^ less than 0.5 are listed in bold, EA: Effect Allele, NEA: Non-Effect Allele.
(PDF)

Table S3 Association Results of SNPs achieving *p*≤5×10^−6^ in the Discovery phase in European Populations (Sex-Combined Analysis). *Denotes SNPs typed in the *de-novo* follow-up phase.
(PDF)

Table S4 Genome-Wide Significant SNPs (p<5×10^−8^) Associated with Adiponectin Levels in Non-Europeans Populations. EA: Effect Allele, NEA: Non-Effect Allele, EA-Freq: Frequency of Effect Allele.
(PDF)

Table S5 SNPs associated with adiponectin at genome-wide significant levels (p<5×10^−8^) using the fixed-effect model in women only in European populations (including Discovery and Follow-Up phases).
(PDF)

Table S6 SNPs associated with adiponectin at genome-wide significant levels (p<5×10^−8^) using fixed-effect models in men only in Euopean populations.
(PDF)

Table S7 Association results of nominally significant SNPs with Type 2 Diabetes in the DIAGRAM+ Consortium. EA: Effect Allele, NEA: Non-Effect Allele. B) Association results of nominally significant SNPs with diabetes-related traits in the MAGIC Consortium. Fasting glucose and 2 h glucose in mmol/L; Insulin in pmol/L, EA: Effect Allele, NEA: Non-Effect Allele. C) Association results of nominally significant SNPs with diabetes-related traits in the GIANT and Body fat GWAS consortia. The beta expressed in inverse normally transformed BMI units (i.e. interpretable as SD or Z-score), shows the change in BMI per additional effect allele.,*Results that are statistically significant, accounting for the number of independent SNPs, are highlighted in bold., EA: Effect Allele, NEA: Non-Effect Allele, EA-Freq: Frequency of Effect Allele. D) Association results of nominally significant SNPs with lipid traits in the GLGC Consortium. For these traits the effect size is in SD units, based on standard error-weighted meta-analysis. *Results that are statistically significant, accounting for the number of independent SNPs are highlighted in bold., EA: Effect Allele, NEA: Non-Effect Allele, EA-Freq: Frequency of Effect Allele.
(PDF)

Text S1 Supplemental data include description of study cohorts and funding.
(DOCX)
