# Supplementary figures and images for: Novel Loci for Adiponectin Levels and Their Influence on Type 2 Diabetes and Metabolic Traits: A Multi-Ethnic Meta-Analysis of 45,891 Individuals
Source: PLoS Genet. 2012 Mar 29;8(3):e1002607. doi: 10.1371/journal.pgen.1002607 (PMC3315470; doi:10.1371/journal.pgen.1002607)

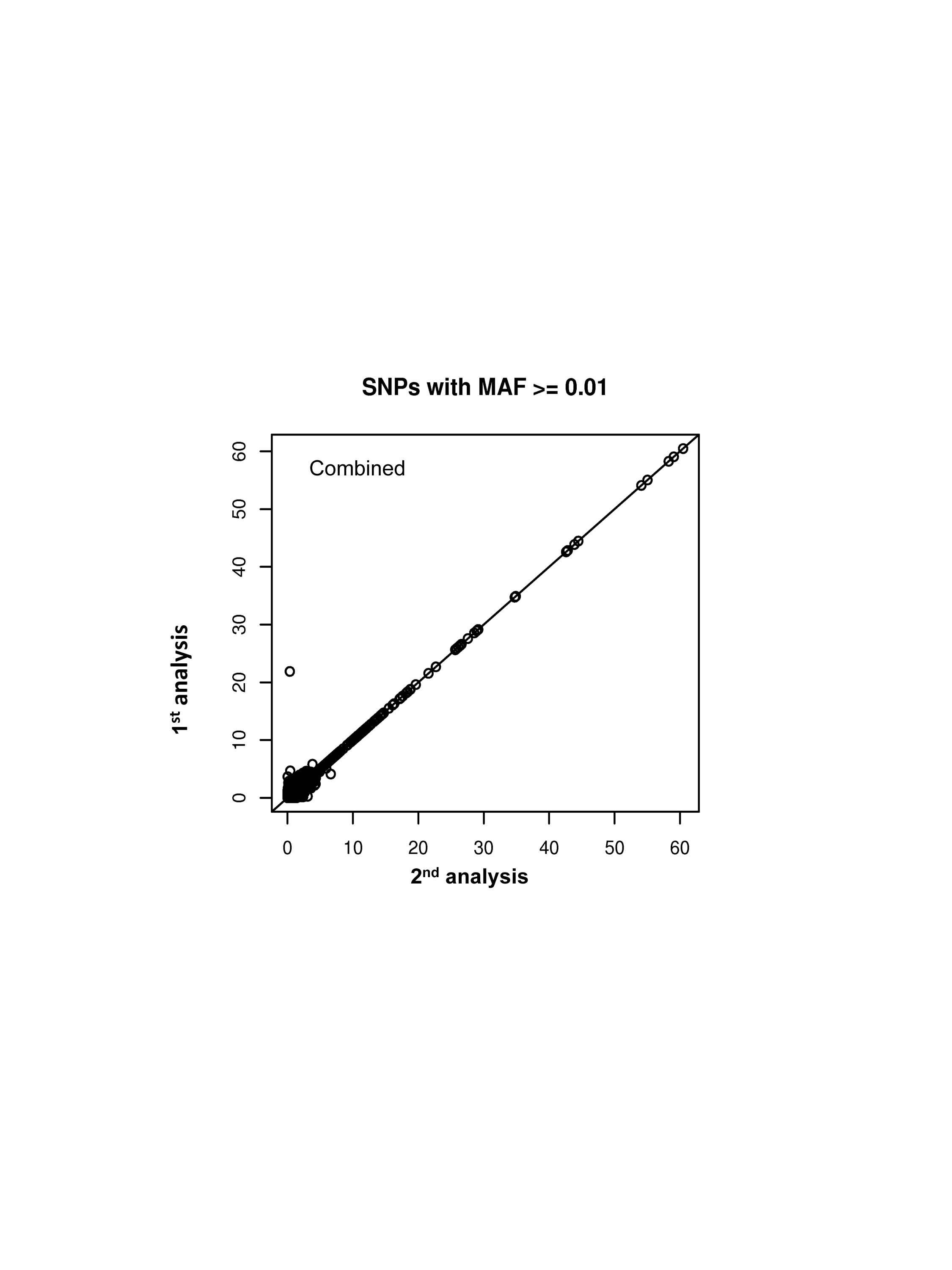

Supplement: Figure S1 — The comparison between two independent meta-analyses performed in different centers for quality control purposes. The −log10 p-value of all SNPS with MAF≥0.01 in the first analysis are plotted against the −log10 p-value from the second analysis. (TIF) [file pgen.1002607.s001.tif]

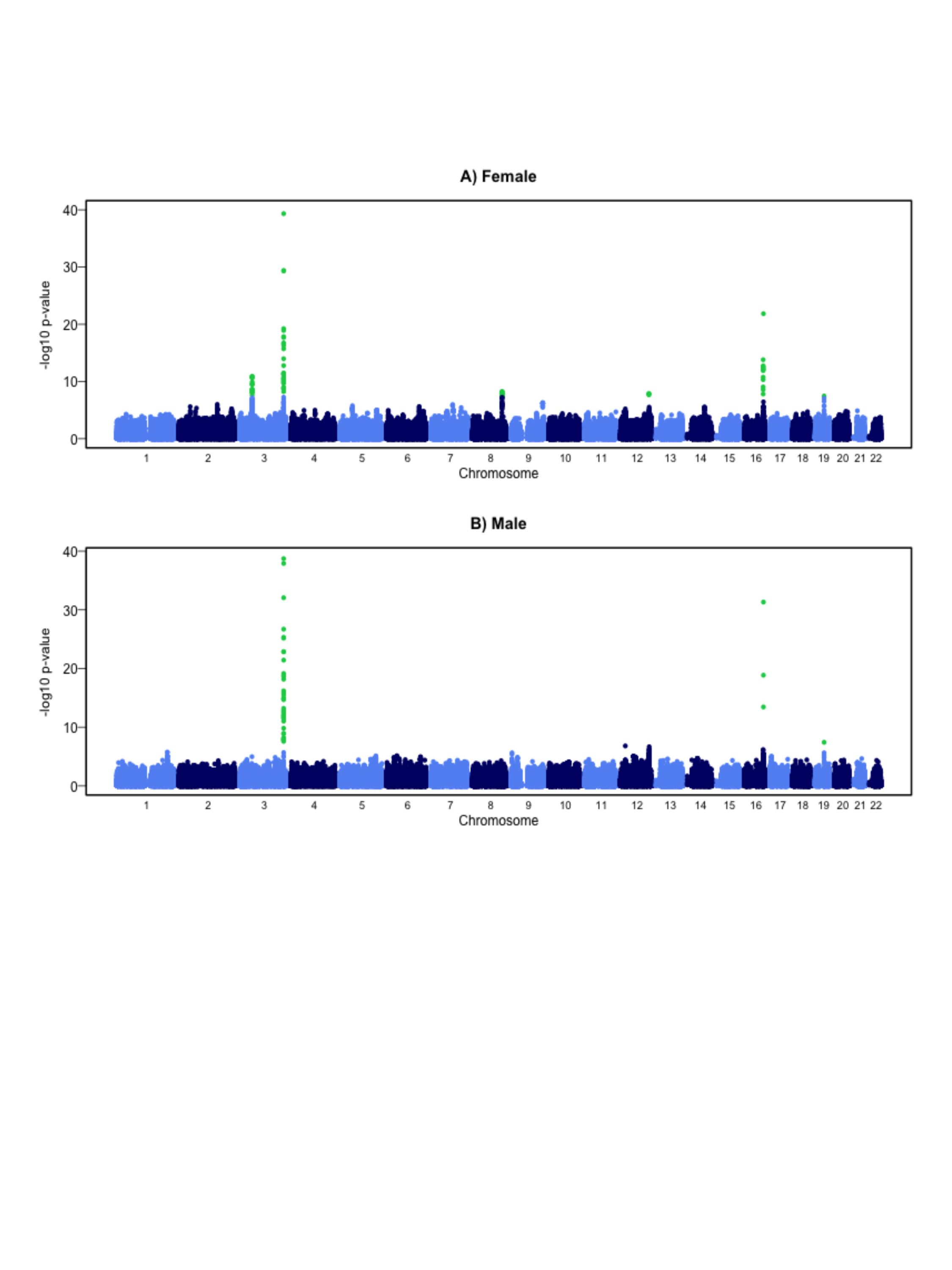

Supplement: Figure S2 — The Manhattan plots of sex-stratified meta-analyses in the discovery phase in the European population. The meta-analysis shown in panel a) is stratified for women and that in panel b) is stratified for men. Manhattan plots demonstrate −Log 10(p-value) measures for association between single nucleotide polymorphisms (SNPs) and chromosomal position. The SNPs that achieved genome-wide significance are highlighted in green in the plots. The red ovals identify loci found only in women. (TIF) [file pgen.1002607.s002.tif]

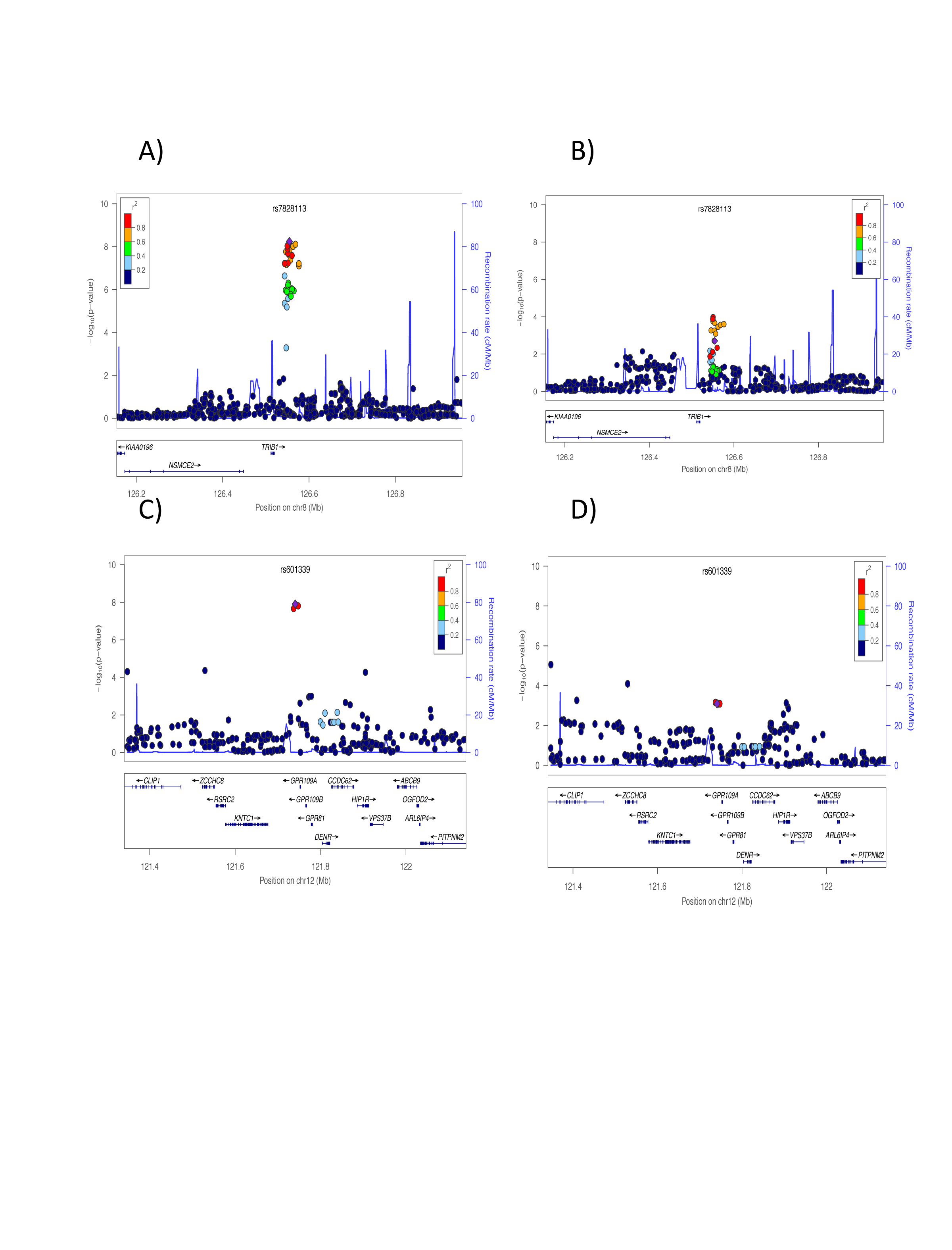

Supplement: Figure S3 — Association Results Near Peaks for Sex-specific Analysis of Adiponectin. SNPs in regions near peak associations are shown for a) chromosome 8 female, b) chromosome 8 males, c) chromosome 12 females and d) chromosome 12 males. Purple diamonds indicate the top SNPs, which have the strongest evidence of association in women. Each circle shows a SNP with a color scale proportional to the r2 value for that SNP and the top SNP from HapMap CEU. Blue lines show the estimated recombination rates from HapMap. The bottom panels illustrate the relative position of each gene in the locus. (TIF) [file pgen.1002607.s003.tif]
