# Supplementary material for: Novel Loci for Adiponectin Levels and Their Influence on Type 2 Diabetes and Metabolic Traits: A Multi-Ethnic Meta-Analysis of 45,891 Individuals
Source: PLoS Genet. 2012 Mar 29;8(3):e1002607. doi: 10.1371/journal.pgen.1002607 (PMC3315470; doi:10.1371/journal.pgen.1002607)
Supplement: Table S2 — Comparing the Genome-Wide Significant SNPS from fixed effect model with random effect model. *SNP with I2 less than 0.5 are listed in bold, EA: Effect Allele, NEA: Non-Effect Allele. (PDF) [file pgen.1002607.s005.pdf]

Table S2**: Comparing the Genome-Wide Significant SNPS from fixed effect model with random effect model.**

				Discovery-fixed Effect		Discovery-Random Effect			
SNP	Chr/Pos	EA/NEA	EAF	Beta (SE)	P-Value	Beta (SE)	P-Value	Q-PValue	I^2
**rs13081028**	**3/52530356**	**G/A**	**0.43**	**-0.03(0.004)**	**1.05E-09**	**-0.03(0.006)**	**1.46E-05**	**0.07**	**0.4**
**rs9853056**	**3/52530997**	**T/C**	**0.43**	**-0.03(0.004)**	**9.37E-10**	**-0.03(0.006)**	**1.31E-05**	**0.07**	**0.4**
**rs4282054**	**3/52541105**	**T/C**	**0.43**	**-0.03(0.004)**	**6.25E-10**	**-0.03(0.006)**	**9.13E-06**	**0.08**	**0.4**
**rs12489828**	**3/52542054**	**T/G**	**0.55**	**0.03(0.004)**	**5.49E-08**	**0.02(0.006)**	**3.24E-04**	**0.03**	**0.4**
**rs2590838**	**3/52597126**	**G/A**	**0.49**	**-0.03(0.004)**	**7.05E-11**	**-0.03(0.006)**	**2.48E-06**	**0.10**	**0.3**
**rs2276824**	**3/52612526**	**G/C**	**0.57**	**0.03(0.005)**	**3.06E-10**	**0.03(0.006)**	**6.07E-06**	**0.10**	**0.3**
**rs9879090**	**3/52623305**	**T/C**	**0.43**	**-0.03(0.005)**	**2.68E-10**	**-0.03(0.006)**	**5.98E-06**	**0.10**	**0.3**
**rs13083798**	**3/52624788**	**G/A**	**0.51**	**0.03(0.004)**	**5.18E-11**	**0.03(0.006)**	**2.51E-06**	**0.10**	**0.3**
**rs1108842**	**3/52695120**	**C/A**	**0.51**	**0.03(0.004)**	**3.66E-11**	**0.03(0.006)**	**2.06E-06**	**0.10**	**0.3**
**rs11235**	**3/52720127**	**T/C**	**0.57**	**0.03(0.005)**	**2.80E-10**	**0.03(0.006)**	**5.76E-06**	**0.10**	**0.3**
**rs2710323**	**3/52790945**	**T/C**	**0.51**	**-0.03(0.004)**	**2.90E-10**	**-0.03(0.005)**	**9.07E-07**	**0.17**	**0.3**
**rs3617**	**3/52808845**	**C/A**	**0.54**	**-0.03(0.004)**	**2.20E-08**	**-0.03(0.005)**	**7.29E-07**	**0.31**	**0.1**
**rs4481150**	**3/52812833**	**T/C**	**0.53**	**-0.03(0.004)**	**3.33E-08**	**-0.02(0.006)**	**2.70E-05**	**0.16**	**0.3**
**rs2535627**	**3/52820145**	**T/C**	**0.53**	**-0.03(0.004)**	**2.54E-08**	**-0.02(0.006)**	**3.07E-05**	**0.14**	**0.3**
**rs2071044**	**3/52822641**	**T/C**	**0.47**	**0.03(0.004)**	**3.65E-08**	**0.02(0.006)**	**3.51E-05**	**0.14**	**0.3**
**rs7636643**	**3/151532470**	**T/A**	**0.09**	**-0.04(0.008)**	**2.77E-08**	**-0.04(0.008)**	**2.77E-08**	**0.99**	**0.0**
**rs16862607**	**3/151533988**	**G/A**	**0.09**	**-0.04(0.008)**	**2.76E-08**	**-0.04(0.008)**	**2.76E-08**	**0.99**	**0.0**
**rs16862610**	**3/151535497**	**T/C**	**0.91**	**0.04(0.008)**	**2.55E-08**	**0.04(0.008)**	**2.55E-08**	**0.99**	**0.0**
**rs2060662**	**3/151536642**	**G/C**	**0.09**	**-0.04(0.008)**	**2.50E-08**	**-0.04(0.008)**	**2.50E-08**	**0.99**	**0.0**
**rs1597466**	**3/151538251**	**T/G**	**0.10**	**-0.04(0.008)**	**1.89E-08**	**-0.04(0.008)**	**1.89E-08**	**0.98**	**0.0**
**rs7616723**	**3/151538334**	**G/A**	**0.91**	**0.04(0.008)**	**2.62E-08**	**0.04(0.008)**	**2.62E-08**	**0.99**	**0.0**
**rs7617025**	**3/151538648**	**G/A**	**0.90**	**0.04(0.008)**	**2.26E-08**	**0.04(0.008)**	**2.26E-08**	**0.99**	**0.0**
**rs7646517**	**3/187902864**	**T/C**	**0.12**	**-0.04(0.007)**	**4.50E-10**	**-0.04(0.007)**	**4.50E-10**	**0.48**	**0.0**
**rs11927941**	**3/187915740**	**G/A**	**0.32**	**-0.03(0.005)**	**3.99E-10**	**-0.03(0.006)**	**1.21E-06**	**0.12**	**0.3**
**rs10937266**	**3/187915769**	**G/A**	**0.69**	**0.03(0.005)**	**1.21E-09**	**0.03(0.006)**	**5.36E-06**	**0.09**	**0.3**
**rs11924390**	**3/187915793**	**T/C**	**0.69**	**0.03(0.005)**	**1.23E-09**	**0.03(0.006)**	**5.69E-06**	**0.09**	**0.4**
**rs3774291**	**3/187916165**	**T/C**	**0.32**	**-0.03(0.005)**	**1.21E-09**	**-0.03(0.006)**	**5.46E-06**	**0.09**	**0.3**
**rs3774292**	**3/187916268**	**T/A**	**0.32**	**-0.03(0.005)**	**4.70E-09**	**-0.03(0.007)**	**1.10E-04**	**0.03**	**0.5**
**rs10440056**	**3/187916516**	**T/C**	**0.32**	**-0.03(0.005)**	**4.76E-09**	**-0.03(0.007)**	**1.11E-04**	**0.03**	**0.5**
**rs3821815**	**3/187916956**	**T/C**	**0.68**	**0.03(0.005)**	**6.39E-09**	**0.03(0.007)**	**1.22E-04**	**0.03**	**0.5**
**rs1851665**	**3/187919092**	**G/A**	**0.32**	**-0.03(0.005)**	**8.65E-09**	**-0.03(0.007)**	**1.92E-04**	**0.02**	**0.5**
**rs1836860**	**3/187919298**	**T/C**	**0.68**	**0.03(0.005)**	**7.30E-09**	**0.03(0.007)**	**2.02E-04**	**0.02**	**0.5**
**rs1403694**	**3/187922692**	**T/C**	**0.61**	**0.03(0.004)**	**1.50E-09**	**0.03(0.006)**	**2.02E-05**	**0.04**	**0.4**
**rs1648722**	**3/187931683**	**T/C**	**0.39**	**-0.03(0.004)**	**3.41E-12**	**-0.03(0.006)**	**3.13E-07**	**0.07**	**0.4**
**rs1656925**	**3/187931817**	**T/C**	**0.62**	**0.03(0.004)**	**1.29E-11**	**0.03(0.006)**	**4.77E-07**	**0.08**	**0.4**
**rs1648698**	**3/187932276**	**G/C**	**0.62**	**0.03(0.004)**	**1.25E-11**	**0.03(0.006)**	**4.40E-07**	**0.08**	**0.4**
**rs1648700**	**3/187932610**	**T/C**	**0.62**	**0.03(0.004)**	**1.75E-11**	**0.03(0.006)**	**3.85E-07**	**0.09**	**0.3**

**rs1624569**	**3/187932763**	**T/C**	**0.62**	**0.03(0.004)**	**1.31E-11**	**0.03(0.006)**	**4.75E-07**	**0.08**	**0.4**
**rs710448**	**3/187935579**	**G/A**	**0.38**	**-0.03(0.004)**	**2.17E-11**	**-0.03(0.006)**	**3.32E-06**	**0.04**	**0.4**
**rs5030060**	**3/187935611**	**T/C**	**0.31**	**0.03(0.005)**	**2.22E-09**	**0.03(0.006)**	**7.20E-06**	**0.07**	**0.4**
**rs822363**	**3/187935685**	**G/C**	**0.38**	**-0.03(0.005)**	**3.12E-11**	**-0.03(0.006)**	**2.12E-06**	**0.05**	**0.4**
rs5030062	3/187936874	C/A	0.38	0.03(0.005)	2.77E-12	0.04(0.008)	7.12E-06	1.0E-03	0.6
rs5030072	3/187938240	T/C	0.56	-0.03(0.005)	9.89E-11	-0.03(0.008)	2.50E-05	2.1E-03	0.6
rs3856930	3/187941016	T/C	0.35	0.03(0.005)	2.31E-10	0.03(0.007)	9.97E-06	0.01	0.5
rs698078	3/187941921	G/A	0.41	0.03(0.004)	7.19E-13	0.04(0.007)	1.95E-06	3.8E-03	0.6
rs710446	3/187942621	T/C	0.59	-0.03(0.004)	1.84E-12	-0.04(0.007)	5.09E-06	1.9E-03	0.6
**rs5030091**	**3/187943571**	**T/C**	**0.59**	**0.04(0.005)**	**9.67E-14**	**0.04(0.007)**	**2.99E-07**	**0.03**	**0.5**
**rs2062632**	**3/187943875**	**T/C**	**0.75**	**0.06(0.006)**	**2.52E-19**	**0.06(0.006)**	**8.78E-17**	**0.33**	**0.1**
**rs266760**	**3/187943910**	**G/A**	**0.72**	**0.05(0.007)**	**3.62E-13**	**0.05(0.007)**	**1.72E-12**	**0.40**	**0.0**
**rs1972703**	**3/187946037**	**G/A**	**0.84**	**0.06(0.007)**	**6.96E-16**	**0.06(0.008)**	**2.66E-13**	**0.29**	**0.1**
**rs822373**	**3/187953880**	**G/A**	**0.34**	**0.03(0.005)**	**1.77E-09**	**0.03(0.005)**	**1.77E-09**	**0.47**	**0.0**
**rs266749**	**3/187954613**	**T/C**	**0.36**	**0.03(0.005)**	**1.72E-10**	**0.03(0.005)**	**4.67E-09**	**0.31**	**0.1**
**rs266743**	**3/187958485**	**T/C**	**0.24**	**-0.05(0.006)**	**5.74E-14**	**-0.05(0.007)**	**2.07E-11**	**0.25**	**0.2**
**rs266742**	**3/187958539**	**T/G**	**0.65**	**-0.04(0.005)**	**2.93E-12**	**-0.04(0.005)**	**2.93E-12**	**0.59**	**0.0**
**rs822355**	**3/187962817**	**T/C**	**0.68**	**-0.05(0.006)**	**2.20E-16**	**-0.05(0.006)**	**2.20E-16**	**0.48**	**0.0**
**rs822354**	**3/187962900**	**G/A**	**0.68**	**-0.05(0.005)**	**1.53E-20**	**-0.05(0.005)**	**1.53E-20**	**0.61**	**0.0**
**rs266733**	**3/187976007**	**T/G**	**0.51**	**0.05(0.005)**	**3.99E-22**	**0.05(0.006)**	**8.31E-15**	**0.13**	**0.3**
**rs185554**	**3/187977116**	**G/A**	**0.52**	**0.05(0.005)**	**3.06E-27**	**0.05(0.006)**	**1.15E-18**	**0.16**	**0.3**
rs266719	3/187984342	T/C	0.22	0.07(0.005)	4.92E-38	0.08(0.009)	5.51E-16	2.1E-03	0.6
**rs3900626**	**3/187984594**	**T/C**	**0.17**	**-0.05(0.006)**	**5.71E-13**	**-0.05(0.007)**	**9.19E-11**	**0.30**	**0.1**
**rs1426810**	**3/187986129**	**G/A**	**0.36**	**0.05(0.005)**	**2.84E-30**	**0.06(0.007)**	**1.01E-14**	**0.01**	**0.5**
**rs1354091**	**3/187988594**	**T/G**	**0.74**	**0.05(0.005)**	**9.71E-20**	**0.05(0.005)**	**2.24E-18**	**0.39**	**0.1**
**rs2066500**	**3/187990616**	**T/C**	**0.26**	**-0.05(0.005)**	**9.72E-20**	**-0.05(0.005)**	**1.20E-18**	**0.40**	**0.0**
**rs266759**	**3/187991006**	**T/C**	**0.49**	**-0.05(0.004)**	**1.96E-29**	**-0.05(0.006)**	**1.63E-17**	**0.09**	**0.4**
rs266756	3/187991268	G/C	0.22	0.07(0.006)	2.21E-38	0.08(0.009)	8.47E-17	3.1E-03	0.6
**rs266754**	**3/187991660**	**T/C**	**0.48**	**-0.05(0.004)**	**2.32E-29**	**-0.05(0.006)**	**2.79E-17**	**0.08**	**0.4**
**rs187868**	**3/187992211**	**G/A**	**0.52**	**0.05(0.004)**	**1.21E-29**	**0.05(0.006)**	**2.16E-17**	**0.08**	**0.4**
**rs3917117**	**3/187998538**	**G/A**	**0.74**	**0.05(0.005)**	**3.53E-20**	**0.05(0.005)**	**3.53E-20**	**0.49**	**0.0**
**rs843991**	**3/187999122**	**T/C**	**0.49**	**0.05(0.004)**	**2.73E-30**	**0.05(0.005)**	**1.29E-20**	**0.17**	**0.3**
**rs3917110**	**3/188001346**	**G/C**	**0.26**	**-0.05(0.005)**	**1.47E-20**	**-0.05(0.005)**	**1.47E-20**	**0.48**	**0.0**
**rs3917109**	**3/188001500**	**C/A**	**0.73**	**0.05(0.006)**	**5.22E-15**	**0.05(0.006)**	**2.48E-14**	**0.41**	**0.0**
**rs16861184**	**3/188003171**	**T/C**	**0.26**	**-0.05(0.005)**	**9.62E-21**	**-0.05(0.005)**	**9.62E-21**	**0.51**	**0.0**
**rs710450**	**3/188005327**	**C/A**	**0.49**	**-0.05(0.005)**	**1.97E-19**	**-0.05(0.007)**	**1.59E-11**	**0.10**	**0.4**
**rs2293243**	**3/188005431**	**T/A**	**0.26**	**-0.05(0.005)**	**5.34E-21**	**-0.05(0.005)**	**5.34E-21**	**0.54**	**0.0**
rs266717	3/188013178	T/C	0.48	0.04(0.005)	1.62E-20	0.05(0.012)	1.38E-05	2.2E-12	0.8
**rs16861189**	**3/188018128**	**G/C**	**0.47**	**-0.03(0.005)**	**4.82E-08**	**-0.03(0.006)**	**2.57E-05**	**0.10**	**0.3**
**rs6810075**	**3/188031259**	**T/C**	**0.63**	**0.07(0.005)**	**3.56E-41**	**0.07(0.005)**	**3.56E-41**	**0.65**	**0.0**
**rs10937273**	**3/188032389**	**G/A**	**0.58**	**-0.04(0.005)**	**9.61E-18**	**-0.04(0.006)**	**9.03E-11**	**0.09**	**0.3**
**rs1648707**	**3/188034405**	**C/A**	**0.36**	**-0.06(0.005)**	**8.63E-40**	**-0.06(0.005)**	**8.63E-40**	**0.68**	**0.0**
**rs864265**	**3/188036986**	**T/G**	**0.14**	**-0.04(0.006)**	**1.43E-08**	**-0.04(0.008)**	**1.38E-05**	**0.10**	**0.3**

rs822387	3/188038731	T/C	0.92	-0.15(0.009)	7.43E-61	-0.15(0.014)	7.97E-24	3.6E-03	0.6
rs17300539	3/188042154	G/A	0.94	-0.18(0.01)	4.49E-77	-0.19(0.016)	4.69E-31	2.2E-03	0.6
**rs266729**	**3/188042168**	**G/C**	**0.27**	**-0.06(0.005)**	**3.33E-29**	**-0.06(0.005)**	**3.33E-29**	**0.80**	**0.0**
**rs182052**	**3/188043476**	**G/A**	**0.64**	**0.06(0.005)**	**6.65E-41**	**0.06(0.005)**	**6.65E-41**	**0.52**	**0.0**
rs16861209	3/188045808	C/A	0.93	-0.19(0.01)	3.27E-77	-0.2(0.018)	1.62E-28	5.8E-04	0.6
rs16861210	3/188049192	G/A	0.93	-0.18(0.01)	9.20E-73	-0.19(0.016)	2.45E-31	0.01	0.5
**rs12495941**	**3/188050874**	**T/G**	**0.33**	**0.04(0.006)**	**6.15E-12**	**0.04(0.006)**	**6.15E-12**	**0.47**	**0.0**
rs7649121	3/188051479	T/A	0.20	-0.07(0.007)	1.73E-18	-0.07(0.011)	2.08E-08	0.01	0.5
rs17366568	3/188053147	G/A	0.90	0.15(0.009)	2.75E-66	0.2(0.027)	7.64E-13	1.1E-13	0.9
rs1501299	3/188053817	T/G	0.27	0.07(0.006)	1.75E-31	0.07(0.01)	7.15E-11	2.0E-03	0.6
**rs3821799**	**3/188054180**	**T/C**	**0.45**	**0.03(0.005)**	**3.25E-10**	**0.03(0.006)**	**6.64E-07**	**0.12**	**0.3**
rs3774261	3/188054253	G/A	0.61	-0.07(0.005)	1.30E-49	-0.07(0.007)	2.64E-21	0.01	0.5
rs6773957	3/188056399	G/A	0.61	-0.07(0.005)	9.81E-50	-0.07(0.007)	2.60E-21	0.01	0.5
**rs1063538**	**3/188056877**	**T/C**	**0.38**	**0.06(0.005)**	**6.15E-30**	**0.06(0.008)**	**3.55E-13**	**0.03**	**0.5**
rs7639352	3/188061168	T/C	0.29	0.07(0.005)	2.21E-46	0.07(0.008)	4.29E-17	1.3E-03	0.6
rs6444175	3/188062438	G/A	0.71	-0.07(0.005)	6.85E-46	-0.07(0.008)	7.01E-17	1.3E-03	0.6
**rs7615090**	**3/188073697**	**T/G**	**0.88**	**0.06(0.008)**	**2.81E-11**	**0.07(0.013)**	**1.95E-07**	**0.06**	**0.4**
**rs2954018**	**8/126546335**	**T/C**	**0.70**	**0.03(0.005)**	**4.81E-08**	**0.03(0.005)**	**4.81E-08**	**0.65**	**0.0**
**rs2980880**	**8/126550154**	**G/A**	**0.31**	**-0.03(0.005)**	**1.11E-08**	**-0.03(0.005)**	**1.11E-08**	**0.55**	**0.0**
**rs2980879**	**8/126550657**	**T/A**	**0.69**	**0.03(0.005)**	**1.08E-08**	**0.03(0.005)**	**1.08E-08**	**0.55**	**0.0**
**rs2980878**	**8/126550709**	**G/C**	**0.30**	**-0.03(0.005)**	**1.56E-08**	**-0.03(0.005)**	**1.56E-08**	**0.60**	**0.0**
**rs2980876**	**8/126550876**	**T/C**	**0.69**	**0.03(0.005)**	**1.20E-08**	**0.03(0.005)**	**1.20E-08**	**0.58**	**0.0**
**rs2954026**	**8/126553708**	**T/G**	**0.30**	**-0.03(0.005)**	**2.16E-08**	**-0.03(0.005)**	**2.16E-08**	**0.61**	**0.0**
**rs7846466**	**8/126554713**	**T/C**	**0.30**	**-0.03(0.005)**	**2.41E-08**	**-0.03(0.005)**	**2.41E-08**	**0.61**	**0.0**
**rs2954032**	**8/126562574**	**G/A**	**0.70**	**0.03(0.005)**	**4.11E-08**	**0.03(0.005)**	**4.11E-08**	**0.61**	**0.0**
**rs2954033**	**8/126562928**	**G/A**	**0.70**	**0.03(0.005)**	**4.30E-08**	**0.03(0.005)**	**4.30E-08**	**0.60**	**0.0**
**rs2980859**	**8/126568611**	**G/C**	**0.69**	**0.03(0.005)**	**4.88E-08**	**0.03(0.005)**	**4.88E-08**	**0.61**	**0.0**
**rs2980888**	**8/126576490**	**T/C**	**0.29**	**-0.03(0.006)**	**3.92E-08**	**-0.03(0.006)**	**3.92E-08**	**0.82**	**0.0**
**rs2954038**	**8/126576571**	**C/A**	**0.29**	**-0.03(0.006)**	**4.82E-08**	**-0.03(0.006)**	**4.82E-08**	**0.78**	**0.0**
**rs11045172**	**12/20361488**	**C/A**	**0.20**	**0.04(0.006)**	**4.76E-08**	**0.04(0.008)**	**1.87E-06**	**0.23**	**0.2**
**rs12812995**	**12/20369141**	**G/C**	**0.63**	**-0.03(0.005)**	**3.10E-08**	**-0.03(0.005)**	**3.38E-07**	**0.33**	**0.1**
**rs1444636**	**12/20384150**	**G/C**	**0.63**	**-0.03(0.005)**	**3.39E-08**	**-0.03(0.005)**	**3.37E-07**	**0.33**	**0.1**
**rs10770643**	**12/20385101**	**G/A**	**0.38**	**0.03(0.005)**	**2.75E-08**	**0.03(0.005)**	**5.44E-07**	**0.29**	**0.1**
**rs7303397**	**12/20385638**	**G/A**	**0.38**	**0.03(0.005)**	**4.21E-08**	**0.03(0.005)**	**7.16E-07**	**0.30**	**0.1**
**rs7955516**	**12/20389303**	**C/A**	**0.38**	**0.03(0.005)**	**2.43E-08**	**0.03(0.005)**	**5.28E-07**	**0.29**	**0.2**
**rs2120757**	**12/20393267**	**T/C**	**0.64**	**-0.03(0.005)**	**3.97E-08**	**-0.03(0.006)**	**3.65E-06**	**0.18**	**0.2**
**rs3943606**	**12/20399304**	**T/C**	**0.36**	**0.03(0.005)**	**4.85E-08**	**0.03(0.006)**	**5.47E-06**	**0.16**	**0.3**
**rs2009084**	**12/20402460**	**T/C**	**0.61**	**-0.03(0.005)**	**4.56E-08**	**-0.03(0.006)**	**6.83E-06**	**0.15**	**0.3**
**rs11057405**	**12/121347850**	**G/A**	**0.90**	**0.05(0.009)**	**5.58E-09**	**0.05(0.01)**	**1.43E-06**	**0.26**	**0.2**
**rs2454722**	**12/121737171**	**G/A**	**0.18**	**0.04(0.006)**	**5.66E-11**	**0.04(0.006)**	**1.23E-10**	**0.43**	**0.0**
**rs601339**	**12/121740696**	**G/A**	**0.18**	**0.04(0.006)**	**3.88E-11**	**0.04(0.006)**	**5.20E-11**	**0.44**	**0.0**
**rs4759361**	**12/121744233**	**T/A**	**0.82**	**-0.04(0.006)**	**4.51E-11**	**-0.04(0.006)**	**1.48E-10**	**0.41**	**0.0**
**rs509548**	**12/121747808**	**T/A**	**0.82**	**-0.04(0.006)**	**4.69E-11**	**-0.04(0.006)**	**1.95E-10**	**0.40**	**0.0**

**rs6488898**	**12/122769785**	**G/A**	**0.08**	**-0.05(0.009)**	**1.48E-09**	**-0.05(0.011)**	**1.03E-05**	**0.10**	**0.3**
**rs11057354**	**12/122833591**	**G/A**	**0.88**	**0.04(0.007)**	**1.98E-09**	**0.04(0.008)**	**1.68E-07**	**0.25**	**0.2**
**rs12317176**	**12/122970671**	**T/C**	**0.66**	**-0.03(0.005)**	**2.52E-08**	**-0.03(0.005)**	**2.52E-08**	**0.61**	**0.0**
**rs7301953**	**12/122971824**	**G/A**	**0.68**	**-0.03(0.005)**	**1.83E-08**	**-0.03(0.005)**	**1.83E-08**	**0.73**	**0.0**
**rs10846579**	**12/122973356**	**T/C**	**0.34**	**0.03(0.005)**	**2.92E-08**	**0.03(0.005)**	**2.92E-08**	**0.61**	**0.0**
**rs11057394**	**12/122973629**	**T/C**	**0.67**	**-0.03(0.005)**	**7.32E-09**	**-0.03(0.005)**	**7.32E-09**	**0.77**	**0.0**
**rs12809125**	**12/122973944**	**G/A**	**0.34**	**0.03(0.005)**	**2.86E-08**	**0.03(0.005)**	**2.86E-08**	**0.61**	**0.0**
**rs7133378**	**12/122975455**	**G/A**	**0.69**	**-0.03(0.005)**	**1.29E-09**	**-0.03(0.005)**	**1.29E-09**	**0.65**	**0.0**
**rs9971695**	**12/122979444**	**G/A**	**0.67**	**-0.03(0.005)**	**2.47E-08**	**-0.03(0.005)**	**2.47E-08**	**0.62**	**0.0**
**rs3802999**	**12/122980051**	**T/C**	**0.33**	**0.03(0.005)**	**2.55E-08**	**0.03(0.005)**	**2.55E-08**	**0.60**	**0.0**
**rs4930721**	**12/122983842**	**T/C**	**0.31**	**0.03(0.005)**	**1.49E-08**	**0.03(0.005)**	**1.49E-08**	**0.71**	**0.0**
**rs12298484**	**12/122984627**	**T/C**	**0.33**	**0.03(0.005)**	**2.12E-08**	**0.03(0.005)**	**2.12E-08**	**0.61**	**0.0**
**rs11057396**	**12/122985015**	**C/A**	**0.32**	**0.03(0.005)**	**4.57E-08**	**0.03(0.005)**	**4.57E-08**	**0.90**	**0.0**
**rs11057397**	**12/122985681**	**T/C**	**0.33**	**0.03(0.005)**	**1.61E-08**	**0.03(0.005)**	**1.61E-08**	**0.59**	**0.0**
**rs9863**	**12/122987406**	**T/C**	**0.65**	**-0.03(0.005)**	**3.30E-08**	**-0.03(0.005)**	**3.30E-08**	**0.73**	**0.0**
**rs4930723**	**12/122989553**	**G/C**	**0.67**	**-0.03(0.005)**	**1.99E-08**	**-0.03(0.005)**	**1.99E-08**	**0.60**	**0.0**
**rs11057401**	**12/122993259**	**T/A**	**0.69**	**-0.03(0.005)**	**6.89E-09**	**-0.03(0.005)**	**6.89E-09**	**0.74**	**0.0**
**rs4930726**	**12/122994284**	**T/C**	**0.67**	**-0.03(0.005)**	**1.53E-08**	**-0.03(0.005)**	**1.53E-08**	**0.59**	**0.0**
**rs2178663**	**12/122999858**	**T/C**	**0.31**	**0.03(0.005)**	**1.02E-08**	**0.03(0.005)**	**1.02E-08**	**0.70**	**0.0**
**rs4405410**	**12/123001741**	**T/A**	**0.67**	**-0.03(0.005)**	**1.53E-08**	**-0.03(0.005)**	**1.53E-08**	**0.59**	**0.0**
**rs7961449**	**12/123003168**	**T/A**	**0.67**	**-0.03(0.005)**	**1.50E-08**	**-0.03(0.005)**	**1.50E-08**	**0.58**	**0.0**
**rs7964945**	**12/123003621**	**T/A**	**0.85**	**-0.04(0.006)**	**2.61E-08**	**-0.04(0.006)**	**2.61E-08**	**0.62**	**0.0**
**rs3867146**	**12/123003958**	**G/A**	**0.88**	**0.04(0.007)**	**2.47E-08**	**0.04(0.008)**	**1.33E-07**	**0.35**	**0.1**
**rs7132655**	**12/123004926**	**G/C**	**0.67**	**-0.03(0.005)**	**1.57E-08**	**-0.03(0.005)**	**1.57E-08**	**0.59**	**0.0**
**rs4765219**	**12/123006063**	**C/A**	**0.67**	**-0.03(0.005)**	**1.76E-08**	**-0.03(0.005)**	**1.76E-08**	**0.60**	**0.0**
**rs7958691**	**12/123006696**	**T/G**	**0.33**	**0.03(0.005)**	**1.67E-08**	**0.03(0.005)**	**1.67E-08**	**0.59**	**0.0**
**rs7305864**	**12/123007833**	**G/C**	**0.33**	**0.03(0.005)**	**6.58E-09**	**0.03(0.005)**	**6.58E-09**	**0.72**	**0.0**
**rs6488913**	**12/123011522**	**G/C**	**0.67**	**-0.03(0.005)**	**1.33E-08**	**-0.03(0.005)**	**1.33E-08**	**0.58**	**0.0**
**rs7312404**	**12/123012681**	**G/A**	**0.32**	**0.03(0.005)**	**1.78E-08**	**0.03(0.005)**	**1.78E-08**	**0.85**	**0.0**
**rs11831913**	**12/123012851**	**T/C**	**0.69**	**-0.03(0.005)**	**6.68E-09**	**-0.03(0.005)**	**6.68E-09**	**0.70**	**0.0**
**rs6488914**	**12/123013794**	**G/C**	**0.69**	**-0.03(0.005)**	**1.53E-08**	**-0.03(0.005)**	**1.53E-08**	**0.71**	**0.0**
**rs7973683**	**12/123015176**	**C/A**	**0.67**	**-0.03(0.005)**	**8.44E-09**	**-0.03(0.005)**	**8.44E-09**	**0.54**	**0.0**
**rs4765127**	**12/123026120**	**T/G**	**0.33**	**0.03(0.005)**	**4.63E-09**	**0.03(0.005)**	**4.63E-09**	**0.66**	**0.0**
**rs12311114**	**12/123026656**	**C/A**	**0.69**	**-0.03(0.005)**	**1.06E-08**	**-0.03(0.005)**	**1.06E-08**	**0.60**	**0.0**
**rs4765528**	**12/123028207**	**T/A**	**0.69**	**-0.03(0.005)**	**4.56E-09**	**-0.03(0.005)**	**4.56E-09**	**0.71**	**0.0**
**rs11057408**	**12/123030789**	**T/G**	**0.33**	**0.03(0.005)**	**1.09E-08**	**0.03(0.005)**	**1.09E-08**	**0.65**	**0.0**
**rs7978610**	**12/123034525**	**G/C**	**0.67**	**-0.03(0.005)**	**1.61E-09**	**-0.03(0.005)**	**1.61E-09**	**0.86**	**0.0**
**rs952632**	**12/123035691**	**G/A**	**0.33**	**0.03(0.005)**	**4.13E-09**	**0.03(0.005)**	**4.13E-09**	**0.69**	**0.0**
**rs11837287**	**12/123036012**	**T/C**	**0.33**	**0.03(0.005)**	**5.79E-09**	**0.03(0.005)**	**5.79E-09**	**0.71**	**0.0**
**rs7311969**	**12/123036286**	**T/C**	**0.67**	**-0.03(0.005)**	**4.43E-09**	**-0.03(0.005)**	**4.43E-09**	**0.63**	**0.0**
**rs7307277**	**12/123041109**	**G/A**	**0.33**	**0.03(0.005)**	**5.81E-09**	**0.03(0.005)**	**5.81E-09**	**0.69**	**0.0**
**rs7311233**	**12/123041893**	**G/A**	**0.31**	**0.03(0.005)**	**4.24E-09**	**0.03(0.005)**	**4.24E-09**	**0.69**	**0.0**
**rs12809473**	**12/123042552**	**T/A**	**0.67**	**-0.03(0.005)**	**5.98E-09**	**-0.03(0.005)**	**5.98E-09**	**0.70**	**0.0**

rs12827409	12/123042659	G/C	0.33	0.03(0.005)	6.05E-09	0.03(0.005)	6.05E-09	0.70	0.0
rs7135314	12/123044519	T/A	0.33	0.03(0.005)	6.22E-09	0.03(0.005)	6.22E-09	0.69	0.0
rs4765148	12/123044590	T/G	0.31	0.03(0.005)	6.63E-09	0.03(0.005)	6.63E-09	0.62	0.0
rs11057409	12/123045284	T/C	0.33	0.03(0.005)	5.59E-09	0.03(0.005)	5.59E-09	0.70	0.0
rs7975482	12/123047643	G/A	0.33	0.03(0.005)	7.68E-09	0.03(0.005)	7.68E-09	0.68	0.0
rs2130382	12/123050596	G/C	0.31	0.03(0.005)	8.27E-09	0.03(0.005)	8.27E-09	0.71	0.0
rs12310367	12/123052631	G/A	0.33	0.03(0.005)	1.16E-08	0.03(0.005)	1.16E-08	0.63	0.0
rs11057412	12/123055095	G/C	0.67	-0.03(0.005)	1.24E-08	-0.03(0.005)	1.24E-08	0.62	0.0
rs1187415	12/123057482	G/C	0.33	0.03(0.005)	9.62E-09	0.03(0.005)	9.62E-09	0.55	0.0
rs12303671	12/123058563	T/G	0.67	-0.03(0.005)	1.30E-08	-0.03(0.005)	1.30E-08	0.61	0.0
rs7307053	12/123060493	T/C	0.31	0.03(0.005)	1.64E-08	0.03(0.005)	1.64E-08	0.69	0.0
rs12824567	12/123061156	G/C	0.69	-0.03(0.005)	1.54E-08	-0.03(0.005)	1.54E-08	0.69	0.0
rs863750	12/123071397	T/C	0.61	-0.03(0.005)	6.41E-09	-0.03(0.005)	6.41E-09	0.76	0.0
rs10773049	12/123072584	T/C	0.62	-0.03(0.005)	2.67E-08	-0.03(0.005)	2.67E-08	0.74	0.0
rs825453	12/123074711	T/A	0.62	-0.03(0.005)	4.03E-08	-0.03(0.005)	4.03E-08	0.77	0.0
rs11057418	12/123074929	G/C	0.76	0.03(0.006)	3.14E-08	0.04(0.006)	1.06E-07	0.38	0.1
rs2927328	16/80067223	T/C	0.36	-0.03(0.005)	1.69E-10	-0.04(0.007)	9.48E-07	0.03	0.4
rs1471379	16/80068200	T/C	0.38	-0.03(0.005)	3.20E-10	-0.03(0.006)	7.32E-08	0.15	0.3
rs1966957	16/80068580	G/C	0.38	-0.03(0.004)	2.66E-10	-0.03(0.006)	8.42E-08	0.13	0.3
rs2927327	16/80069035	T/A	0.38	-0.03(0.004)	2.47E-10	-0.03(0.006)	7.77E-08	0.13	0.3
rs2966079	16/80069307	T/C	0.55	0.03(0.005)	1.56E-11	0.04(0.007)	5.49E-07	0.01	0.5
rs1471152	16/80070137	T/G	0.38	-0.03(0.004)	3.38E-10	-0.03(0.006)	8.23E-08	0.15	0.3
rs2927324	16/80070322	T/C	0.55	0.03(0.005)	1.29E-11	0.04(0.007)	4.89E-07	0.01	0.5
rs2927323	16/80070449	G/A	0.32	-0.03(0.005)	4.77E-12	-0.04(0.007)	5.18E-08	0.06	0.4
rs2927322	16/80072006	G/A	0.70	0.04(0.005)	4.86E-13	0.04(0.007)	1.02E-08	0.07	0.4
rs2966085	16/80074135	G/A	0.64	0.03(0.005)	5.70E-12	0.04(0.006)	1.21E-08	0.12	0.3
rs2317241	16/80077267	G/A	0.70	0.03(0.005)	5.45E-11	0.04(0.006)	7.32E-09	0.22	0.2
rs12443634	16/80081775	C/A	0.72	0.04(0.005)	1.59E-16	0.04(0.005)	1.57E-15	0.40	0.1
rs2925979	16/80092291	T/C	0.30	-0.04(0.005)	1.87E-18	-0.04(0.005)	1.87E-18	0.52	0.0
rs2966093	16/80096121	G/A	0.36	-0.04(0.005)	1.36E-16	-0.04(0.006)	1.59E-10	0.09	0.4
rs2966094	16/80096138	C/A	0.65	0.04(0.005)	5.53E-17	0.04(0.006)	5.90E-11	0.09	0.3
rs2927307	16/80101333	G/A	0.42	-0.04(0.004)	4.16E-17	-0.04(0.006)	1.05E-09	0.04	0.4
rs2966095	16/80106959	G/A	0.64	0.04(0.005)	1.94E-16	0.04(0.006)	5.81E-11	0.12	0.3
rs2966097	16/80107209	T/C	0.64	0.04(0.005)	1.48E-14	0.04(0.007)	7.19E-09	0.08	0.4
rs11865200	16/81183206	G/A	0.16	-0.04(0.006)	1.14E-09	-0.04(0.009)	3.84E-05	0.03	0.4
rs7196910	16/81183882	G/C	0.28	-0.03(0.005)	1.63E-08	-0.03(0.008)	1.58E-04	0.01	0.5
rs8058318	16/81185746	G/A	0.35	0.03(0.005)	3.26E-08	0.03(0.005)	8.05E-07	0.27	0.2
rs8047615	16/81191554	G/A	0.75	0.03(0.005)	2.34E-08	0.03(0.008)	1.71E-04	0.01	0.5
rs8062637	16/81192869	T/C	0.22	-0.04(0.005)	6.08E-10	-0.04(0.009)	2.29E-04	9.8E-04	0.6
rs7200895	16/81202107	T/C	0.83	0.04(0.006)	1.85E-12	0.05(0.015)	5.52E-04	8.7E-10	0.8
rs3852724	16/81203595	C/A	0.82	0.04(0.006)	9.62E-10	0.05(0.013)	8.07E-04	1.2E-08	0.8
rs3865185	16/81203963	T/A	0.18	-0.04(0.006)	7.08E-10	-0.05(0.013)	7.03E-04	1.1E-08	0.8
rs3865186	16/81204473	G/A	0.20	-0.03(0.005)	9.53E-09	-0.04(0.011)	6.58E-04	1.1E-06	0.7

**rs8060301**	**16/81219245**	**T/A**	**0.54**	**0.03(0.004)**	**3.28E-08**	**0.03(0.006)**	**1.48E-05**	**0.07**	**0.4**
**rs4783244**	**16/81219769**	**T/G**	**0.46**	**-0.03(0.004)**	**3.70E-08**	**-0.03(0.006)**	**3.17E-05**	**0.05**	**0.4**
**rs12051272**	**16/81220789**	**T/G**	**0.03**	**-0.28(0.018)**	**1.41E-49**	**-0.3(0.03)**	**1.48E-21**	**0.05**	**0.5**
rs8047711	16/81225172	G/A	0.97	0.18(0.015)	4.10E-29	0.18(0.031)	1.55E-08	8.8E-05	0.7
rs16957913	16/81227750	T/C	0.97	0.16(0.015)	8.13E-25	0.17(0.033)	1.21E-06	4.0E-06	0.8
rs12599599	16/81228040	G/A	0.97	0.16(0.015)	3.90E-25	0.17(0.033)	6.96E-07	7.7E-06	0.7
**rs12922394**	**16/81229828**	**T/C**	**0.07**	**-0.1(0.011)**	**3.16E-18**	**-0.1(0.015)**	**1.31E-11**	**0.14**	**0.3**
**rs1870843**	**16/81316815**	**G/A**	**0.36**	**0.03(0.005)**	**4.26E-11**	**0.03(0.005)**	**3.49E-09**	**0.26**	**0.2**
**rs11150491**	**16/81318003**	**T/G**	**0.63**	**-0.03(0.005)**	**2.20E-10**	**-0.03(0.005)**	**2.20E-10**	**0.50**	**0.0**
**rs889140**	**19/38580840**	**G/A**	**0.35**	**-0.03(0.005)**	**9.81E-10**	**-0.03(0.005)**	**1.58E-08**	**0.30**	**0.1**
**rs889139**	**19/38581209**	**G/A**	**0.65**	**0.03(0.005)**	**1.09E-09**	**0.03(0.005)**	**1.53E-08**	**0.30**	**0.1**
**rs731839**	**19/38590905**	**G/A**	**0.35**	**-0.04(0.005)**	**2.20E-13**	**-0.04(0.005)**	**6.43E-13**	**0.42**	**0.0**
**rs4805885**	**19/38597963**	**T/C**	**0.39**	**-0.03(0.005)**	**1.25E-11**	**-0.03(0.005)**	**1.25E-11**	**0.47**	**0.0**
**rs8182584**	**19/38601550**	**T/G**	**0.38**	**-0.03(0.005)**	**6.64E-11**	**-0.03(0.005)**	**6.64E-11**	**0.55**	**0.0**

*SNP with I2 less than 0.5 are listed in bold.
EA: Effect Allele
NEA: Non-Effect Allele
