# Supplementary material for: Novel Loci for Adiponectin Levels and Their Influence on Type 2 Diabetes and Metabolic Traits: A Multi-Ethnic Meta-Analysis of 45,891 Individuals
Source: PLoS Genet. 2012 Mar 29;8(3):e1002607. doi: 10.1371/journal.pgen.1002607 (PMC3315470; doi:10.1371/journal.pgen.1002607)
Supplement: Table S3 — Association Results of SNPs achieving p≤5×10^−6^ in the Discovery phase in European Populations (Sex-Combined Analysis). *Denotes SNPs typed in the de-novo follow-up phase. (PDF) [file pgen.1002607.s006.pdf]

Table S3: **Association Results of SNPs achieving $p \leq 5x10^{-6}$ in the Discovery phase in European Populations (Sex-Combined Analysis)**

SNP	Chr/Pos	EA/NEA	Discovery				Insilico and Denovo Follow-Up				Joint Analysis			
			Beta	Pvalue	I2	n	Beta	Pvalue	I2	n	Beta	Pvalue	I2	n
rs12088212	1/24454748	G/A	0.034	3.6E-06	0.0	29236	0.02	3.0E-01	0.0	5921	0.033	2.3E-06	0.0	35157
rs6424169	1/24463278	G/A	0.034	1.6E-06	0.0	29225	0.011	5.5E-01	0.0	5939	0.031	2.2E-06	0.0	35164
rs6700959	1/24463356	G/C	-0.034	2.2E-06	0.0	29220	-0.016	3.5E-01	0.0	6622	-0.031	1.9E-06	0.0	35842
rs10794657	1/24498760	G/A	0.029	1.7E-06	0.1	29227	0.032	3.1E-02	0.0	6622	0.029	1.5E-07	0.0	35849
rs621535	1/24512900	G/A	0.027	5.0E-06	0.1	29345	0.031	1.4E-01	0.0	2268	0.027	1.5E-06	0.0	31613
rs622345	1/24513044	T/C	-0.027	4.9E-06	0.1	29338	-0.037	1.5E-01	0.0	1585	-0.028	1.7E-06	0.0	30923
rs2791547	1/217726288	T/A	-0.021	4.7E-06	0.0	29328	-0.019	1.2E-01	0.0	5939	-0.021	1.2E-06	0.0	35267
rs2066152	1/217730656	G/A	-0.021	4.8E-06	0.0	29329	-0.034	3.2E-02	0.6	2268	-0.022	5.8E-07	0.0	31597
rs2791544	1/217731024	G/A	-0.021	3.3E-06	0.0	29329	-0.029	1.1E-02	0.0	6622	-0.022	1.4E-07	0.0	35951
rs2061155	1/217731632	T/C	-0.021	2.9E-06	0.0	29343	-0.029	1.1E-02	0.0	6622	-0.022	1.2E-07	0.0	35965
rs765751	1/217735856	T/C	0.021	3.3E-06	0.0	29325	0.019	1.2E-01	0.0	5927	0.021	8.9E-07	0.0	35252
rs12143910	1/217737616	T/A	-0.022	3.0E-06	0.4	29261	-0.026	2.3E-02	0.0	6622	-0.023	2.1E-07	0.2	35883
rs11118319	1/217737824	T/C	-0.022	3.1E-06	0.4	29261	-0.026	2.2E-02	0.0	6622	-0.023	2.1E-07	0.2	35883
rs2820468	1/217740320	G/A	-0.023	3.5E-06	0.3	29282	-0.033	5.3E-03	0.0	6622	-0.024	9.0E-08	0.1	35904
rs2820465	1/217741824	T/G	-0.022	1.4E-06	0.2	29294	-0.025	2.6E-02	0.0	6622	-0.023	1.1E-07	0.1	35916
rs2791553	1/217742672	G/A	-0.022	1.4E-06	0.2	29325	-0.016	1.9E-01	0.0	5939	-0.022	5.6E-07	0.0	35264
rs4323719	1/217744160	T/C	-0.021	2.9E-06	0.4	29344	-0.026	2.2E-02	0.0	6622	-0.022	2.0E-07	0.3	35966
rs6672377	1/217745360	C/A	-0.021	3.3E-06	0.4	29344	-0.026	2.2E-02	0.0	6622	-0.022	2.2E-07	0.3	35966
rs12044156	1/217746592	G/C	0.021	3.0E-06	0.4	29344	0.026	2.2E-02	0.0	6622	0.022	2.0E-07	0.3	35966
rs12031785	1/217753056	T/C	-0.021	4.3E-06	0.4	29344	-0.026	2.3E-02	0.0	6619	-0.022	3.0E-07	0.3	35963
rs2494195	1/217755264	T/C	0.021	3.0E-06	0.1	29327	0.015	2.2E-01	0.0	5939	0.021	1.4E-06	0.0	35266
rs1572505	1/217775392	C/A	-0.022	3.6E-06	0.2	29341	-0.03	1.0E-02	0.0	6622	-0.023	1.5E-07	0.0	35963
rs1337098	1/217776816	G/A	0.022	4.2E-06	0.2	29336	0.031	7.7E-03	0.0	6622	0.023	1.4E-07	0.0	35958
rs10779358	1/217780352	T/A	0.022	4.5E-06	0.2	29336	0.026	4.3E-02	0.0	5939	0.022	5.3E-07	0.0	35275
rs2820449	1/217781616	G/A	-0.022	4.7E-06	0.2	29336	-0.031	9.2E-03	0.0	6616	-0.023	1.8E-07	0.0	35952
rs1337101	1/217792720	T/G	0.023	2.0E-06	0.0	29321	0.035	3.8E-03	0.0	6622	0.025	4.0E-08	0.0	35943
rs3001032	1/217794400	T/C	-0.023	2.0E-06	0.0	29321	-0.035	3.3E-03	0.0	6609	-0.025	3.6E-08	0.0	35930
rs1415293	1/217796624	T/A	0.023	2.0E-06	0.0	29321	0.035	3.6E-03	0.0	6622	0.025	3.8E-08	0.0	35943
rs4846302	1/217797216	G/C	-0.023	1.8E-06	0.1	29329	-0.034	4.4E-03	0.0	6622	-0.025	4.0E-08	0.0	35951
rs2820441	1/217801584	C/A	0.023	1.8E-06	0.1	29335	0.027	3.8E-02	0.0	5939	0.024	1.9E-07	0.0	35274
rs1415288	1/217809168	C/A	0.024	2.6E-06	0.0	29303	0.033	8.6E-03	0.0	6622	0.025	8.9E-08	0.0	35925
rs2820446	1/217815440	G/C	0.023	4.1E-06	0.0	29327	0.028	3.8E-02	0.0	5939	0.023	4.4E-07	0.0	35266
rs4846567	1/217817344	T/G	0.023	4.3E-06	0.0	29333	0.033	7.1E-03	0.0	6622	0.024	1.3E-07	0.0	35955
rs2820443	1/217820128	T/C	-0.023	4.1E-06	0.0	29334	-0.032	8.4E-03	0.0	6622	-0.024	1.4E-07	0.0	35956
rs2943653	2/226756016	T/C	-0.022	3.9E-06	0.0	29300	-0.012	3.5E-01	0.0	5941	-0.021	2.9E-06	0.0	35241
rs952227	2/226770320	G/A	-0.022	4.5E-06	0.0	29279	-0.015	1.8E-01	0.0	7362	-0.021	1.9E-06	0.0	36641
rs2943646	2/226807776	G/A	-0.021	4.5E-06	0.2	29320	-0.019	1.1E-01	0.0	6679	-0.021	1.1E-06	0.1	35999
rs2972147	2/226808096	T/C	0.021	4.3E-06	0.2	29322	0.016	1.9E-01	0.0	5941	0.021	1.7E-06	0.1	35263
rs2972146	2/226808944	T/G	-0.021	4.6E-06	0.3	29312	-0.02	7.5E-02	0.0	6624	-0.021	8.3E-07	0.1	35936
rs2138157	2/226811968	C/A	-0.022	2.3E-06	0.3	29300	-0.019	1.3E-01	0.0	5941	-0.022	6.6E-07	0.1	35241
rs2943652	2/226816688	T/C	-0.022	3.1E-06	0.2	29311	-0.023	3.6E-02	0.0	7362	-0.022	3.1E-07	0.1	36673
rs2943656	2/226830160	G/A	-0.022	2.0E-06	0.3	29306	-0.017	1.5E-01	0.0	6679	-0.021	7.0E-07	0.1	35985
rs1515110	2/226830464	T/G	-0.022	1.6E-06	0.3	29307	-0.019	5.0E-02	0.0	9771	-0.022	2.1E-07	0.1	39078
rs1515108	2/226831328	T/C	-0.022	1.5E-06	0.3	29307	-0.016	1.6E-01	0.0	6679	-0.021	5.7E-07	0.1	35986
rs2943657	2/226831680	T/C	-0.022	1.5E-06	0.3	29307	-0.015	1.8E-01	0.0	6678	-0.021	6.2E-07	0.1	35985
rs2943659	2/226833504	G/A	0.022	2.6E-06	0.3	29307	0.02	8.3E-02	0.0	6475	0.022	5.1E-07	0.2	35782
rs1399626	2/226833664	T/A	0.022	2.3E-06	0.3	29308	0.016	1.6E-01	0.0	6679	0.021	8.0E-07	0.2	35987
rs2943660	2/226836784	T/G	0.022	3.5E-06	0.3	29307	0.017	1.3E-01	0.0	6624	0.021	1.1E-06	0.2	35931
rs1515099	2/226837136	G/A	0.022	3.4E-06	0.3	29308	0.012	3.1E-01	0.0	5941	0.021	2.3E-06	0.2	35249
rs1515100	2/226837168	C/A	0.021	5.0E-06	0.2	29307	0.042	2.2E-02	0.0	1587	0.023	5.8E-07	0.2	30894
rs2673135	2/226859840	G/A	0.022	3.6E-06	0.2	29307	0.02	6.2E-02	0.0	7362	0.021	5.5E-07	0.1	36669
rs2673142	2/226864896	G/C	0.022	3.4E-06	0.3	29280	0.015	1.8E-01	0.0	6679	0.021	1.4E-06	0.1	35959
rs2713548	2/226864912	T/C	-0.022	3.6E-06	0.2	29307	-0.02	6.0E-02	0.0	7362	-0.022	5.4E-07	0.1	36669
rs2458993	2/226864976	T/C	-0.022	3.9E-06	0.2	29285	-0.02	6.5E-02	0.0	7362	-0.022	6.3E-07	0.1	36647
rs2713549	2/226865456	G/A	-0.022	3.6E-06	0.2	29307	-0.018	1.1E-01	0.0	6624	-0.021	9.0E-07	0.1	35931
rs2713559	2/226872752	G/C	-0.022	4.2E-06	0.2	29307	-0.018	1.1E-01	0.0	6624	-0.021	1.0E-06	0.1	35931
rs2713557	2/226879840	T/C	0.022	4.3E-06	0.2	29307	0.018	1.0E-01	0.0	6624	0.021	1.0E-06	0.1	35931
rs2713552	2/226889920	T/G	-0.022	4.4E-06	0.2	29314	-0.017	1.5E-01	0.0	6624	-0.021	1.5E-06	0.1	35938
rs6442038	3/46935744	G/A	-0.023	3.7E-06	0.1	29289	-0.013	2.6E-01	0.0	7362	-0.021	2.5E-06	0.0	36651
rs6780601	3/46940860	G/A	-0.023	3.4E-06	0.1	29289	-0.014	2.2E-01	0.0	7362	-0.022	1.9E-06	0.0	36651
rs9852082	3/46947380	T/C	0.023	2.8E-06	0.1	29287	0.01	3.0E-01	0.0	10523	0.021	2.8E-06	0.0	39810
rs4683297	3/46948676	T/C	-0.023	1.9E-06	0.1	29296	-0.014	2.1E-01	0.0	7362	-0.022	1.0E-06	0.0	36658
rs4683296	3/46948824	G/C	-0.023	2.0E-06	0.1	29289	-0.014	1.9E-01	0.0	7362	-0.022	9.9E-07	0.0	36651
rs4683294	3/46954016	G/C	0.023	2.6E-06	0.1	29256	0.008	4.8E-01	0.0	6674	0.021	3.4E-06	0.1	35930
rs614288	3/52195244	T/C	-0.022	3.7E-06	0.3	29276	-0.026	2.6E-02	0.4	6624	-0.022	2.9E-07	0.3	35900
rs352162	3/52228008	T/C	-0.022	1.1E-06	0.5	29255	-0.031	5.5E-03	0.3	6624	-0.023	2.7E-08	0.4	35879
rs187084	3/52236072	G/A	0.022	2.7E-06	0.4	29220	-0.014	2.1E-01	0.5	6624	0.017	8.4E-05	0.5	35844
rs6445358	3/52283884	G/C	0.021	4.8E-06	0.4	29333	0.027	2.5E-02	0.0	5941	0.022	3.9E-07	0.3	35274
rs7622851	3/52308712	G/C	0.021	3.8E-06	0.4	29340	0.035	1.8E-03	0.0	6624	0.023	5.0E-08	0.3	35964
rs9311474	3/52313892	T/C	-0.021	3.8E-06	0.4	29343	-0.035	1.5E-03	0.0	6624	-0.023	4.2E-08	0.3	35967
rs648514	3/52442304	G/A	0.024	2.1E-07	0.4	29301	0.037	1.0E-03	0.1	6611	0.026	1.5E-09	0.3	35912
rs10865972	3/52466488	C/A	0.046	2.6E-06	0.0	29340	-0.023	3.5E-01	0.6	5940	0.037	4.2E-05	0.3	35280
rs1541495	3/52467748	T/C	0.047	1.8E-06	0.0	29340	-0.031	1.8E-01	0.5	6623	0.036	7.6E-05	0.3	35963
rs2109558	3/52476656	G/C	0.048	3.1E-06	0.0	27704	-0.031	2.4E-01	0.6	5941	0.038	7.0E-05	0.4	33645
rs6784615	3/52481464	T/C	-0.045	3.1E-06	0.0	29276	0.034	2.1E-01	0.6	5941	-0.036	6.1E-05	0.4	35217
rs11721286	3/52484068	G/A	-0.046	2.1E-06	0.0	29345	0.036	1.4E-01	0.5	6624	-0.035	8.6E-05	0.4	35969
rs4687617	3/52484316	G/A	0.046	2.7E-06	0.0	29344	-0.034	1.9E-01	0.7	4554	0.036	6.5E-05	0.4	33898
rs2109559	3/52487360	G/A	-0.046	2.1E-06	0.0	29345	0.036	1.4E-01	0.5	6624	-0.036	8.4E-05	0.4	35969
rs887514	3/52497700	T/C	0.049	1.1E-06	0.0	29345	-0.037	1.3E-01	0.5	6624	0.037	5.9E-05	0.4	35969
rs9863753	3/52504940	T/A	-0.05	8.0E-07	0.0	29324	0.038	1.3E-01	0.5	6624	-0.038	4.8E-05	0.3	35948
rs1010553	3/52515812	T/C	0.023	7.4E-07	0.4	29265	0.034	3.0E-03	0.0	6564	0.024	1.2E-08	0.2	35829
rs1010554	3/52517960	T/C	0.024	1.1E-07	0.4	29340	0.031	5.6E-03	0.0	6624	0.025	2.5E-09	0.2	35964
rs13081028	3/52530356	G/A	-0.028	1.1E-09	0.4	29308	-0.029	9.4E-03	0.0	6623	-0.028	3.0E-11	0.2	35931
rs9853056	3/52530996	T/C	-0.028	9.4E-10	0.4	29306	-0.032	5.1E-03	0.0	6475	-0.029	1.6E-11	0.2	35781
rs13303	3/52533048	T/C	-0.029	1.2E-07	0.3	20629	-0.029	1.0E-02	0.0	6623	-0.029	3.9E-09	0.0	27252

rs4282054	3/52541104	T/C	-0.029	6.3E-10	0.4	29306	-0.029	8.8E-03	0.0	6624	-0.029	1.7E-11	0.1	35930
rs7614981	3/52541952	C/A	-0.025	5.8E-08	0.4	29307	-0.023	5.3E-02	0.0	5940	-0.025	7.6E-09	0.2	35247
rs12489828	3/52542056	T/G	0.025	5.5E-08	0.4	29307	0.03	7.3E-03	0.0	6624	0.026	1.4E-09	0.2	35931
rs2590838	3/52597128	G/A	-0.03	7.1E-11	0.3	29340	-0.035	1.4E-03	0.0	6624	-0.03	3.9E-13	0.2	35964
rs2276824	3/52612528	G/C	0.03	3.1E-10	0.3	28950	0.03	8.9E-03	0.0	6624	0.03	8.3E-12	0.1	35574
rs9879090	3/52623304	T/C	-0.031	2.7E-10	0.3	28950	-0.03	8.8E-03	0.0	6624	-0.031	7.2E-12	0.1	35574
rs13083798	3/52624788	G/A	0.03	5.2E-11	0.3	29340	0.032	7.3E-03	0.0	5941	0.03	1.2E-12	0.2	35281
rs1108842	3/52695120	C/A	0.03	3.7E-11	0.3	29338	0.036	8.6E-04	0.0	6624	0.031	1.4E-13	0.2	35962
rs6617	3/52715224	G/C	0.021	4.5E-06	0.0	29254	-0.013	2.3E-01	0.0	6624	0.016	1.1E-04	0.3	35878
rs11235	3/52720128	T/C	0.031	2.8E-10	0.3	28952	0.031	8.2E-03	0.0	6624	0.031	7.1E-12	0.1	35576
rs2710323	3/52790944	T/C	-0.029	2.9E-10	0.3	29346	-0.002	8.7E-01	0.6	6624	-0.025	2.8E-09	0.5	35970
rs3617	3/52808844	C/A	-0.026	2.2E-08	0.1	29303	-0.035	1.6E-03	0.0	6611	-0.027	1.8E-10	0.0	35914
rs4481150	3/52812832	T/C	-0.025	3.3E-08	0.3	29328	-0.037	9.3E-04	0.0	6624	-0.027	1.9E-10	0.1	35952
rs2535627	3/52820144	T/C	-0.026	2.5E-08	0.3	29343	-0.038	5.6E-04	0.0	6624	-0.027	1.0E-10	0.1	35967
rs2071044	3/52822640	T/C	0.025	3.7E-08	0.3	29292	0.038	6.0E-04	0.0	6624	0.027	1.6E-10	0.1	35916
rs9831977	3/52829180	T/G	-0.033	4.3E-06	0.0	29319	0.011	5.2E-01	0.5	6624	-0.027	5.5E-05	0.1	35943
rs13076033	3/52873468	T/C	-0.037	7.1E-07	0.0	29344	0.015	3.9E-01	0.4	6624	-0.03	1.7E-05	0.0	35968
rs2952830	3/52957336	G/A	0.024	4.6E-06	0.3	29290	-0.002	8.7E-01	0.1	6624	0.02	2.7E-05	0.3	35914
rs2581777	3/53029768	T/C	0.023	1.6E-06	0.1	29343	-0.007	5.3E-01	0.2	6624	0.019	2.1E-05	0.3	35967
rs2244461	3/53030880	G/A	0.023	2.0E-06	0.1	29340	-0.007	5.5E-01	0.2	6624	0.019	2.4E-05	0.3	35964
rs2581806	3/53038400	G/A	-0.022	2.7E-06	0.1	29343	0.007	5.8E-01	0.2	6624	-0.018	2.9E-05	0.2	35967
rs2581818	3/53046692	C/A	0.022	3.4E-06	0.1	29312	-0.008	5.1E-01	0.1	6624	0.018	4.0E-05	0.3	35936
rs13072457	3/53077428	G/A	-0.033	1.0E-06	0.0	28796	0.019	2.4E-01	0.4	6624	-0.026	3.8E-05	0.3	35420
rs4301033	3/151525312	G/A	0.042	5.1E-08	0.0	29303	-0.012	5.8E-01	0.4	5792	0.036	6.4E-07	0.0	35095
rs7636643	3/151532464	T/A	-0.043	2.8E-08	0.0	29336	0.026	2.2E-01	0.5	5941	-0.036	1.2E-06	0.1	35277
rs16862607	3/151533984	G/A	-0.043	2.8E-08	0.0	29339	0.019	3.2E-01	0.4	6623	-0.035	1.3E-06	0.1	35962
rs16862610	3/151535504	T/C	0.044	2.6E-08	0.0	29340	-0.024	2.6E-01	0.5	5792	0.036	1.0E-06	0.1	35132
rs2060662	3/151536640	G/C	-0.044	2.5E-08	0.0	29341	0.021	2.8E-01	0.4	6623	-0.035	1.4E-06	0.1	35964
rs1597466	3/151538256	T/G	-0.044	1.9E-08	0.0	29319	0.023	2.2E-01	0.3	6475	-0.034	1.6E-06	0.1	35794
rs7616723	3/151538336	G/A	0.044	2.6E-08	0.0	29342	-0.023	2.3E-01	0.4	6624	0.035	1.8E-06	0.1	35966
rs7617025	3/151538656	G/A	0.044	2.3E-08	0.0	29342	-0.024	2.2E-01	0.4	6624	0.035	1.6E-06	0.1	35966
rs9844972	3/151580320	G/C	0.047	3.3E-07	0.0	26422	-0.045	3.4E-02	0.0	6624	0.033	9.0E-05	0.3	33046
rs9870756	3/187827312	T/C	-0.033	3.9E-06	0.0	29213	-0.036	3.6E-02	0.1	6624	-0.034	3.9E-07	0.0	35837
rs7646517	3/187902864	T/C	-0.044	4.5E-10	0.0	29347	0.02	2.5E-01	0.7	6624	-0.036	5.4E-08	0.6	35971
rs11927941	3/187915744	G/A	-0.032	4.0E-10	0.3	29036	0.013	3.0E-01	0.2	6475	-0.026	4.7E-08	0.5	35511
rs10937266	3/187915776	G/A	0.03	1.2E-09	0.3	29170	-0.012	3.0E-01	0.2	6624	0.024	1.2E-07	0.5	35794
rs11924390	3/187915792	T/C	0.03	1.2E-09	0.3	29171	-0.013	3.0E-01	0.2	6624	0.024	1.2E-07	0.5	35795
rs3774291	3/187916160	T/C	-0.031	1.2E-09	0.3	29034	0.011	3.5E-01	0.3	6624	-0.025	9.6E-08	0.5	35658
rs3774292	3/187916272	T/A	-0.028	4.7E-09	0.5	29344	0.013	2.7E-01	0.2	6624	-0.022	3.8E-07	0.5	35968
rs10440056	3/187916512	T/C	-0.028	4.8E-09	0.5	29343	0.013	2.7E-01	0.2	6624	-0.022	3.8E-07	0.5	35967
rs3821815	3/187916960	T/C	0.028	6.4E-09	0.4	29345	-0.007	5.7E-01	0.2	5941	0.024	1.2E-07	0.5	35286
rs1851665	3/187919088	G/A	-0.028	8.7E-09	0.5	29344	0.013	2.7E-01	0.2	6624	-0.022	6.1E-07	0.5	35968
rs1836860	3/187919296	T/C	0.028	7.3E-09	0.5	29342	-0.008	5.2E-01	0.2	5941	0.023	1.5E-07	0.5	35283
rs1624230	3/187921632	C/A	0.024	1.3E-06	0.3	27699	-0.007	5.8E-01	0.1	5941	0.02	1.4E-05	0.4	33640
rs1403694	3/187922688	T/C	0.028	1.5E-09	0.4	29345	-0.013	2.6E-01	0.2	6624	0.022	1.7E-07	0.5	35969
rs1648722	3/187931680	T/C	-0.032	3.4E-12	0.4	29263	0.006	6.2E-01	0.1	6624	-0.027	2.5E-10	0.5	35887
rs1656925	3/187931824	T/C	0.031	1.3E-11	0.4	29345	-0.01	4.1E-01	0.0	4963	0.026	1.1E-09	0.4	34308
rs1648698	3/187932272	G/C	0.031	1.3E-11	0.4	29345	-0.005	6.8E-01	0.0	6624	0.026	6.0E-10	0.4	35969
rs1648700	3/187932608	T/C	0.031	1.8E-11	0.3	29342	0	9.7E-01	0.1	5941	0.027	2.2E-10	0.4	35283
rs1624569	3/187932768	T/C	0.031	1.3E-11	0.4	29342	0	9.7E-01	0.1	5941	0.027	1.7E-10	0.4	35283
rs710448	3/187935584	G/A	-0.031	2.2E-11	0.4	29328	0.006	5.8E-01	0.1	6624	-0.026	1.3E-09	0.5	35952
rs5030060	3/187935616	T/C	0.029	2.2E-09	0.4	29340	-0.015	2.1E-01	0.2	6624	0.023	3.0E-07	0.5	35964
rs822363	3/187935680	G/C	-0.031	3.1E-11	0.4	29341	0.007	5.6E-01	0.1	6624	-0.026	1.9E-09	0.5	35965
rs3856930	3/187941024	T/C	0.03	2.3E-10	0.5	29231	0.002	9.0E-01	0.0	5941	0.027	1.7E-09	0.5	35172
rs5030091	3/187943568	T/C	0.038	9.7E-14	0.4	28827	-0.005	6.5E-01	0.6	6475	0.032	1.6E-11	0.6	35302
rs2062632	3/187943872	T/C	0.055	2.5E-19	0.1	29028	-0.019	1.8E-01	0.7	6624	0.044	5.1E-15	0.6	35652
rs266760	3/187943904	G/A	0.05	3.6E-13	0.0	20331	-0.028	5.7E-02	0.6	4554	0.037	3.7E-09	0.6	24885
rs1972703	3/187946032	G/A	0.057	7.0E-16	0.1	29094	-0.009	6.0E-01	0.6	5941	0.048	1.9E-13	0.5	35035
rs266764	3/187946656	G/C	-0.022	2.8E-06	0.3	29171	0.002	8.6E-01	0.3	5941	-0.019	1.4E-05	0.3	35112
rs6807774	3/187946752	G/A	0.024	7.1E-07	0.1	29152	-0.005	6.4E-01	0.0	6624	0.02	1.0E-05	0.2	35776
rs822368	3/187949552	G/A	-0.023	8.9E-07	0.3	29346	0.013	2.7E-01	0.5	6624	-0.018	2.7E-05	0.5	35970
rs822373	3/187953888	G/A	0.029	1.8E-09	0.0	29347	-0.005	6.7E-01	0.2	6624	0.024	5.8E-08	0.3	35971
rs266749	3/187954608	T/C	0.032	1.7E-10	0.1	29208	0.002	8.8E-01	0.1	5941	0.028	1.5E-09	0.2	35149
rs266743	3/187958480	T/C	-0.047	5.7E-14	0.2	29270	-0.052	7.7E-04	0.4	6475	-0.048	1.7E-16	0.2	35745
rs266742	3/187958544	T/G	-0.035	2.9E-12	0.0	29119	0.012	3.5E-01	0.6	6612	-0.029	4.4E-10	0.4	35731
rs822355	3/187962816	T/C	-0.047	2.2E-16	0.0	26923	0.024	7.7E-02	0.6	5792	-0.037	3.5E-12	0.6	32715
rs822354	3/187962896	G/A	-0.05	1.5E-20	0.0	28533	0.025	6.5E-02	0.6	5941	-0.04	8.6E-16	0.6	34474
rs266733	3/187976000	T/G	0.047	4.0E-22	0.3	28655	0.043	5.3E-04	0.0	5941	0.046	6.7E-25	0.2	34596
rs185554	3/187977120	G/A	0.052	3.1E-27	0.3	28802	0.044	1.0E-04	0.2	6624	0.051	1.2E-30	0.2	35426
rs3900626	3/187984592	T/C	-0.045	5.7E-13	0.1	29309	-0.026	9.1E-02	0.0	6624	-0.042	1.9E-13	0.0	35933
rs3816112	3/187985360	T/C	-0.042	2.0E-07	0.0	29346	-0.005	8.2E-01	0.0	6624	-0.039	4.8E-07	0.0	35970
rs1426810	3/187986128	G/A	0.054	2.8E-30	0.5	29345	0	9.7E-01	0.8	5941	0.047	3.6E-27	0.7	35286
rs1354091	3/187988592	T/G	0.046	9.7E-20	0.1	29345	0.034	1.6E-02	0.0	5941	0.045	5.0E-21	0.0	35286
rs17299991	3/187988624	T/C	-0.083	3.6E-07	0.0	26260	-0.019	6.1E-01	0.0	4703	-0.073	8.7E-07	0.0	30963
rs2066500	3/187990624	T/C	-0.047	9.7E-20	0.0	29344	-0.034	1.6E-02	0.0	5941	-0.045	4.8E-21	0.0	35285
rs266759	3/187991008	T/C	-0.052	2.0E-29	0.3	29331	-0.042	1.6E-04	0.2	6624	-0.05	1.2E-32	0.3	35955
rs266754	3/187991664	T/C	-0.052	2.3E-29	0.4	29288	-0.043	1.1E-04	0.2	6624	-0.051	9.8E-33	0.3	35912
rs187868	3/187992208	G/A	0.052	1.2E-29	0.4	29329	0.044	8.1E-05	0.2	6624	0.051	3.7E-33	0.3	35953
rs3917117	3/187998544	G/A	0.048	3.5E-20	0.0	29298	0.032	1.6E-02	0.0	6624	0.046	2.4E-21	0.0	35922
rs843991	3/187999120	T/C	0.053	2.7E-30	0.3	29298	-0.003	8.2E-01	0.8	5941	0.046	9.9E-27	0.7	35239
rs3917110	3/188001344	G/C	-0.048	1.5E-20	0.0	29332	-0.032	1.5E-02	0.0	6624	-0.046	9.9E-22	0.0	35956
rs3917109	3/188001504	C/A	0.048	5.2E-15	0.0	20655	0.03	3.8E-02	0.0	4554	0.045	7.8E-16	0.0	25209
rs16861184	3/188003168	T/C	-0.049	9.6E-21	0.0	29331	-0.034	1.1E-02	0.0	6624	-0.047	4.4E-22	0.0	35955
rs710450	3/188005328	C/A	-0.048	2.0E-19	0.3	20636	-0.046	4.0E-04	0.1	4554	-0.048	2.7E-22	0.3	25190
rs2293243	3/188005424	T/A	-0.049	5.3E-21	0.0	29326	-0.035	9.3E-03	0.0	6624	-0.047	1.9E-22	0.0	35950
rs13094303	3/188016800	T/C	-0.027	5.4E-08	0.3	29018	-0.016	1.9E-01	0.0	6554	-0.026	2.7E-08	0.1	35572
rs16861189	3/188018128	G/C	-0.027	4.8E-08	0.3	28985	-0.016	1.9E-01	0.0	6624	-0.026	2.4E-08	0.1	35609
rs6810075	3/188031264	T/C	0.066	3.6E-41	0.0	29140	0.043	5.8E-04	0.0	6609	0.063	1.8E-43	0.0	35749
rs10937273	3/188032384	G/A	-0.04	9.6E-18	0.3	29347	0.005	6.4E-01	0.3	6624	-0.034	3.6E-15	0.5	35971
rs12637534	3/188032896	G/A	-0.039	2.1E-06	0.2	26530	0.005	8.2E-01	0.2	5926	-0.035	8.4E-06	0.3	32456
rs1648707	3/188034400	C/A	-0.063	8.6E-40	0.0	29345	-0.043	2.7E-04	0.0	6624	-0.06	1.6E-42	0.0	35969
rs864265	3/188036992	T/G	-0.037	1.4E-08	0.3	29207	0.009	6.1E-01	0.7	5792	-0.031	2.4E-07	0.5	34999
rs16861194	3/188042112	G/A	-0.043	2.0E-07	0.4	26516	-0.002	9.2E-01	0.0	6624	-0.038	7.6E-07	0.3	33140

rs266729	3/188042176	G/C	-0.06	3.3E-29	0.0	29195	-0.04	1.9E-03	0.0	6624	-0.057	3.8E-31	0.0	35819
rs182052	3/188043472	G/A	0.064	6.7E-41	0.0	29304	0.048	2.0E-04	0.0	5941	0.062	5.2E-44	0.0	35245
rs16861205	3/188044320	G/A	0.047	2.3E-07	0.1	26456	-0.006	8.3E-01	0.0	5941	0.041	1.3E-06	0.2	32397
rs12495941	3/188050880	T/G	0.041	6.2E-12	0.0	28307	-0.022	1.0E-01	0.0	6475	0.031	7.5E-09	0.4	34782
rs3821799	3/188054176	T/C	0.03	3.3E-10	0.3	28972	0	1.0E+00	0.1	5941	0.026	3.3E-09	0.4	34913
rs1063538	3/188056880	T/C	0.064	6.2E-30	0.5	20404	-0.009	4.3E-01	0.8	6573	0.051	1.8E-23	0.8	26977
rs7615090	3/188073696	T/G	0.058	2.8E-11	0.4	21869	0.047	9.3E-02	0.0	3902	0.057	5.7E-12	0.3	25771
rs2412664	4/56152144	C/A	0.026	1.1E-06	0.0	29346	-0.005	7.3E-01	0.2	6624	0.022	8.8E-06	0.2	35970
rs13434995	4/56161972	G/A	-0.027	1.3E-06	0.1	29310	0.006	6.9E-01	0.6	5941	-0.023	8.7E-06	0.4	35251
rs10018932	4/56162624	G/A	0.027	1.6E-06	0.0	29309	-0.004	7.9E-01	0.6	6624	0.023	1.1E-05	0.3	35933
rs10011801	4/56163492	T/A	-0.027	1.7E-06	0.0	29309	0.003	8.0E-01	0.6	6624	-0.023	1.1E-05	0.3	35933
rs10000512	4/56163780	C/A	0.027	1.6E-06	0.0	29309	-0.003	8.0E-01	0.6	6624	0.023	1.1E-05	0.3	35933
rs17725110	4/56166780	G/A	-0.027	1.6E-06	0.0	29309	0.004	7.8E-01	0.6	6624	-0.023	1.1E-05	0.3	35933
rs17725163	4/56170204	T/C	0.027	1.8E-06	0.0	29305	-0.008	6.0E-01	0.7	5941	0.023	1.4E-05	0.4	35246
rs10026315	4/56173120	C/A	-0.027	3.9E-06	0.1	27600	0.008	5.9E-01	0.7	5941	-0.023	3.4E-05	0.4	33541
rs3805385	4/56174744	G/A	-0.027	2.0E-06	0.0	29309	0.005	7.3E-01	0.6	6623	-0.022	1.5E-05	0.4	35932
rs17781708	4/56175824	T/A	0.027	1.9E-06	0.0	29304	-0.008	6.1E-01	0.7	5941	0.023	1.5E-05	0.4	35245
rs702634	5/53307176	G/A	0.022	4.9E-06	0.0	29123	0.021	8.6E-02	0.6	6654	0.022	9.7E-07	0.2	35777
rs4865796	5/53308420	G/A	0.022	4.2E-06	0.0	29143	0.008	5.1E-01	0.7	6599	0.02	5.5E-06	0.4	35742
rs1664781	5/53312056	G/A	0.022	4.9E-06	0.1	29220	-0.024	2.2E-01	0.0	1587	0.019	2.7E-05	0.2	30807
rs6898870	5/53328864	G/A	0.026	5.4E-07	0.0	29305	0.014	2.4E-01	0.5	7362	0.024	3.6E-07	0.2	36667
rs3776720	5/53331916	G/A	-0.026	4.6E-07	0.0	29317	-0.014	2.5E-01	0.5	7362	-0.024	3.2E-07	0.3	36679
rs12521454	5/53333060	T/C	0.028	2.6E-07	0.1	29320	0.017	1.7E-01	0.6	7362	0.026	1.2E-07	0.3	36682
rs7736354	5/53333348	T/C	-0.026	5.1E-07	0.1	29334	-0.015	2.1E-01	0.6	7362	-0.025	2.9E-07	0.3	36696
rs7735253	5/53333368	G/A	0.026	5.1E-07	0.1	29335	0.015	2.1E-01	0.6	7362	0.025	2.9E-07	0.3	36697
rs6450175	5/53333580	T/C	-0.026	5.1E-07	0.1	29335	-0.015	2.1E-01	0.6	7362	-0.025	2.9E-07	0.3	36697
rs6450176	5/53333784	G/A	0.028	1.4E-07	0.1	29239	0.017	1.2E-01	0.4	10507	0.026	5.8E-08	0.2	39746
rs3776717	5/53334520	G/A	0.026	4.8E-07	0.1	29343	0.016	1.8E-01	0.6	7362	0.025	2.4E-07	0.3	36705
rs3776716	5/53334680	G/A	-0.026	5.0E-07	0.1	29343	-0.016	1.8E-01	0.6	7362	-0.025	2.5E-07	0.3	36705
rs1445887	5/53335132	T/A	-0.026	5.8E-07	0.1	29343	-0.017	1.2E-01	0.4	10521	-0.024	2.0E-07	0.3	39864
rs6893522	5/53335192	T/C	0.049	1.3E-06	0.0	29319	-0.009	7.5E-01	0.0	5940	0.042	8.3E-06	0.0	35259
rs4588541	5/53336396	G/A	-0.026	7.3E-07	0.1	29343	-0.016	1.9E-01	0.6	7362	-0.024	3.5E-07	0.3	36705
rs4311394	5/53336420	G/A	-0.026	7.1E-07	0.2	29344	-0.016	1.9E-01	0.6	7362	-0.024	3.4E-07	0.3	36706
rs10805455	5/53338060	T/G	0.026	7.5E-07	0.1	29343	0.016	1.9E-01	0.5	7362	0.024	3.7E-07	0.3	36705
rs2448	5/53338112	T/C	0.025	6.1E-07	0.1	29340	0.014	2.4E-01	0.5	7362	0.024	4.0E-07	0.3	36702
rs12654393	5/53338376	T/G	-0.025	9.2E-07	0.1	29342	-0.024	6.2E-02	0.5	6679	-0.025	1.4E-07	0.3	36021
rs6886510	5/53338884	T/C	-0.026	6.3E-07	0.1	29317	-0.023	7.1E-02	0.5	6679	-0.026	1.1E-07	0.3	35996
rs6876198	5/53339352	T/C	0.026	7.5E-07	0.1	29331	0.023	6.8E-02	0.5	6679	0.025	1.2E-07	0.3	36010
rs1541681	5/53340376	T/G	-0.025	1.1E-06	0.1	29314	-0.011	3.4E-01	0.5	7362	-0.023	1.1E-06	0.3	36676
rs3776706	5/53340688	T/G	0.025	1.6E-06	0.1	29314	0.013	2.7E-01	0.5	7362	0.023	1.1E-06	0.3	36676
rs3776705	5/53340712	T/C	0.025	1.8E-06	0.1	29314	0.013	2.7E-01	0.5	7362	0.023	1.3E-06	0.3	36676
rs855362	6/9952975	T/C	-0.032	3.7E-06	0.0	29291	-0.008	6.4E-01	0.2	5940	-0.029	6.5E-06	0.0	35231
rs1206974	6/9955661	T/C	-0.032	4.0E-06	0.0	29346	0.004	8.1E-01	0.3	6623	-0.026	2.8E-05	0.0	35969
rs1206976	6/9956231	G/A	0.031	4.9E-06	0.0	29344	-0.025	2.6E-01	0.1	2269	0.027	4.6E-05	0.0	31613
rs1206977	6/9956405	T/C	0.031	4.8E-06	0.0	29344	-0.004	8.1E-01	0.3	6623	0.026	3.3E-05	0.0	35967
rs2327213	6/9958457	G/C	0.032	4.7E-06	0.0	29343	-0.004	8.1E-01	0.3	6622	0.026	3.3E-05	0.0	35965
rs6459464	6/9959288	T/G	-0.032	4.6E-06	0.0	29343	0.004	8.1E-01	0.3	6622	-0.026	3.2E-05	0.0	35965
rs1934774	6/9959868	G/A	0.032	4.6E-06	0.0	29343	-0.004	8.1E-01	0.3	6622	0.027	3.3E-05	0.0	35965
rs7741384	6/9960565	T/C	0.035	4.2E-06	0.0	29209	-0.003	8.6E-01	0.2	6623	0.029	2.9E-05	0.0	35832
rs4716055	6/9961905	T/G	-0.036	1.6E-06	0.0	27708	-0.007	6.9E-01	0.2	5941	-0.031	4.1E-06	0.0	33649
rs9396660	6/9962667	T/G	-0.032	4.4E-06	0.0	29343	0.004	8.1E-01	0.3	6623	-0.027	3.3E-05	0.0	35966
rs6908776	6/9966618	T/G	-0.032	5.0E-06	0.0	29343	0.024	2.6E-01	0.1	2269	-0.027	5.0E-05	0.0	31612
rs1340640	6/9981898	G/A	0.033	3.5E-06	0.0	29332	0.007	6.9E-01	0.2	5940	0.029	7.4E-06	0.0	35272
rs998584	6/43865872	C/A	0.03	5.8E-08	0.3	28167	0.017	1.9E-01	0.0	5941	0.028	3.3E-08	0.2	34108
rs6905288	6/43866852	G/A	0.024	1.7E-06	0.2	28111	0.02	9.3E-02	0.0	6679	0.024	3.7E-07	0.0	34790
rs1358980	6/43872528	T/C	-0.029	2.4E-07	0.3	28532	-0.02	5.1E-02	0.0	9836	-0.027	4.1E-08	0.1	38368
rs668459	6/139877376	T/C	0.021	5.0E-06	0.0	29342	0.016	3.0E-01	0.0	2269	0.02	2.7E-06	0.0	31611
rs628751	6/139880112	C/A	-0.021	3.5E-06	0.0	29145	-0.001	9.3E-01	0.0	5941	-0.019	9.9E-06	0.0	35086
rs643381	6/139881120	C/A	-0.021	3.4E-06	0.0	29133	-0.005	6.9E-01	0.0	6474	-0.019	7.1E-06	0.0	35607
rs592423	6/139882384	C/A	0.022	2.5E-06	0.0	29089	0.007	5.3E-01	0.0	6679	0.02	3.4E-06	0.0	35768
rs636202	6/139885280	T/C	-0.022	4.2E-06	0.0	29087	-0.002	8.4E-01	0.0	6623	-0.019	1.2E-05	0.0	35710
rs6821	7/129561952	T/C	-0.027	3.3E-06	0.0	29086	0.006	6.7E-01	0.0	6568	-0.022	4.1E-05	0.0	35654
rs12706917	7/129567216	C/A	0.024	3.1E-06	0.0	29243	-0.005	6.9E-01	0.0	6616	0.02	2.9E-05	0.0	35859
rs2980885	8/126543488	G/A	-0.029	4.0E-06	0.0	24006	0	9.9E-01	0.0	6624	-0.024	2.3E-05	0.0	30630
rs2980884	8/126543536	G/A	-0.025	1.3E-06	0.4	23882	-0.009	4.8E-01	0.0	5792	-0.023	1.7E-06	0.2	29674
rs2954018	8/126546336	T/C	0.029	4.8E-08	0.0	24074	0.012	3.9E-01	0.0	5941	0.027	5.7E-08	0.0	30015
rs2001845	8/126547824	G/A	0.03	1.2E-07	0.0	22438	0.012	3.9E-01	0.0	5941	0.027	1.6E-07	0.0	28379
rs4871603	8/126549552	T/C	0.026	1.8E-07	0.1	24047	0.007	5.7E-01	0.0	5941	0.024	3.4E-07	0.0	29988
rs2980880	8/126550152	G/A	-0.03	1.1E-08	0.0	24084	-0.015	2.0E-01	0.0	6624	-0.028	7.3E-09	0.0	30708
rs2980879	8/126550656	T/A	0.03	1.1E-08	0.0	24084	0.015	2.0E-01	0.0	6624	0.028	7.1E-09	0.0	30708
rs2980878	8/126550712	G/C	-0.03	1.6E-08	0.0	24074	-0.015	2.2E-01	0.1	6624	-0.027	1.2E-08	0.0	30698
rs2980876	8/126550880	T/C	0.03	1.2E-08	0.0	24078	0.015	2.0E-01	0.0	6624	0.027	7.9E-09	0.0	30702
rs2954020	8/126551136	G/A	0.03	6.4E-08	0.0	22448	0.014	3.0E-01	0.2	5941	0.028	6.1E-08	0.0	28389
rs2954025	8/126553648	T/C	0.027	7.2E-08	0.1	24070	0.009	4.6E-01	0.0	5941	0.025	1.1E-07	0.1	30011
rs2954026	8/126553712	T/G	-0.029	2.2E-08	0.0	24074	-0.014	2.3E-01	0.0	6624	-0.027	1.7E-08	0.0	30698
rs2980862	8/126553824	G/C	-0.029	5.9E-08	0.0	24075	-0.011	4.2E-01	0.0	5941	-0.026	7.8E-08	0.0	30016
rs7828113	8/126554648	G/A	0.027	6.9E-08	0.1	24070	0.012	2.9E-01	0.0	6624	0.025	6.7E-08	0.0	30694
rs7846466	8/126554712	T/C	-0.029	2.4E-08	0.0	24074	-0.01	3.9E-01	0.0	6621	-0.026	4.0E-08	0.0	30695
rs2980867	8/126556872	T/G	-0.028	1.3E-07	0.0	24042	-0.01	4.5E-01	0.0	5941	-0.026	1.7E-07	0.0	29983
rs2954030	8/126560488	T/C	-0.025	8.4E-07	0.1	24032	-0.021	6.4E-02	0.0	6624	-0.025	1.4E-07	0.0	30656
rs2954032	8/126562576	G/A	0.029	4.1E-08	0.0	24036	0.013	3.4E-01	0.0	5941	0.027	4.2E-08	0.0	29977
rs2954033	8/126562928	G/A	0.029	4.3E-08	0.0	24035	0.016	1.9E-01	0.0	6624	0.027	2.5E-08	0.0	30659
rs2980859	8/126568608	G/C	0.03	4.9E-08	0.0	23994	0.017	1.6E-01	0.0	6624	0.028	2.4E-08	0.0	30618
rs2980888	8/126576488	T/C	-0.033	3.9E-08	0.0	23587	-0.02	1.2E-01	0.0	6624	-0.031	1.4E-08	0.0	30211
rs2954038	8/126576568	C/A	-0.032	4.8E-08	0.0	23586	-0.02	1.3E-01	0.0	6624	-0.03	1.8E-08	0.0	30210
rs625476	9/13695973	T/C	0.022	4.4E-06	0.3	29338	0.001	9.5E-01	0.0	9833	0.019	2.0E-05	0.3	39171
rs2782980	10/115771520	T/C	-0.027	1.7E-06	0.0	29111	0.017	1.7E-01	0.0	6624	-0.02	1.0E-04	0.3	35735
rs2782982	10/115778488	G/A	0.023	1.8E-06	0.0	29309	-0.006	5.9E-01	0.4	6624	0.019	2.2E-05	0.2	35933
rs10787516	10/115803912	T/C	0.022	2.3E-06	0.1	29343	0.007	4.7E-01	0.2	10373	0.02	4.0E-06	0.2	39716
rs10885531	10/115804384	T/C	0.022	2.4E-06	0.1	29342	0.007	5.2E-01	0.3	7362	0.02	3.7E-06	0.2	36704
rs11023332	11/14740686	G/C	0.022	1.4E-06	0.0	29304	-0.001	9.5E-01	0.3	6623	0.019	6.8E-06	0.2	35927
rs11045163	12/20354792	G/A	0.023	5.9E-07	0.1	29233	-0.01	3.6E-01	0.3	6474	0.019	1.6E-05	0.3	35707
rs11045172	12/20361488	C/A	0.036	4.8E-08	0.2	27347	-0.012	4.2E-01	0.2	6623	0.029	2.0E-06	0.4	33970

rs7134375	12/20365024	C/A	-0.023	7.4E-07	0.1	29344	0.006	6.0E-01	0.1	6623	-0.019	8.6E-06	0.2	35967
rs12812995	12/20369140	G/C	-0.028	3.1E-08	0.1	28949	0.009	4.5E-01	0.1	6623	-0.022	1.0E-06	0.3	35572
rs1444636	12/20384150	G/C	-0.026	3.4E-08	0.1	29124	0	9.9E-01	0.0	5941	-0.023	1.9E-07	0.2	35065
rs10770643	12/20385100	G/A	0.026	2.7E-08	0.1	29216	-0.006	5.8E-01	0.1	6623	0.022	6.2E-07	0.3	35839
rs7303397	12/20385638	G/A	0.026	4.2E-08	0.1	29236	-0.007	5.7E-01	0.1	6623	0.021	9.3E-07	0.3	35859
rs11045182	12/20386924	T/G	0.026	5.1E-08	0.2	28201	-0.007	5.6E-01	0.0	6623	0.022	1.1E-06	0.3	34824
rs7955516	12/20389304	C/A	0.026	2.4E-08	0.1	29178	0.009	4.2E-01	0.0	9098	0.024	4.5E-08	0.0	38276
rs2120757	12/20393268	T/C	-0.026	4.0E-08	0.2	29262	-0.002	9.0E-01	0.0	5941	-0.023	1.7E-07	0.2	35203
rs12815593	12/20395904	T/C	0.025	5.3E-08	0.2	29284	0.003	8.2E-01	0.0	5941	0.023	1.8E-07	0.1	35225
rs3943606	12/20399304	T/C	0.026	4.9E-08	0.3	29346	-0.008	4.7E-01	0.1	6623	0.021	1.4E-06	0.4	35969
rs1025949	12/20399854	G/A	-0.027	2.6E-06	0.2	27201	0.008	5.4E-01	0.2	6474	-0.022	3.8E-05	0.4	33675
rs2009084	12/20402460	T/C	-0.027	4.6E-08	0.3	27845	0.009	4.6E-01	0.1	6623	-0.022	1.6E-06	0.4	34468
rs17376795	12/20403322	T/C	0.026	1.3E-07	0.3	27859	-0.008	5.1E-01	0.2	6623	0.021	3.2E-06	0.4	34482
rs1319631	12/20406734	G/A	0.026	1.7E-07	0.3	27802	-0.008	5.0E-01	0.2	6623	0.021	4.0E-06	0.4	34425
rs2657901	12/55219684	G/A	-0.023	6.4E-07	0.0	29343	0.014	2.1E-01	0.0	6623	-0.018	2.6E-05	0.2	35966
rs2657902	12/55219920	G/A	0.023	6.5E-07	0.0	29343	-0.013	2.4E-01	0.0	6623	0.018	2.4E-05	0.2	35966
rs2694909	12/55221196	T/C	0.023	6.3E-07	0.0	29344	-0.015	2.0E-01	0.0	6623	0.018	2.7E-05	0.2	35967
rs7310284	12/55221920	G/A	-0.023	1.1E-06	0.0	29293	0.007	5.8E-01	0.0	5929	-0.02	1.0E-05	0.0	35222
rs2657888	12/55224648	T/G	0.023	5.7E-07	0.0	29326	-0.01	4.2E-01	0.0	5941	0.019	8.4E-06	0.0	35267
rs17540509	12/55230108	T/C	-0.023	1.2E-06	0.0	29292	0.015	2.0E-01	0.0	6623	-0.018	4.7E-05	0.1	35915
rs11171894	12/55237488	G/A	0.025	8.7E-07	0.0	29127	-0.011	3.6E-01	0.0	6623	0.02	1.8E-05	0.1	35750
rs1916334	12/120968680	G/A	0.028	3.2E-06	0.3	29206	0.02	1.7E-01	0.1	6623	0.027	1.2E-06	0.2	35829
rs11057405	12/121347848	G/A	0.052	5.6E-09	0.2	29329	-0.013	5.1E-01	0.5	6623	0.042	3.1E-07	0.4	35952
rs12369179	12/121529504	T/C	-0.052	1.7E-07	0.2	26479	0.016	4.6E-01	0.6	5941	-0.04	7.1E-06	0.5	32420
rs2454722	12/121737168	G/A	0.039	5.7E-11	0.0	29346	-0.001	9.3E-01	0.3	6623	0.033	1.0E-09	0.3	35969
rs601339	12/121740696	G/A	0.039	3.9E-11	0.0	29325	-0.002	9.0E-01	0.3	6622	0.034	7.8E-10	0.3	35947
rs4759361	12/121744232	T/A	-0.039	4.5E-11	0.0	29316	0.002	8.7E-01	0.3	6623	-0.033	9.5E-10	0.3	35939
rs509548	12/121747808	T/A	-0.039	4.7E-11	0.0	29314	0.004	8.1E-01	0.3	6623	-0.033	1.1E-09	0.3	35937
rs10846527	12/122609248	G/C	0.044	2.6E-06	0.0	29253	0.037	1.5E-01	0.0	5940	0.043	8.3E-07	0.0	35193
rs6488898	12/122769784	G/A	-0.054	1.5E-09	0.3	29346	-0.043	8.0E-02	0.0	6623	-0.053	2.8E-10	0.1	35969
rs7131750	12/122824840	T/C	-0.023	1.1E-06	0.3	29221	-0.026	3.3E-02	0.0	5941	-0.023	1.0E-07	0.2	35162
rs10846557	12/122831008	T/C	-0.022	1.3E-06	0.3	29315	-0.022	4.6E-02	0.0	6623	-0.022	1.6E-07	0.2	35938
rs11057353	12/122831640	T/C	-0.023	2.8E-06	0.4	29340	-0.028	1.3E-02	0.0	6623	-0.023	1.3E-07	0.2	35963
rs11057354	12/122833592	G/A	0.043	2.0E-09	0.2	29347	0.028	1.6E-01	0.0	5941	0.042	7.8E-10	0.1	35288
rs10846559	12/122840424	G/A	0.022	4.5E-06	0.4	29347	0.035	3.0E-03	0.0	6623	0.024	8.2E-08	0.3	35970
rs10773036	12/122842864	T/C	0.022	1.1E-06	0.4	29327	0.027	1.4E-02	0.0	6623	0.023	4.8E-08	0.2	35950
rs9888323	12/122844760	T/C	0.022	2.1E-06	0.4	29174	0.027	1.4E-02	0.0	6623	0.023	1.0E-07	0.2	35797
rs1114629	12/122846272	C/A	-0.022	2.4E-06	0.3	29171	-0.028	1.3E-02	0.0	6623	-0.023	1.1E-07	0.2	35794
rs4930731	12/122883368	G/A	-0.021	4.8E-06	0.2	29211	-0.022	5.6E-02	0.0	6623	-0.022	6.8E-07	0.1	35834
rs12317176	12/122970672	T/C	-0.026	2.5E-08	0.0	29334	0.017	1.5E-01	0.5	6623	-0.021	2.4E-06	0.4	35957
rs7301953	12/122971824	G/A	-0.027	1.8E-08	0.0	29309	0.02	9.7E-02	0.5	6623	-0.021	2.8E-06	0.4	35932
rs12322694	12/122973248	T/A	-0.035	7.7E-08	0.0	29255	0.01	5.9E-01	0.1	5941	-0.03	8.9E-07	0.2	35196
rs10846579	12/122973360	T/C	0.026	2.9E-08	0.0	29334	-0.018	1.3E-01	0.5	6623	0.02	3.2E-06	0.4	35957
rs11057394	12/122973632	T/C	-0.029	7.3E-09	0.0	27698	0.018	1.3E-01	0.5	6623	-0.022	1.6E-06	0.4	34321
rs12809125	12/122973944	G/A	0.026	2.9E-08	0.0	29334	-0.018	1.3E-01	0.5	6623	0.02	3.2E-06	0.4	35957
rs7133378	12/122975456	G/A	-0.03	1.3E-09	0.0	29223	0.024	5.0E-02	0.6	6474	-0.023	6.2E-07	0.5	35697
rs9971695	12/122979440	G/A	-0.026	2.5E-08	0.0	29334	0.018	1.2E-01	0.5	6623	-0.02	3.1E-06	0.4	35957
rs3802999	12/122980048	T/C	0.026	2.6E-08	0.0	29334	-0.018	1.2E-01	0.5	6623	0.02	3.3E-06	0.4	35957
rs4930721	12/122983840	T/C	0.027	1.5E-08	0.0	29309	-0.021	8.2E-02	0.5	6623	0.021	2.9E-06	0.4	35932
rs12298484	12/122984624	T/C	0.027	2.1E-08	0.0	29344	-0.004	7.8E-01	0.5	5941	0.023	1.9E-07	0.2	35285
rs11057396	12/122985016	C/A	0.031	4.6E-08	0.0	20631	-0.021	7.9E-02	0.6	6474	0.022	2.0E-05	0.5	27105
rs11057397	12/122985680	T/C	0.027	1.6E-08	0.0	29321	-0.018	1.2E-01	0.6	6474	0.021	2.1E-06	0.5	35795
rs9863	12/122987408	T/C	-0.027	3.3E-08	0.0	29065	0.022	5.8E-02	0.3	6602	-0.02	8.2E-06	0.4	35667
rs4930723	12/122989552	G/C	-0.027	2.0E-08	0.0	29333	0.018	1.2E-01	0.5	6623	-0.021	2.6E-06	0.4	35956
rs4930724	12/122989768	T/C	-0.026	5.5E-08	0.0	29316	0.004	7.3E-01	0.4	5941	-0.023	4.7E-07	0.2	35257
rs11057401	12/122993256	T/A	-0.028	6.9E-09	0.0	29302	0.009	4.9E-01	0.5	5941	-0.024	1.4E-07	0.2	35243
rs4930726	12/122994288	T/C	-0.027	1.5E-08	0.0	29332	0.017	1.5E-01	0.6	6622	-0.021	1.8E-06	0.5	35954
rs11057402	12/122996720	T/A	0.041	6.0E-08	0.1	29234	0.022	2.3E-01	0.1	6622	0.039	3.8E-08	0.1	35856
rs2178663	12/122999856	T/C	0.028	1.0E-08	0.0	29309	-0.021	8.4E-02	0.5	6623	0.021	2.1E-06	0.4	35932
rs4405410	12/123001744	T/A	-0.027	1.5E-08	0.0	29332	0.018	1.2E-01	0.5	6623	-0.021	2.2E-06	0.4	35955
rs7961449	12/123003168	T/A	-0.027	1.5E-08	0.0	29332	0.018	1.2E-01	0.5	6623	-0.021	2.2E-06	0.4	35955
rs7964945	12/123003624	T/A	-0.037	2.6E-08	0.0	29252	0.016	3.1E-01	0.0	6623	-0.03	1.3E-06	0.2	35875
rs3867146	12/123003960	G/A	0.041	2.5E-08	0.1	29316	0.02	3.2E-01	0.0	4553	0.039	2.0E-08	0.0	33869
rs7132655	12/123004928	G/C	-0.027	1.6E-08	0.0	29332	0.017	1.5E-01	0.6	6623	-0.021	1.8E-06	0.4	35955
rs4765219	12/123006064	C/A	-0.027	1.8E-08	0.0	29333	0.005	7.1E-01	0.5	5941	-0.023	1.8E-07	0.2	35274
rs7958691	12/123006696	T/G	0.027	1.7E-08	0.0	29332	-0.017	1.5E-01	0.6	6623	0.021	1.9E-06	0.4	35955
rs7305864	12/123007832	G/C	0.028	6.6E-09	0.0	29197	-0.005	7.2E-01	0.5	5941	0.024	7.8E-08	0.2	35138
rs10773048	12/123010360	T/C	0.023	1.4E-06	0.2	29148	0.013	2.9E-01	0.0	5941	0.022	9.7E-07	0.0	35089
rs6488913	12/123011520	G/C	-0.027	1.3E-08	0.0	29332	0.017	1.5E-01	0.6	6623	-0.021	1.6E-06	0.4	35955
rs7312404	12/123012680	G/A	0.032	1.8E-08	0.0	20655	-0.024	5.8E-02	0.7	4553	0.023	7.4E-06	0.5	25208
rs11831913	12/123012848	T/C	-0.028	6.7E-09	0.0	29308	0.018	1.3E-01	0.6	6623	-0.022	1.0E-06	0.4	35931
rs6488914	12/123013792	G/C	-0.027	1.5E-08	0.0	29298	0.005	6.9E-01	0.5	5941	-0.024	1.7E-07	0.2	35239
rs7973683	12/123015176	C/A	-0.027	8.4E-09	0.0	29345	0.015	2.1E-01	0.6	6623	-0.022	8.0E-07	0.5	35968
rs4765127	12/123026120	T/G	0.028	4.6E-09	0.0	29330	-0.003	8.3E-01	0.5	5941	0.024	4.2E-08	0.2	35271
rs12311114	12/123026656	C/A	-0.028	1.1E-08	0.0	29293	0.001	9.1E-01	0.6	5922	-0.024	7.5E-08	0.3	35215
rs4765528	12/123028208	T/A	-0.028	4.6E-09	0.0	29239	0.005	7.1E-01	0.5	5941	-0.025	5.6E-08	0.2	35180
rs11057408	12/123030792	T/G	0.027	1.1E-08	0.0	29297	-0.003	8.1E-01	0.5	5941	0.024	9.5E-08	0.2	35238
rs7978610	12/123034528	G/C	-0.03	1.6E-09	0.0	27709	0	9.9E-01	0.5	5941	-0.027	1.4E-08	0.2	33650
rs952632	12/123035688	G/A	0.028	4.1E-09	0.0	29342	-0.013	2.8E-01	0.6	6623	0.022	3.3E-07	0.4	35965
rs11837287	12/123036016	T/C	0.028	5.8E-09	0.0	29345	-0.012	2.9E-01	0.6	6623	0.022	4.2E-07	0.4	35968
rs7311969	12/123036288	T/C	-0.028	4.4E-09	0.0	29338	0	9.9E-01	0.5	5941	-0.025	2.8E-08	0.2	35279
rs7307277	12/123041112	G/A	0.028	5.8E-09	0.0	29341	-0.012	2.9E-01	0.6	6623	0.022	4.2E-07	0.4	35964
rs7311233	12/123041896	G/A	0.028	4.2E-09	0.0	29328	-0.001	9.3E-01	0.5	5941	0.025	3.2E-08	0.2	35269
rs12809473	12/123042552	T/A	-0.028	6.0E-09	0.0	29345	0.012	3.0E-01	0.6	6623	-0.022	4.2E-07	0.4	35968
rs12827409	12/123042656	G/C	0.028	6.1E-09	0.0	29345	-0.012	2.9E-01	0.6	6623	0.022	4.3E-07	0.4	35968
rs7135314	12/123044520	T/A	0.028	6.2E-09	0.0	29345	-0.012	2.9E-01	0.6	6623	0.022	4.4E-07	0.4	35968
rs825510	12/123044536	C/A	-0.039	3.3E-07	0.0	26597	-0.027	1.3E-01	0.0	6623	-0.037	1.1E-07	0.0	33220
rs4765148	12/123044592	T/G	0.028	6.6E-09	0.0	29323	-0.001	9.1E-01	0.6	5941	0.025	5.1E-08	0.3	35264
rs11057409	12/123045280	T/C	0.028	5.6E-09	0.0	29312	-0.001	9.7E-01	0.6	5941	0.025	3.9E-08	0.2	35253
rs7975482	12/123047640	G/A	0.028	7.7E-09	0.0	29268	0	9.7E-01	0.5	5941	0.024	5.1E-08	0.2	35209
rs2130382	12/123050592	G/C	0.028	8.3E-09	0.0	29331	-0.013	2.8E-01	0.6	6623	0.022	6.3E-07	0.4	35954
rs12310367	12/123052632	G/A	0.028	1.2E-08	0.0	29342	-0.012	3.2E-01	0.6	6623	0.022	7.4E-07	0.4	35965
rs11057412	12/123055096	G/C	-0.028	1.2E-08	0.0	29342	0.012	3.2E-01	0.6	6623	-0.022	7.8E-07	0.4	35965

rs1187415	12/123057480	G/C	0.028	9.6E-09	0.0	29340	0	9.7E-01	0.5	5941	0.024	6.5E-08	0.3	35281
rs707334	12/123058368	G/A	-0.039	4.4E-07	0.0	26596	-0.028	1.3E-01	0.0	6622	-0.038	1.4E-07	0.0	33218
rs12303671	12/123058560	T/G	-0.028	1.3E-08	0.0	29342	0.011	3.3E-01	0.6	6623	-0.022	7.5E-07	0.4	35965
rs7307053	12/123060496	T/C	0.028	1.6E-08	0.0	29306	-0.001	9.7E-01	0.5	5941	0.025	1.1E-07	0.2	35247
rs12824567	12/123061152	G/C	-0.028	1.5E-08	0.0	29305	0.013	2.7E-01	0.6	6623	-0.022	1.2E-06	0.4	35928
rs863750	12/123071400	T/C	-0.027	6.4E-09	0.0	29334	-0.003	8.2E-01	0.2	5941	-0.024	2.8E-08	0.0	35275
rs10773049	12/123072584	T/C	-0.027	2.7E-08	0.0	29329	0.003	7.9E-01	0.3	6623	-0.022	3.9E-07	0.2	35952
rs825453	12/123074712	T/A	-0.026	4.0E-08	0.0	29289	0.001	9.2E-01	0.3	6623	-0.022	4.0E-07	0.1	35912
rs11057418	12/123074928	G/C	0.034	3.1E-08	0.1	26176	0.021	1.8E-01	0.0	5941	0.033	1.4E-08	0.0	32117
rs1716407	12/123081168	G/A	0.025	9.1E-08	0.0	29255	-0.006	6.0E-01	0.6	6623	0.021	1.5E-06	0.2	35878
rs825456	12/123103528	G/A	0.021	4.3E-06	0.0	29313	-0.008	5.1E-01	0.7	6623	0.017	4.9E-05	0.4	35936
rs825459	12/123107040	G/C	-0.025	3.9E-07	0.0	29113	0.006	5.9E-01	0.7	6623	-0.02	5.8E-06	0.4	35736
rs4777845	15/91678432	T/C	-0.075	3.0E-06	0.0	24461	-0.012	7.3E-01	0.0	6624	-0.064	9.4E-06	0.0	31085
rs4777846	15/91680832	T/C	-0.074	3.1E-06	0.0	24459	-0.012	7.3E-01	0.0	6623	-0.064	9.6E-06	0.0	31082
rs2927328	16/80067224	T/C	-0.031	1.7E-10	0.4	29129	-0.029	1.4E-02	0.0	6577	-0.031	6.7E-12	0.3	35706
rs1471379	16/80068200	T/C	-0.03	3.2E-10	0.3	29255	-0.039	1.5E-03	0.0	5940	-0.031	2.3E-12	0.1	35195
rs1966957	16/80068576	G/C	-0.029	2.7E-10	0.3	29337	-0.032	4.1E-03	0.0	6623	-0.03	3.7E-12	0.1	35960
rs2927327	16/80069032	T/A	-0.029	2.5E-10	0.3	29337	-0.032	4.1E-03	0.0	6623	-0.03	3.5E-12	0.1	35960
rs2966079	16/80069304	T/C	0.031	1.6E-11	0.5	29184	0.035	1.5E-03	0.0	6571	0.032	8.8E-14	0.3	35755
rs1471152	16/80070136	T/G	-0.029	3.4E-10	0.3	29315	-0.038	1.8E-03	0.0	5940	-0.03	2.8E-12	0.1	35255
rs2927324	16/80070320	T/C	0.032	1.3E-11	0.5	29184	0.043	3.7E-04	0.0	5940	0.033	2.9E-14	0.3	35124
rs2927323	16/80070448	G/A	-0.034	4.8E-12	0.4	29308	-0.025	3.6E-02	0.0	6623	-0.033	5.1E-13	0.2	35931
rs2927322	16/80072008	G/A	0.038	4.9E-13	0.4	29016	0.026	3.7E-02	0.0	6474	0.036	6.1E-14	0.2	35490
rs2966085	16/80074136	G/A	0.033	5.7E-12	0.3	29004	0.031	1.7E-02	0.0	5791	0.033	2.7E-13	0.2	34795
rs2317241	16/80077264	G/A	0.033	5.4E-11	0.2	27946	0.027	2.6E-02	0.0	6623	0.032	4.2E-12	0.0	34569
rs12443634	16/80081776	C/A	0.043	1.6E-16	0.1	29157	0.035	5.6E-03	0.0	6623	0.042	2.9E-18	0.0	35780
rs7193307	16/80083800	G/A	-0.03	5.5E-07	0.0	27779	0.009	5.3E-01	0.0	6623	-0.024	1.1E-05	0.0	34402
rs12932649	16/80084336	T/C	0.03	1.3E-07	0.2	27777	-0.01	4.8E-01	0.0	6474	0.025	3.4E-06	0.3	34251
rs2927311	16/80088728	G/C	0.028	2.9E-07	0.0	29310	-0.005	7.2E-01	0.0	6623	0.024	3.4E-06	0.1	35933
rs4889326	16/80091160	T/C	0.028	3.4E-07	0.0	29320	-0.006	6.4E-01	0.0	6623	0.023	4.6E-06	0.1	35943
rs2925979	16/80092288	T/C	-0.044	1.9E-18	0.0	29347	-0.038	1.9E-03	0.0	6623	-0.043	1.2E-20	0.0	35970
rs4889327	16/80094152	T/C	0.027	1.0E-06	0.1	29346	-0.01	4.7E-01	0.0	5940	0.022	1.3E-05	0.2	35286
rs2966093	16/80096120	G/A	-0.04	1.4E-16	0.3	29162	-0.034	7.7E-03	0.0	5940	-0.04	3.1E-18	0.2	35102
rs2966094	16/80096136	C/A	0.041	5.5E-17	0.3	29162	0.032	6.9E-03	0.0	6623	0.04	1.4E-18	0.2	35785
rs12920728	16/80096344	G/A	-0.028	3.6E-07	0.1	29326	0.005	6.8E-01	0.0	6623	-0.023	4.5E-06	0.1	35949
rs8063007	16/80099088	C/A	-0.031	8.0E-07	0.0	29347	0.007	6.6E-01	0.4	6623	-0.026	8.4E-06	0.3	35970
rs2927307	16/80101336	G/A	-0.039	4.2E-17	0.4	29289	-0.035	1.8E-03	0.0	6623	-0.039	2.5E-19	0.3	35912
rs8182218	16/80105184	T/G	-0.027	9.7E-07	0.0	29327	0.006	6.5E-01	0.0	6623	-0.022	1.1E-05	0.1	35950
rs2966095	16/80106960	G/A	0.04	1.9E-16	0.3	29198	0.03	1.2E-02	0.0	6623	0.039	8.3E-18	0.2	35821
rs2966097	16/80107208	T/C	0.04	1.5E-14	0.4	27568	0.03	1.2E-02	0.0	6623	0.038	6.1E-16	0.2	34191
rs8057739	16/80107576	G/A	-0.032	1.4E-06	0.1	29223	0.011	4.9E-01	0.3	6623	-0.026	2.1E-05	0.3	35846
rs12934986	16/80110568	T/A	-0.031	1.4E-06	0.0	29206	0.01	5.4E-01	0.5	5940	-0.026	1.3E-05	0.3	35146
rs8191256	16/80691360	T/C	-0.128	1.5E-06	0.0	17330	-0.018	8.5E-01	0.0	1306	-0.12	2.3E-06	0.0	18636
rs11865200	16/81183208	G/A	-0.04	1.1E-09	0.4	29175	0.003	8.4E-01	0.0	6623	-0.034	2.2E-08	0.4	35798
rs7196910	16/81183880	G/C	-0.029	1.6E-08	0.5	29147	0.002	8.6E-01	0.0	6623	-0.025	1.9E-07	0.5	35770
rs8058318	16/81185744	G/A	0.028	3.3E-08	0.2	29100	-0.019	1.1E-01	0.0	6623	0.021	5.8E-06	0.4	35723
rs8047615	16/81191552	G/A	0.029	2.3E-08	0.5	29344	-0.001	9.2E-01	0.0	5940	0.026	1.5E-07	0.4	35284
rs12596316	16/81203656	G/A	-0.024	1.8E-07	0.1	29287	0.001	9.2E-01	0.0	5940	-0.021	9.5E-07	0.2	35227
rs3852725	16/81203672	C/A	-0.025	5.0E-08	0.4	29249	0.011	3.9E-01	0.1	5940	-0.021	1.2E-06	0.5	35189
rs8060301	16/81219248	T/A	0.025	3.3E-08	0.4	29309	0.008	5.0E-01	0.2	6623	0.023	6.0E-08	0.3	35932
rs4783244	16/81219768	T/G	-0.025	3.7E-08	0.4	29341	-0.008	4.9E-01	0.3	6623	-0.023	6.5E-08	0.4	35964
rs12051213	16/81220600	T/C	-0.027	8.1E-08	0.3	28603	-0.008	5.4E-01	0.0	6623	-0.025	1.6E-07	0.2	35226
rs12051272	16/81220792	T/G	-0.277	1.4E-49	0.5	15593	-0.053	4.0E-01	0.7	5471	-0.26	6.1E-48	0.7	21064
rs10514548	16/81224424	T/A	-0.025	1.4E-06	0.2	29039	-0.003	8.1E-01	0.4	6623	-0.022	4.6E-06	0.3	35662
rs9652670	16/81228976	G/C	0.025	9.3E-07	0.4	28893	0.013	2.7E-01	0.0	6623	0.023	6.7E-07	0.2	35516
rs4782722	16/81229664	T/G	0.026	3.0E-07	0.4	28995	0.021	1.1E-01	0.0	5791	0.025	7.4E-08	0.2	34786
rs12922394	16/81229824	T/C	-0.103	3.2E-18	0.3	24466	-0.005	8.4E-01	0.0	6623	-0.083	2.0E-15	0.4	31089
rs1870843	16/81316816	G/A	0.032	4.3E-11	0.2	29343	0.012	3.1E-01	0.2	6623	0.029	7.0E-11	0.2	35966
rs11150491	16/81318000	T/G	-0.031	2.2E-10	0.0	29298	-0.004	7.1E-01	0.2	6623	-0.027	1.6E-09	0.2	35921
rs10514560	16/81328576	G/A	-0.025	2.4E-06	0.0	29343	-0.008	5.4E-01	0.2	6623	-0.023	3.9E-06	0.0	35966
rs10514559	16/81328752	G/C	-0.025	1.2E-07	0.1	29291	-0.023	4.4E-02	0.2	6623	-0.025	1.4E-08	0.1	35914
rs12103370	16/81329144	T/C	-0.028	1.6E-07	0.4	29219	0.001	9.4E-01	0.6	6623	-0.023	1.4E-06	0.5	35842
rs11646073	16/81334512	A/G	-0.085	4.4E-06	0.0	16273	0.039	2.4E-01	0.3	6303	-0.056	4.9E-04	0.5	22576
rs1462046	16/81356624	T/C	-0.021	3.4E-06	0.0	29290	-0.012	2.7E-01	0.0	6623	-0.02	2.3E-06	0.0	35913
rs12446293	16/81380968	T/C	-0.026	2.5E-06	0.4	28957	-0.01	4.6E-01	0.5	6623	-0.024	3.4E-06	0.4	35580
rs9928039	16/81555240	G/C	-0.022	1.7E-06	0.0	29337	-0.006	6.2E-01	0.0	6623	-0.02	3.0E-06	0.0	35960
rs12929479	16/81555352	G/A	0.026	7.0E-08	0.2	27512	0.031	1.1E-02	0.0	5940	0.027	2.6E-09	0.0	33452
rs8064211	16/81558600	G/A	-0.021	2.5E-06	0.0	29336	-0.001	9.1E-01	0.0	5940	-0.019	6.6E-06	0.0	35276
rs8062451	16/81558640	G/A	0.021	4.4E-06	0.0	29344	0.004	7.3E-01	0.0	6623	0.018	9.4E-06	0.0	35967
rs8046196	16/81558848	T/G	-0.024	1.5E-07	0.0	29345	-0.024	3.4E-02	0.0	6623	-0.024	1.3E-08	0.0	35968
rs10492868	16/81559360	G/C	-0.022	1.6E-06	0.0	29258	-0.007	5.1E-01	0.0	6623	-0.02	2.3E-06	0.0	35881
rs8059783	16/81570344	T/G	-0.023	4.0E-07	0.0	29316	-0.004	7.4E-01	0.0	5938	-0.021	9.2E-07	0.0	35254
rs4782742	16/81573152	C/A	-0.023	5.3E-07	0.0	29338	-0.004	7.3E-01	0.2	5940	-0.021	1.2E-06	0.0	35278
rs8063602	16/81573920	C/A	-0.022	9.0E-07	0.0	29343	-0.008	4.7E-01	0.2	6623	-0.02	1.2E-06	0.0	35966
rs7404645	16/81578032	G/A	0.026	9.2E-07	0.0	29342	0.016	2.0E-01	0.0	6623	0.024	4.6E-07	0.0	35965
rs11150530	16/81583816	T/A	-0.027	9.0E-07	0.0	29143	-0.012	3.8E-01	0.0	5940	-0.025	9.1E-07	0.0	35083
rs6565099	16/81596480	T/C	-0.023	5.0E-07	0.0	29190	-0.006	6.3E-01	0.4	5940	-0.021	8.9E-07	0.1	35130
rs7206011	16/81596720	T/A	0.023	4.6E-07	0.0	29232	0.011	3.2E-01	0.4	6623	0.022	3.9E-07	0.1	35855
rs7203988	16/81600440	C/A	-0.024	1.8E-07	0.0	29343	-0.011	3.3E-01	0.4	6623	-0.022	1.6E-07	0.0	35966
rs7500448	16/81603288	G/A	0.027	1.4E-06	0.0	29084	0.013	3.3E-01	0.3	6623	0.025	1.3E-06	0.1	35707
rs12918209	16/81617616	T/C	0.029	5.6E-07	0.0	29045	0.008	5.3E-01	0.0	6623	0.025	1.3E-06	0.0	35668
rs4591132	16/81620520	T/C	-0.025	1.5E-06	0.0	29077	-0.012	3.5E-01	0.0	5868	-0.023	1.4E-06	0.0	34945
rs9888896	16/81622904	T/C	0.027	2.1E-06	0.0	29161	0.011	3.5E-01	0.0	6623	0.025	2.4E-06	0.0	35784
rs6565105	16/81623168	G/A	0.025	1.1E-07	0.3	29278	0.022	4.9E-02	0.0	6474	0.024	1.3E-08	0.1	35752
rs2228685	16/81623464	T/A	0.024	1.3E-07	0.3	29279	0.028	2.3E-02	0.0	5940	0.025	8.9E-09	0.0	35219
rs4366697	16/81625488	G/C	0.027	9.4E-07	0.0	28680	0.005	6.9E-01	0.0	6623	0.024	2.7E-06	0.0	35303
rs4473177	16/81627208	T/C	-0.028	1.9E-06	0.0	29124	-0.01	4.5E-01	0.0	5937	-0.025	2.6E-06	0.0	35061
rs4387591	16/81629920	G/C	-0.025	1.9E-06	0.0	28617	0.003	7.9E-01	0.2	6623	-0.021	1.4E-05	0.0	35240
rs4290460	16/81629960	C/A	-0.029	1.0E-06	0.0	28837	-0.005	7.2E-01	0.0	6474	-0.025	3.6E-06	0.0	35311
rs4294808	16/81636080	G/A	-0.024	3.7E-06	0.0	28705	-0.028	3.4E-02	0.1	6534	-0.025	3.6E-07	0.0	35239
rs13332706	16/81911760	G/A	0.112	3.3E-06	0.0	23418	-0.006	8.5E-01	0.0	6302	0.07	2.8E-04	0.1	29720
rs507506	17/7059046	G/A	-0.024	3.4E-06	0.1	29233	-0.014	1.5E-01	0.0	10520	-0.022	1.7E-06	0.0	39753
rs17805277	17/7142477	T/G	0.04	4.1E-07	0.0	26290	-0.009	6.3E-01	0.0	5940	0.033	5.9E-06	0.1	32230

rs889140	19/38580840	G/A	-0.029	9.8E-10	0.1	29347	0.005	6.9E-01	0.2	6624	-0.024	2.5E-08	0.3	35971
rs889139	19/38581208	G/A	0.029	1.1E-09	0.1	29291	0.001	9.2E-01	0.1	5941	0.026	6.8E-09	0.2	35232
rs731839	19/38590904	G/A	-0.037	2.2E-13	0.0	29166	0	9.9E-01	0.4	6605	-0.031	8.0E-12	0.4	35771
rs4805885	19/38597964	T/C	-0.033	1.3E-11	0.0	29137	0	9.9E-01	0.5	6613	-0.028	2.6E-10	0.3	35750
rs12461274	19/38599376	T/C	-0.022	3.6E-06	0.3	29211	-0.013	2.5E-01	0.5	6624	-0.02	2.1E-06	0.3	35835
rs8182584	19/38601552	T/G	-0.031	6.6E-11	0.0	29245	0.004	7.4E-01	0.5	6624	-0.026	2.7E-09	0.4	35869
rs6510382	19/38610408	T/G	-0.025	2.1E-07	0.0	29312	-0.003	8.1E-01	0.3	6623	-0.022	8.5E-07	0.2	35935
rs10417411	19/38611328	G/C	-0.025	2.8E-07	0.0	29320	-0.004	7.6E-01	0.2	6624	-0.022	9.4E-07	0.2	35944
rs3826904	19/38679696	G/C	-0.022	4.0E-06	0.1	29340	-0.003	8.1E-01	0.3	6624	-0.019	1.1E-05	0.2	35964
rs33824	19/38682280	T/C	0.022	3.7E-06	0.2	29345	0.01	4.3E-01	0.1	5941	0.02	3.4E-06	0.1	35286
rs33838	19/38701572	G/A	0.021	4.9E-06	0.1	29315	-0.016	4.1E-01	0.0	1587	0.019	1.9E-05	0.2	30902

*Denotes SNPs typed in the *de-novo* follow-up phase
