# Supplementary material for: Novel Loci for Adiponectin Levels and Their Influence on Type 2 Diabetes and Metabolic Traits: A Multi-Ethnic Meta-Analysis of 45,891 Individuals
Source: PLoS Genet. 2012 Mar 29;8(3):e1002607. doi: 10.1371/journal.pgen.1002607 (PMC3315470; doi:10.1371/journal.pgen.1002607)
Supplement: Table S4 — Genome-Wide Significant SNPs (p<5×10^−8^) Associated with Adiponectin Levels in Non-Europeans Populations. EA: Effect Allele, NEA: Non-Effect Allele, EA-Freq: Frequency of Effect Allele. (PDF) [file pgen.1002607.s007.pdf]

Table S4. **Genome-Wide Significant SNPs ($p < 5 \times 10^{-8}$) Associated with Adiponectin Levels in Non-Europeans Populations.**

snp	position	Closet Gene	EA/NEA	EA-freq	Beta (CI 95%)	SE	P value	N
**African American**								
rs4686807	3/188080297	*ADIPOQ*	A/G	0.316258	0.10(0.069,0.125)	0.014	1.61E-11	3,095
**East-Asian**								
rs822391	3/188046497	*ADIPOQ*	C/T	0.1514	0.15 (-0.190,0.306)	0.023	1.57E-10	1,776
rs12445207	16/81201259	*CDH13*	G/C	0.4478	0.49(-0.152,0.149)	0.014	4.17E-20	1,776

EA: Effect Allele
NEA: Non-Effect Allele
EA-Freq: Frequency of Effect Allele
