# Supplementary material for: Novel Loci for Adiponectin Levels and Their Influence on Type 2 Diabetes and Metabolic Traits: A Multi-Ethnic Meta-Analysis of 45,891 Individuals
Source: PLoS Genet. 2012 Mar 29;8(3):e1002607. doi: 10.1371/journal.pgen.1002607 (PMC3315470; doi:10.1371/journal.pgen.1002607)
Supplement: Table S5 — SNPs associated with adiponectin at genome-wide significant levels (p<5×10^−8^) using the fixed-effect model in women only in European populations (including Discovery and Follow-Up phases). (PDF) [file pgen.1002607.s008.pdf]

Table S5. **SNPs associated with adiponectin at genome-wide significant levels ($p < 5 \times 10^{-8}$) using the fixed-effect model in women only in European populations (including Discovery and Follow-Up phases).**

SNPs	Chr/Position	EA/NEA	Beta (95 CI %)	SE	P	I2	n
rs648514	3/52442303	G/A	0.035(0.023,0.047)	0.006	8.81E-09	0.28	16,638
rs1010553	3/52515813	T/C	0.034(0.022,0.045)	0.006	2.38E-08	0.24	16,602
rs1010554	3/52517959	T/C	0.036(0.024,0.047)	0.006	2.65E-09	0.29	16,677
rs13081028	3/52530356	G/A	-0.041(-0.052,-0.029)	0.006	1.85E-11	0.26	16,645
rs9853056	3/52530997	T/C	-0.041(-0.052,-0.029)	0.006	1.62E-11	0.25	16,643
rs13303	3/52533048	T/C	-0.041(-0.054,-0.028)	0.007	2.91E-09	0.13	12,326
rs4282054	3/52541105	T/C	-0.041(-0.052,-0.029)	0.006	1.66E-11	0.25	16,643
rs7614981	3/52541954	C/A	-0.036(-0.047,-0.024)	0.006	2.55E-09	0.31	16,645
rs12489828	3/52542054	T/G	0.036(0.024,0.047)	0.006	2.55E-09	0.31	16,644
rs2590838	3/52597126	G/A	-0.04(-0.051,-0.028)	0.006	2.39E-11	0.06	16,677
rs2276824	3/52612526	G/C	0.04(0.028,0.052)	0.006	3.17E-10	0.23	16,288
rs9879090	3/52623305	T/C	-0.04(-0.052,-0.028)	0.006	2.84E-10	0.23	16,288
rs13083798	3/52624788	G/A	0.04(0.029,0.052)	0.006	1.72E-11	0.05	16,678
rs1108842	3/52695120	C/A	0.04(0.029,0.052)	0.006	1.23E-11	0.05	16,676
rs11235	3/52720127	T/C	0.04(0.028,0.052)	0.006	3.25E-10	0.23	16,290
rs2710323	3/52790945	T/C	-0.038(-0.05,-0.027)	0.006	1.22E-10	0.04	16,683
rs4481150	3/52812833	T/C	-0.035(-0.046,-0.023)	0.006	7.30E-09	0	16,664
rs2535627	3/52820145	T/C	-0.035(-0.047,-0.023)	0.006	5.69E-09	0	16,680
rs2071044	3/52822641	T/C	0.035(0.023,0.046)	0.006	7.04E-09	0	16,630
rs5030062	3/187936874	C/A	0.04(0.029,0.052)	0.006	4.64E-11	0.45	16,327
rs5030072	3/187938240	T/C	-0.035(-0.047,-0.023)	0.006	7.26E-09	0.54	16,536
rs3856930	3/187941016	T/C	0.038(0.025,0.05)	0.006	1.96E-09	0.23	16,569
rs698078	3/187941921	G/A	0.042(0.03,0.053)	0.006	3.32E-12	0.43	16,634
rs710446	3/187942621	T/C	-0.04(-0.052,-0.029)	0.006	1.83E-11	0.44	16,656
rs5030091	3/187943571	T/C	0.041(0.028,0.054)	0.007	1.82E-09	0.16	16,165
rs2062632	3/187943875	T/C	0.055(0.039,0.07)	0.008	5.84E-12	0	16,366
rs266760	3/187943910	G/A	0.056(0.039,0.072)	0.009	2.05E-10	0	12,028
rs1972703	3/187946037	G/A	0.056(0.038,0.073)	0.009	1.28E-09	0	16,431
rs822355	3/187962817	T/C	-0.045(-0.06,-0.031)	0.007	1.07E-09	0	15,075
rs822354	3/187962900	G/A	-0.048(-0.062,-0.035)	0.007	5.88E-12	0	15,890
rs266733	3/187976007	T/G	0.044(0.032,0.056)	0.006	4.39E-12	0.34	15,998
rs185554	3/187977116	G/A	0.049(0.037,0.061)	0.006	1.06E-14	0.33	16,139
rs266719	3/187984342	T/C	0.072(0.058,0.086)	0.007	2.08E-22	0.58	16,508
rs1426810	3/187986129	G/A	0.051(0.039,0.063)	0.006	9.16E-17	0.53	16,681
rs1354091	3/187988594	T/G	0.042(0.03,0.055)	0.007	1.62E-10	0	16,684
rs2066500	3/187990616	T/C	-0.043(-0.056,-0.03)	0.007	9.90E-11	0	16,682
rs266759	3/187991006	T/C	-0.05(-0.062,-0.039)	0.006	2.63E-17	0.22	16,669
rs266756	3/187991268	G/C	0.074(0.059,0.088)	0.007	6.55E-23	0.57	16,471
rs266754	3/187991660	T/C	-0.051(-0.062,-0.039)	0.006	5.00E-17	0.24	16,626
rs187868	3/187992211	G/A	0.051(0.039,0.062)	0.006	1.86E-17	0.23	16,667
rs3917117	3/187998538	G/A	0.044(0.031,0.057)	0.007	1.01E-10	0	16,635
rs843991	3/187999122	T/C	0.05(0.038,0.062)	0.006	1.94E-16	0.34	16,635
rs3917110	3/188001346	G/C	-0.045(-0.058,-0.032)	0.007	4.11E-11	0	16,669
rs3917109	3/188001500	C/A	0.045(0.03,0.06)	0.008	6.12E-09	0.07	12,352
rs16861184	3/188003171	T/C	-0.045(-0.058,-0.032)	0.007	3.60E-11	0	16,669
rs710450	3/188005327	C/A	-0.05(-0.064,-0.037)	0.007	1.70E-13	0.33	12,333
rs2293243	3/188005431	T/A	-0.046(-0.059,-0.033)	0.007	2.21E-11	0	16,662
rs266717	3/188013178	T/C	0.039(0.028,0.051)	0.006	9.30E-11	0.76	16,680
rs6810075	3/188031259	T/C	0.059(0.046,0.071)	0.006	5.89E-20	0.28	16,477
rs10937273	3/188032389	G/A	-0.037(-0.049,-0.025)	0.006	1.11E-09	0.53	16,684
rs1648707	3/188034405	C/A	-0.054(-0.066,-0.042)	0.006	1.49E-18	0.30	16,683
rs822387	3/188038731	T/C	-0.139(-0.161,-0.117)	0.011	1.77E-33	0.61	16,575
rs17300539	3/188042154	G/A	-0.178(-0.203,-0.153)	0.013	8.37E-42	0.54	16,583

rs266729	3/188042168	G/C	-0.048(-0.061,-0.035)	0.007	3.82E-12	0.17	16,531
rs182052	3/188043476	G/A	0.054(0.042,0.066)	0.006	2.09E-18	0.33	16,643
rs16861209	3/188045808	C/A	-0.188(-0.214,-0.162)	0.013	4.65E-43	0.53	16,537
rs16861210	3/188049192	G/A	-0.172(-0.197,-0.147)	0.013	2.13E-39	0.44	16,473
rs17366568	3/188053147	G/A	0.149(0.126,0.172)	0.012	4.84E-36	0.83	13,782
rs1501299	3/188053817	T/G	0.074(0.059,0.089)	0.008	7.48E-22	0.70	12,125
rs3774261	3/188054253	G/A	-0.07(-0.082,-0.058)	0.006	4.89E-30	0.43	16,419
rs6773957	3/188056399	G/A	-0.07(-0.082,-0.059)	0.006	4.03E-30	0.43	16,419
rs1063538	3/188056877	T/C	0.065(0.051,0.079)	0.007	1.20E-19	0.40	12,101
rs7639352	3/188061168	T/C	0.077(0.064,0.09)	0.006	1.11E-31	0.68	16,665
rs6444175	3/188062438	G/A	-0.077(-0.09,-0.065)	0.006	8.25E-32	0.68	16,685
rs7615090	3/188073697	T/G	0.066(0.044,0.088)	0.011	4.96E-09	0.52	12,681
rs2954018	8/126546335	T/C	0.039(0.026,0.053)	0.007	1.70E-08	0	13,915
rs4871603	8/126549549	T/C	0.038(0.025,0.05)	0.006	9.34E-09	0	13,905
rs2980880	8/126550154	G/A	-0.039(-0.052,-0.026)	0.007	1.28E-08	0	13,924
rs2980879	8/126550657	T/A	0.039(0.026,0.052)	0.007	1.28E-08	0	13,924
rs2980878	8/126550709	G/C	-0.039(-0.052,-0.026)	0.007	1.13E-08	0	13,914
rs2980876	8/126550876	T/C	0.038(0.025,0.051)	0.007	2.07E-08	0	13,918
rs2954025	8/126553645	T/C	0.038(0.025,0.051)	0.006	6.48E-09	0	13,910
rs2954026	8/126553708	T/G	-0.039(-0.052,-0.026)	0.007	1.44E-08	0	13,914
rs2980862	8/126553820	G/C	-0.039(-0.052,-0.025)	0.007	2.40E-08	0	13,915
rs7828113	8/126554649	G/A	0.038(0.026,0.051)	0.006	5.70E-09	0	13,910
rs7846466	8/126554713	T/C	-0.039(-0.052,-0.026)	0.007	1.49E-08	0	13,914
rs2980867	8/126556873	T/G	-0.038(-0.052,-0.025)	0.007	4.22E-08	0	13,885
rs2954030	8/126560486	T/C	-0.037(-0.05,-0.024)	0.007	2.60E-08	0	13,872
rs2954032	8/126562574	G/A	0.04(0.027,0.053)	0.007	9.45E-09	0	13,875
rs2954033	8/126562928	G/A	0.04(0.027,0.053)	0.007	9.79E-09	0	13,874
rs2980859	8/126568611	G/C	0.041(0.027,0.054)	0.007	7.72E-09	0	13,834
rs2454722	12/121737171	G/A	0.043(0.028,0.058)	0.008	2.18E-08	0.11	16,683
rs601339	12/121740696	G/A	0.044(0.029,0.059)	0.008	1.24E-08	0.14	16,663
rs4759361	12/121744233	T/A	-0.044(-0.059,-0.029)	0.008	1.48E-08	0.17	16,653
rs509548	12/121747808	T/A	-0.044(-0.059,-0.029)	0.008	1.55E-08	0.17	16,652
rs2927328	16/80067223	T/C	-0.036(-0.049,-0.024)	0.006	1.56E-08	0.48	16,466
rs1471379	16/80068200	T/C	-0.036(-0.048,-0.025)	0.006	2.60E-09	0.55	16,599
rs1966957	16/80068580	G/C	-0.036(-0.048,-0.024)	0.006	2.58E-09	0.53	16,675
rs2927327	16/80069035	T/A	-0.036(-0.048,-0.024)	0.006	2.63E-09	0.53	16,674
rs2966079	16/80069307	T/C	0.036(0.024,0.047)	0.006	3.76E-09	0.58	16,521
rs1471152	16/80070137	T/G	-0.036(-0.047,-0.024)	0.006	3.24E-09	0.51	16,666
rs2927324	16/80070322	T/C	0.036(0.024,0.047)	0.006	3.46E-09	0.58	16,522
rs2927323	16/80070449	G/A	-0.039(-0.052,-0.027)	0.006	8.61E-10	0.52	16,647
rs2927322	16/80072006	G/A	0.045(0.032,0.058)	0.007	4.78E-11	0.50	16,354
rs2966085	16/80074135	G/A	0.037(0.025,0.049)	0.006	2.67E-09	0.45	16,388
rs2317241	16/80077267	G/A	0.04(0.028,0.053)	0.006	9.09E-10	0.36	15,894
rs12443634	16/80081775	C/A	0.05(0.037,0.063)	0.007	2.80E-13	0.06	16,493
rs2925979	16/80092291	T/C	-0.051(-0.063,-0.038)	0.006	1.59E-14	0	16,683
rs2966093	16/80096121	G/A	-0.047(-0.059,-0.034)	0.006	2.89E-13	0	16,499
rs2966094	16/80096138	C/A	0.048(0.035,0.06)	0.006	1.87E-13	0.09	16,499
rs2927307	16/80101333	G/A	-0.043(-0.055,-0.031)	0.006	1.26E-12	0.23	16,626
rs2966095	16/80106959	G/A	0.046(0.033,0.058)	0.006	7.96E-13	0.01	16,536
rs2966097	16/80107209	T/C	0.045(0.032,0.058)	0.007	1.78E-11	0.11	15,714
rs8047711	16/81225172	G/A	0.146(0.107,0.185)	0.020	6.77E-13	0.50	13,382
rs16957913	16/81227750	T/C	0.132(0.094,0.17)	0.020	3.06E-11	0.57	13,377
rs12599599	16/81228040	G/A	0.132(0.094,0.17)	0.020	3.46E-11	0.56	13,377
rs731839	19/38590905	G/A	-0.036(-0.048,-0.023)	0.006	3.65E-08	0.34	16,504
