# Supplementary material for: Novel Loci for Adiponectin Levels and Their Influence on Type 2 Diabetes and Metabolic Traits: A Multi-Ethnic Meta-Analysis of 45,891 Individuals
Source: PLoS Genet. 2012 Mar 29;8(3):e1002607. doi: 10.1371/journal.pgen.1002607 (PMC3315470; doi:10.1371/journal.pgen.1002607)
Supplement: Table S6 — SNPs associated with adiponectin at genome-wide significant levels (p<5×10^−8^) using fixed-effect models in men only in Euopean populations. (PDF) [file pgen.1002607.s009.pdf]

Table S6. **SNPs associated with adiponectin at genome-wide significant levels ($p < 5 \times 10^{-8}$) using fixed-effect models in men only in Euopean populations**.

SNPs	Chr/Position	EA/NEA	Beta (95 CI %)	SE	pvalue	I2	n
rs2062632	3/187943875	T/C	0.054(0.037,0.071)	0.009	1.33E-09	0.44	12,662
rs822355	3/187962817	T/C	-0.052(-0.068,-0.035)	0.008	1.10E-09	0.34	11,848
rs822354	3/187962900	G/A	-0.053(-0.068,-0.038)	0.008	9.46E-12	0.29	12,643
rs266733	3/187976007	T/G	0.052(0.038,0.065)	0.007	2.17E-13	0	12,657
rs185554	3/187977116	G/A	0.057(0.044,0.071)	0.007	3.41E-16	0	12,662
rs266719	3/187984342	T/C	0.075(0.059,0.091)	0.008	1.62E-19	0.24	12,661
rs3900626	3/187984594	T/C	-0.052(-0.07,-0.035)	0.009	1.23E-08	0.19	12,662
rs1426810	3/187986129	G/A	0.057(0.044,0.07)	0.007	1.45E-16	0	12,662
rs1354091	3/187988594	T/G	0.053(0.039,0.068)	0.007	1.66E-12	0	12,661
rs2066500	3/187990616	T/C	-0.052(-0.067,-0.038)	0.007	3.01E-12	0	12,662
rs266759	3/187991006	T/C	-0.054(-0.067,-0.041)	0.007	1.37E-15	0	12,661
rs266756	3/187991268	G/C	0.076(0.06,0.092)	0.008	7.12E-20	0.23	12,662
rs266754	3/187991660	T/C	-0.054(-0.067,-0.041)	0.007	2.04E-15	0	12,662
rs187868	3/187992211	G/A	0.054(0.041,0.067)	0.007	1.16E-15	0	12,661
rs3917117	3/187998538	G/A	0.054(0.039,0.069)	0.008	2.89E-12	0	12,662
rs843991	3/187999122	T/C	0.057(0.044,0.07)	0.007	6.49E-17	0	12,661
rs3917110	3/188001346	G/C	-0.055(-0.07,-0.04)	0.007	6.82E-13	0	12,662
rs16861184	3/188003171	T/C	-0.055(-0.07,-0.04)	0.008	9.64E-13	0	12,661
rs2293243	3/188005431	T/A	-0.055(-0.07,-0.04)	0.008	1.24E-12	0	12,661
rs6810075	3/188031259	T/C	0.077(0.063,0.091)	0.007	6.72E-26	0	12,661
rs10937273	3/188032389	G/A	-0.044(-0.057,-0.031)	0.007	1.52E-10	0.47	12,661
rs1648707	3/188034405	C/A	-0.074(-0.087,-0.061)	0.007	4.82E-26	0	12,662
rs822387	3/188038731	T/C	-0.161(-0.187,-0.135)	0.013	8.73E-33	0.52	12,660
rs266729	3/188042168	G/C	-0.076(-0.091,-0.061)	0.008	3.73E-22	0	12,661
rs182052	3/188043476	G/A	0.076(0.063,0.09)	0.007	1.98E-27	0	12,661
rs16861209	3/188045808	C/A	-0.199(-0.228,-0.17)	0.015	1.92E-39	0.35	12,661
rs16861210	3/188049192	G/A	-0.192(-0.22,-0.163)	0.014	1.24E-38	0.25	12,662
rs12495941	3/188050874	T/G	0.048(0.032,0.065)	0.008	1.83E-08	0.39	12,662
rs7649121	3/188051479	T/A	-0.085(-0.107,-0.063)	0.011	6.23E-14	0.28	11,601
rs3774261	3/188054253	G/A	-0.069(-0.082,-0.056)	0.007	1.41E-23	0.36	12,662
rs6773957	3/188056399	G/A	-0.069(-0.082,-0.056)	0.007	1.38E-23	0.36	12,661
rs7639352	3/188061168	T/C	0.067(0.053,0.082)	0.007	2.28E-19	0	12,662
rs6444175	3/188062438	G/A	-0.067(-0.081,-0.052)	0.007	6.69E-19	0	12,661
rs8047711	16/81225172	G/A	0.21(0.165,0.254)	0.023	1.38E-19	0.44	11,084
rs12922394	16/81229828	T/C	-0.132(-0.165,-0.098)	0.017	3.58E-14	0.08	11,083
rs731839	19/38590905	G/A	-0.04(-0.054,-0.026)	0.007	3.76E-08	0.11	12,662
