# Supplementary material for: Novel Loci for Adiponectin Levels and Their Influence on Type 2 Diabetes and Metabolic Traits: A Multi-Ethnic Meta-Analysis of 45,891 Individuals
Source: PLoS Genet. 2012 Mar 29;8(3):e1002607. doi: 10.1371/journal.pgen.1002607 (PMC3315470; doi:10.1371/journal.pgen.1002607)
Supplement: Table S7 — Association results of nominally significant SNPs with Type 2 Diabetes in the DIAGRAM+ Consortium. EA: Effect Allele, NEA: Non-Effect Allele. B) Association results of nominally significant SNPs with diabetes-related traits in the MAGIC Consortium. Fasting glucose and 2 h glucose in mmol/L; Insulin in pmol/L, EA: Effect Allele, NEA: Non-Effect Allele. C) Association results of nominally significant SNPs with diabetes-related traits in the GIANT and Body fat GWAS consortia. The beta expressed in inverse normally transformed BMI units (i.e. interpretable as SD or Z-score), shows the change in BMI per additional effect allele.,*Results that are statistically significant, accounting for the number of independent SNPs, are highlighted in bold., EA: Effect Allele, NEA: Non-Effect Allele, EA-Freq: Frequency of Effect Allele. D) Association results of nominally significant SNPs with lipid traits in the GLGC Consortium. For these traits the effect size is in SD units, based on standard error-weighted meta-analysis. *Results that are statistically significant, accounting for the number of independent SNPs are highlighted in bold., EA: Effect Allele, NEA: Non-Effect Allele, EA-Freq: Frequency of Effect Allele. (PDF) [file pgen.1002607.s010.pdf]

Table S7

A) Association results of nominally significant SNPs with Type 2 Diabetes in the DIAGRAM+ Consortium.

SNP	Chr/position	Closest Gene	EA/NEA	OR (95% CI)	P	N
rs1648707	3/188034413	*ADIPOQ*	C/A	1.045(1.002,1.089)	0.039	22,570
rs6444168	3/188035559	*ADIPOQ*	G/A	1.069(1.005,1.136)	0.034	21,520
rs266729	3/188042176	*ADIPOQ*	G/C	1.061(1.014,1.111)	0.011	22,570
rs182052	3/188043484	*ADIPOQ*	A/G	1.043(1,1.087)	0.048	22,570
rs7649121	3/188051487	*ADIPOQ*	T/A	1.122(1.041,1.209)	0.003	18,352
rs863750	12/123030324	*ZNF664*	T/C	1.051(1.008,1.096)	0.020	22,570
rs10773049	12/123031511	*ZNF664*	T/C	1.048(1.005,1.093)	0.030	22,570
rs825453	12/123033638	*ZNF664*	T/A	1.049(1.006,1.094)	0.026	22,570
rs2927322	16/80072006	*CMIP*	A/G	1.065(1.017,1.114)	0.007	22,570
rs12443634	16/80081775	*CMIP*	A/C	1.049(1.001,1.098)	0.045	21,198
rs2925979	16/80092291	*CMIP*	T/C	1.066(1.02,1.115)	0.005	21,198
rs2966093	16/80096121	*CMIP*	G/A	1.051(1.005,1.099)	0.030	21,198
rs2966094	16/80096138	*CMIP*	A/C	1.055(1.009,1.103)	0.019	21,198

EA: Effect Allele
NEA: Non-Effect Allele

Table S7

B) Association results of nominally significant SNPs with diabetes-related traits in the MAGIC Consortium.

SNP	Chr/position	Closest Gene	EA/NEA	Beta	SE	P	N	associated traits
rs1108842	3/52695120	*GNL3*	A/C	0.009	0.004	0.027	37,209	FI
rs2710323	3/52790945	*ITIH1*	T/C	0.009	0.004	0.019	37,197	FI
rs3617	3/52808845	*ITIH3*	A/C	-0.008	0.004	0.031	37,150	FI
rs4481150	3/52812833	*ITIH3*	T/C	0.008	0.004	0.042	37,801	FI
rs2535627	3/52820145	*ITIH4*	T/C	0.009	0.004	0.020	36,776	FI
rs2535627	3/52820145	*ITIH4*	T/C	0.008	0.004	0.041	35,477	HbA1C
rs2535627	3/52820145	*ITIH4*	T/C	0.008	0.004	0.041	35,477	Homa-IR
rs2071044	3/52822641	*ITIH4*	T/C	-0.008	0.004	0.037	37,802	FI
rs7617025	3/151538648	*TSC22D2*	A/G	0.013	0.006	0.040	46,186	FG
rs1972703	3/187946037	*KNG1*	A/G	0.061	0.027	0.024	15,234	2hr-Glu
rs266749	3/187954613	*KNG1*	T/C	0.009	0.004	0.037	44,713	FG
rs266742	3/187958539	*KNG1*	T/G	-0.008	0.004	0.048	45,972	FG
rs1501299	3/188053817	*ADIPOQ*	T/G	-0.016	0.007	0.022	14,398	FI
rs1501299	3/188053817	*ADIPOQ*	T/G	-0.015	0.008	0.048	13,363	HbA1C
rs1501299	3/188053817	*ADIPOQ*	T/G	-0.015	0.008	0.048	13,363	Homa-IR
rs12812995	12/20369141	*PDE3A*	C/G	-0.042	0.020	0.035	15,234	2hr-Glu
rs1444636	12/20384150	*PDE3A*	C/G	-0.046	0.020	0.018	15,234	2hr-Glu
rs10770643	12/20385101	*PDE3A*	A/G	0.047	0.020	0.016	15,234	2hr-Glu
rs7303397	12/20385638	*PDE3A*	A/G	0.049	0.019	0.012	15,234	2hr-Glu
rs7955516	12/20389303	*PDE3A*	A/C	0.045	0.020	0.021	15,234	2hr-Glu
rs2120757	12/20393267	*PDE3A*	T/C	0.045	0.019	0.021	15,234	2hr-Glu
rs2009084	12/20402460	*PDE3A*	T/C	0.047	0.020	0.016	15,234	2hr-Glu
rs11057405	12/121347850	*CLIP1*	A/G	0.017	0.008	0.033	32,319	FI
rs2454722	12/121737171	*GPR109A*	A/G	0.012	0.005	0.024	35,909	HbA1C
rs2454722	12/121737171	*GPR109A*	A/G	0.012	0.005	0.024	35,909	Homa-IR
rs2454722	12/121737171	*GPR109A*	A/G	0.011	0.005	0.031	37,208	FI
rs601339	12/121740696	*GPR109A*	A/G	0.012	0.005	0.028	37,032	HbA1C
rs601339	12/121740696	*GPR109A*	A/G	0.012	0.005	0.028	37,032	Homa-IR
rs601339	12/121740696	*GPR109A*	A/G	0.011	0.005	0.036	38,234	FI
rs4759361	12/121744233	*GPR109A*	A/T	-0.012	0.005	0.026	37,032	HbA1C
rs4759361	12/121744233	*GPR109A*	A/T	-0.012	0.005	0.026	37,032	Homa-IR
rs4759361	12/121744233	*GPR109A*	A/T	-0.011	0.005	0.034	38,234	FI
rs509548	12/121747808	*GPR109A*	A/T	-0.012	0.005	0.028	37,032	HbA1C
rs509548	12/121747808	*GPR109A*	A/T	-0.012	0.005	0.028	37,032	Homa-IR
rs509548	12/121747808	*GPR109A*	A/T	-0.011	0.005	0.036	38,234	FI
rs6488898	12/122769785	*ATP6V0A2*	A/G	-0.073	0.037	0.049	15,234	2hr-Glu
rs11057354	12/122833591	*DNAH10*	A/G	0.057	0.029	0.048	15,234	2hr-Glu
rs11057401	12/122993259	*CCDC92*	A/T	-0.008	0.004	0.044	46,170	FG
rs2178663	12/122999858	*CCDC92*	T/C	-0.008	0.004	0.049	46,185	FG
rs3867146	12/123003958	*CCDC92*	A/G	0.016	0.006	0.014	34,204	FI
rs3867146	12/123003958	*CCDC92*	A/G	0.014	0.007	0.036	32,954	HbA1C
rs3867146	12/123003958	*CCDC92*	A/G	0.014	0.007	0.036	32,954	Homa-IR
rs3867146	12/123003958	*CCDC92*	A/G	0.060	0.029	0.040	13,563	2hr-Glu
rs11831913	12/123012851	*CCDC92*	T/C	0.008	0.004	0.045	46,186	FG
rs4765148	12/123044590	*ZNF664*	T/G	-0.008	0.004	0.044	45,951	FG
rs2130382	12/123050596	*ZNF664*	C/G	0.008	0.004	0.041	46,184	FG
rs7307053	12/123060493	*ZNF664*	T/C	-0.008	0.004	0.040	46,186	FG
rs12824567	12/123061156	*ZNF664*	C/G	-0.008	0.004	0.039	46,186	FG

rs863750	12/123071397	*ZNF664*	T/C	0.009	0.004	0.028	37,187	FI
rs10773049	12/123072584	*ZNF664*	T/C	0.009	0.004	0.029	38,231	FI
rs825453	12/123074711	*ZNF664*	A/T	-0.009	0.004	0.031	37,801	FI
rs11057418	12/123074929	*ZNF664*	C/G	0.059	0.024	0.012	15,234	2hr-Glu
rs11057418	12/123074929	*ZNF664*	C/G	0.010	0.005	0.037	38,233	FI
rs2927328	16/80067223	*CMIP*	T/C	0.044	0.020	0.026	15,234	2hr-Glu
rs7193788	16/81213661	*CDH13*	A/G	-0.015	0.005	0.005	35,910	HbA1C
rs7193788	16/81213661	*CDH13*	A/G	-0.015	0.005	0.005	35,910	Homa-IR
rs7193788	16/81213661	*CDH13*	A/G	-0.013	0.005	0.013	37,208	FI
rs7193788	16/81213661	*CDH13*	A/G	-0.010	0.004	0.024	35,444	Homa-b
rs2163869	16/81223356	*CDH13*	A/G	0.009	0.004	0.032	42,602	FG
rs889140	19/38580840	*PEPD*	A/G	-0.009	0.004	0.031	37,210	FI
rs889140	19/38580840	*PEPD*	A/G	-0.009	0.004	0.033	35,912	HbA1C
rs889140	19/38580840	*PEPD*	A/G	-0.009	0.004	0.033	35,912	Homa-IR
rs889139	19/38581209	*PEPD*	A/G	0.009	0.004	0.038	36,975	HbA1C
rs889139	19/38581209	*PEPD*	A/G	0.009	0.004	0.038	36,975	Homa-IR
rs889139	19/38581209	*PEPD*	A/G	0.008	0.004	0.039	38,176	FI
rs731839	19/38590905	*PEPD*	A/G	-0.011	0.004	0.009	37,802	FI
rs731839	19/38590905	*PEPD*	A/G	-0.011	0.004	0.014	36,601	HbA1C
rs731839	19/38590905	*PEPD*	A/G	-0.011	0.004	0.014	36,601	Homa-IR
rs4805885	19/38597963	*PEPD*	T/C	0.012	0.004	0.002	37,802	FI
rs4805885	19/38597963	*PEPD*	T/C	0.012	0.004	0.003	36,601	HbA1C
rs4805885	19/38597963	*PEPD*	T/C	0.012	0.004	0.003	36,601	Homa-IR
rs8182584	19/38601550	*PEPD*	T/G	0.012	0.004	0.002	36,776	FI
rs8182584	19/38601550	*PEPD*	T/G	0.012	0.004	0.004	35,478	HbA1C
rs8182584	19/38601550	*PEPD*	T/G	0.012	0.004	0.004	35,478	Homa-IR
rs8182584	19/38601550	*PEPD*	T/G	0.007	0.004	0.041	35,012	Homa-b

Fasting glucose and 2h glucose in mmol/L; Insulin in pmol/L.
EA: Effect Allele
NEA: Non-Effect Allele

Table S7

C) Association results of nominally significant SNPs with diabetes-related traits in the GIANT and Body fat GWAS consortia.

SNP	Chr/position	Closest Gene	EA/NEA	Beta	SE	P	N	associated traits
**rs13081028**	**3/52530356**	***STAB1***	**A/G**	**-0.021**	**0.005**	**1.26E-05**	**77,167**	**WHR**
**rs9853056**	**3/52530997**	***STAB1***	**T/C**	**0.021**	**0.005**	**1.28E-05**	**77,167**	**WHR**
**rs4282054**	**3/52541105**	***NT5DC2***	**T/C**	**0.021**	**0.005**	**1.73E-05**	**77,167**	**WHR**
**rs2590838**	**3/52597126**	***PBRM1***	**A/G**	**-0.020**	**0.005**	**3.06E-05**	**77,167**	**WHR**
**rs2276824**	**3/52612526**	***PBRM1***	**C/G**	**0.019**	**0.005**	**2.80E-04**	**77,167**	**WHR**
**rs9879090**	**3/52623305**	***PBRM1***	**T/C**	**0.019**	**0.005**	**2.70E-04**	**77,167**	**WHR**
**rs13083798**	**3/52624788**	***PBRM1***	**A/G**	**0.020**	**0.005**	**1.95E-05**	**77,167**	**WHR**
**rs1108842**	**3/52695120**	***GNL3***	**A/C**	**0.021**	**0.005**	**1.68E-05**	**77,167**	**WHR**
**rs11235**	**3/52720127**	***NEK4***	**T/C**	**-0.019**	**0.005**	**3.10E-04**	**77,167**	**WHR**
**rs2710323**	**3/52790945**	***ITIH1***	**T/C**	**0.022**	**0.005**	**7.21E-06**	**77,167**	**WHR**
**rs3617**	**3/52808845**	***ITIH3***	**A/C**	**-0.020**	**0.005**	**4.59E-05**	**77,167**	**WHR**
rs3617	3/52808845	*ITIH3*	A/C	0.010	0.005	2.99E-02	123,745	BMI
**rs4481150**	**3/52812833**	***ITIH3***	**T/C**	**0.019**	**0.005**	**1.00E-04**	**77,167**	**WHR**
**rs2535627**	**3/52820145**	***ITIH4***	**T/C**	**0.019**	**0.005**	**8.22E-05**	**77,167**	**WHR**
**rs2071044**	**3/52822641**	***ITIH4***	**T/C**	**-0.019**	**0.005**	**1.10E-04**	**77,167**	**WHR**
rs11927941	3/187915740	*KNG1*	A/G	0.017	0.008	4.00E-02	34,832	fat%
rs3774291	3/187916165	*KNG1*	T/C	-0.017	0.008	4.20E-02	34,832	fat%
rs3774292	3/187916268	*KNG1*	A/T	0.016	0.008	3.70E-02	36,608	fat%
rs10440056	3/187916516	*KNG1*	T/C	-0.016	0.008	3.70E-02	36,608	fat%
rs3821815	3/187916956	*KNG1*	T/C	0.016	0.008	3.70E-02	36,619	fat%
rs1851665	3/187919092	*KNG1*	A/G	0.016	0.008	3.80E-02	36,610	fat%
rs1836860	3/187919298	*KNG1*	T/C	0.016	0.008	4.10E-02	36,611	fat%
rs1656925	3/187931817	*KNG1*	T/C	0.015	0.007	4.40E-02	36,625	fat%
rs1648700	3/187932610	*KNG1*	T/C	0.015	0.007	4.20E-02	36,613	fat%
rs1624569	3/187932763	*KNG1*	T/C	0.015	0.007	4.00E-02	36,601	fat%
rs710448	3/187935579	*KNG1*	A/G	0.015	0.007	4.40E-02	36,622	fat%
rs822363	3/187935685	*KNG1*	C/G	0.015	0.008	4.30E-02	36,622	fat%
rs2062632	3/187943875	*KNG1*	T/C	-0.013	0.006	3.90E-02	77,167	WHR
rs1426810	3/187986129	*EIF4A2*	A/G	0.016	0.008	3.10E-02	36,622	fat%
rs1354091	3/187988594	*EIF4A2*	T/G	-0.018	0.008	3.50E-02	36,611	fat%
rs2066500	3/187990616	*RFC4*	T/C	0.018	0.008	3.40E-02	36,607	fat%
rs3917117	3/187998538	*RFC4*	A/G	0.018	0.009	3.50E-02	36,597	fat%
rs3917110	3/188001346	*RFC4*	C/G	-0.017	0.008	4.20E-02	36,604	fat%
rs3917109	3/188001500	*RFC4*	A/C	0.028	0.012	1.70E-02	18,028	fat%
rs16861184	3/188003171	*RFC4*	T/C	0.017	0.008	4.10E-02	36,604	fat%
rs710450	3/188005327	*RFC4*	A/C	0.016	0.007	3.10E-02	77,167	WHR
rs2293243	3/188005431	*RFC4*	A/T	-0.017	0.008	4.40E-02	36,603	fat%
rs11045172	12/20361488	*PDE3A*	A/C	-0.015	0.007	1.83E-02	123,259	BMI
rs11057405	12/121347850	*CLIP1*	A/G	0.019	0.009	3.70E-02	77,167	WHR
rs11057405	12/121347850	*CLIP1*	A/G	-0.025	0.009	7.16E-03	122,590	BMI
rs2454722	12/121737171	*GPR109A*	A/G	0.018	0.006	4.00E-03	77,167	WHR
rs601339	12/121740696	*GPR109A*	A/G	0.018	0.006	3.60E-03	77,167	WHR
rs4759361	12/121744233	*GPR109A*	A/T	-0.018	0.006	3.70E-03	77,167	WHR
rs509548	12/121747808	*GPR109A*	A/T	-0.018	0.006	3.70E-03	77,167	WHR
rs6488898	12/122769785	*ATP6V0A2*	A/G	0.023	0.010	1.72E-02	119,083	BMI
rs11057354	12/122833591	*DNAH10*	A/G	0.021	0.007	8.40E-03	77,167	WHR
rs12317176	12/122970671	*DNAH10*	T/C	-0.015	0.005	2.24E-03	123,864	BMI
**rs12317176**	**12/122970671**	***DNAH10***	**T/C**	**0.023**	**0.005**	**5.31E-06**	**77,167**	**WHR**

rs12317176	12/122970671	*DNAH10*	T/C	-0.023	0.008	4.00E-03	36,607	fat%
rs7301953	12/122971824	*DNAH10*	A/G	0.014	0.005	7.03E-03	123,864	BMI
rs7301953	12/122971824	*DNAH10*	A/G	0.026	0.008	1.00E-03	36,593	fat%
**rs7301953**	**12/122971824**	***DNAH10***	**A/G**	**-0.022**	**0.005**	**1.50E-05**	**77,167**	**WHR**
**rs10846579**	**12/122973356**	***DNAH10***	**T/C**	**-0.023**	**0.005**	**4.61E-06**	**77,167**	**WHR**
rs10846579	12/122973356	*DNAH10*	T/C	0.015	0.005	2.20E-03	123,864	BMI
rs10846579	12/122973356	*DNAH10*	T/C	0.026	0.009	3.00E-03	29,051	fat%
**rs11057394**	**12/122973629**	***DNAH10***	**T/C**	**0.023**	**0.005**	**3.60E-06**	**77,167**	**WHR**
rs11057394	12/122973629	*DNAH10*	T/C	-0.015	0.005	2.17E-03	123,863	BMI
rs11057394	12/122973629	*DNAH10*	T/C	-0.024	0.008	3.00E-03	36,607	fat%
**rs12809125**	**12/122973944**	***DNAH10***	**A/G**	**0.023**	**0.005**	**4.66E-06**	**77,167**	**WHR**
rs12809125	12/122973944	*DNAH10*	A/G	-0.015	0.005	2.36E-03	123,864	BMI
rs12809125	12/122973944	*DNAH10*	A/G	-0.023	0.008	3.00E-03	36,608	fat%
rs7133378	12/122975455	*DNAH10*	A/G	0.016	0.005	1.70E-03	123,863	BMI
rs7133378	12/122975455	*DNAH10*	A/G	0.028	0.008	1.00E-03	34,831	fat%
**rs7133378**	**12/122975455**	***DNAH10***	**A/G**	**-0.022**	**0.005**	**2.38E-05**	**77,167**	**WHR**
rs9971695	12/122979444	*DNAH10*	A/G	0.015	0.005	2.94E-03	123,864	BMI
rs9971695	12/122979444	*DNAH10*	A/G	0.023	0.008	3.00E-03	36,608	fat%
**rs9971695**	**12/122979444**	***DNAH10***	**A/G**	**-0.023**	**0.005**	**6.74E-06**	**77,167**	**WHR**
**rs3802999**	**12/122980051**	***DNAH10***	**T/C**	**-0.023**	**0.005**	**5.87E-06**	**77,167**	**WHR**
rs3802999	12/122980051	*DNAH10*	T/C	0.023	0.008	4.00E-03	36,610	fat%
rs3802999	12/122980051	*DNAH10*	T/C	0.015	0.005	2.95E-03	123,864	BMI
rs4930721	12/122983842	*DNAH10*	T/C	0.013	0.005	8.68E-03	123,864	BMI
rs4930721	12/122983842	*DNAH10*	T/C	0.025	0.008	2.00E-03	36,600	fat%
**rs4930721**	**12/122983842**	***DNAH10***	**T/C**	**-0.022**	**0.005**	**2.32E-05**	**77,167**	**WHR**
rs12298484	12/122984627	*DNAH10*	T/C	0.023	0.008	4.00E-03	36,597	fat%
rs12298484	12/122984627	*DNAH10*	T/C	0.015	0.005	2.16E-03	123,789	BMI
**rs12298484**	**12/122984627**	***DNAH10***	**T/C**	**-0.023**	**0.005**	**7.22E-06**	**77,167**	**WHR**
rs11057396	12/122985015	*DNAH10*	A/C	-0.014	0.007	3.95E-02	67,240	BMI
rs11057397	12/122985681	*DNAH10*	T/C	0.022	0.008	5.00E-03	36,606	fat%
**rs11057397**	**12/122985681**	***DNAH10***	**T/C**	**-0.022**	**0.005**	**9.34E-06**	**77,167**	**WHR**
rs11057397	12/122985681	*DNAH10*	T/C	0.016	0.005	1.69E-03	123,864	BMI
rs9863	12/122987406	*CCDC92*	T/C	-0.015	0.005	2.84E-03	123,860	BMI
rs9863	12/122987406	*CCDC92*	T/C	-0.028	0.008	1.00E-03	34,831	fat%
**rs9863**	**12/122987406**	***CCDC92***	**T/C**	**0.021**	**0.005**	**4.36E-05**	**77,167**	**WHR**
rs4930723	12/122989553	*CCDC92*	C/G	0.022	0.008	5.00E-03	36,613	fat%
rs4930723	12/122989553	*CCDC92*	C/G	0.015	0.005	2.83E-03	123,864	BMI
**rs4930723**	**12/122989553**	***CCDC92***	**C/G**	**-0.022**	**0.005**	**8.89E-06**	**77,167**	**WHR**
rs11057401	12/122993259	*CCDC92*	A/T	0.024	0.008	3.00E-03	36,528	fat%
rs11057401	12/122993259	*CCDC92*	A/T	0.013	0.005	8.51E-03	123,779	BMI
**rs11057401**	**12/122993259**	***CCDC92***	**A/T**	**-0.021**	**0.005**	**3.77E-05**	**77,167**	**WHR**
rs4930726	12/122994284	*CCDC92*	T/C	-0.022	0.008	5.00E-03	36,610	fat%
**rs4930726**	**12/122994284**	***CCDC92***	**T/C**	**0.022**	**0.005**	**1.16E-05**	**77,167**	**WHR**
rs4930726	12/122994284	*CCDC92*	T/C	-0.015	0.005	2.87E-03	123,864	BMI
rs2178663	12/122999858	*CCDC92*	T/C	0.013	0.005	8.68E-03	123,864	BMI
**rs2178663**	**12/122999858**	***CCDC92***	**T/C**	**-0.021**	**0.005**	**3.39E-05**	**77,167**	**WHR**
rs2178663	12/122999858	*CCDC92*	T/C	0.024	0.008	2.00E-03	36,601	fat%
rs4405410	12/123001741	*CCDC92*	A/T	0.022	0.008	6.00E-03	36,610	fat%
rs4405410	12/123001741	*CCDC92*	A/T	0.015	0.005	3.00E-03	123,864	BMI
**rs4405410**	**12/123001741**	***CCDC92***	**A/T**	**-0.022**	**0.005**	**1.23E-05**	**77,167**	**WHR**
**rs7961449**	**12/123003168**	***CCDC92***	**A/T**	**-0.022**	**0.005**	**1.23E-05**	**77,167**	**WHR**
rs7961449	12/123003168	*CCDC92*	A/T	0.015	0.005	3.06E-03	123,864	BMI
rs7961449	12/123003168	*CCDC92*	A/T	0.022	0.008	6.00E-03	36,610	fat%

rs3867146	12/123003958	*CCDC92*	A/G	0.020	0.008	1.20E-02	77,167	WHR
rs7132655	12/123004926	*CCDC92*	C/G	0.015	0.005	3.41E-03	123,864	BMI
rs7132655	12/123004926	*CCDC92*	C/G	0.021	0.008	7.00E-03	36,608	fat%
**rs7132655**	**12/123004926**	***CCDC92***	**C/G**	**-0.022**	**0.005**	**1.34E-05**	**77,167**	**WHR**
rs4765219	12/123006063	*CCDC92*	A/C	0.015	0.005	3.04E-03	123,849	BMI
rs4765219	12/123006063	*CCDC92*	A/C	0.021	0.008	7.00E-03	36,606	fat%
**rs4765219**	**12/123006063**	***CCDC92***	**A/C**	**-0.022**	**0.005**	**1.53E-05**	**77,167**	**WHR**
**rs7958691**	**12/123006696**	***CCDC92***	**T/G**	**-0.022**	**0.005**	**1.40E-05**	**77,167**	**WHR**
rs7958691	12/123006696	*CCDC92*	T/G	0.025	0.009	3.00E-03	29,051	fat%
rs7958691	12/123006696	*CCDC92*	T/G	0.015	0.005	3.49E-03	123,864	BMI
**rs7305864**	**12/123007833**	***CCDC92***	**C/G**	**0.022**	**0.005**	**1.61E-05**	**77,167**	**WHR**
rs7305864	12/123007833	*CCDC92*	C/G	-0.022	0.008	5.00E-03	36,440	fat%
rs7305864	12/123007833	*CCDC92*	C/G	-0.014	0.005	3.88E-03	123,680	BMI
**rs6488913**	**12/123011522**	***CCDC92***	**C/G**	**-0.021**	**0.005**	**2.04E-05**	**77,167**	**WHR**
rs6488913	12/123011522	*CCDC92*	C/G	0.015	0.005	3.05E-03	123,864	BMI
rs6488913	12/123011522	*CCDC92*	C/G	0.022	0.008	6.00E-03	36,581	fat%
rs7312404	12/123012681	*CCDC92*	A/G	-0.025	0.011	1.90E-02	18,010	fat%
rs7312404	12/123012681	*CCDC92*	A/G	-0.015	0.007	3.26E-02	67,240	BMI
rs11831913	12/123012851	*CCDC92*	T/C	-0.013	0.005	1.01E-02	123,864	BMI
rs11831913	12/123012851	*CCDC92*	T/C	-0.024	0.008	2.00E-03	36,595	fat%
**rs11831913**	**12/123012851**	***CCDC92***	**T/C**	**0.021**	**0.005**	**5.38E-05**	**77,167**	**WHR**
rs6488914	12/123013794	*CCDC92*	C/G	0.024	0.008	2.00E-03	36,602	fat%
rs6488914	12/123013794	*CCDC92*	C/G	0.013	0.005	9.36E-03	123,841	BMI
**rs6488914**	**12/123013794**	***CCDC92***	**C/G**	**-0.021**	**0.005**	**4.56E-05**	**77,167**	**WHR**
rs7973683	12/123015176	*CCDC92*	A/C	0.015	0.005	3.06E-03	123,831	BMI
rs7973683	12/123015176	*CCDC92*	A/C	0.022	0.008	5.00E-03	36,584	fat%
**rs7973683**	**12/123015176**	***CCDC92***	**A/C**	**-0.022**	**0.005**	**1.98E-05**	**77,167**	**WHR**
rs4765127	12/123026120	*ZNF664*	T/G	0.023	0.008	4.00E-03	36,504	fat%
**rs4765127**	**12/123026120**	***ZNF664***	**T/G**	**-0.022**	**0.005**	**1.84E-05**	**77,167**	**WHR**
rs4765127	12/123026120	*ZNF664*	T/G	0.016	0.005	1.70E-03	123,737	BMI
rs12311114	12/123026656	*ZNF664*	A/C	0.026	0.008	1.00E-03	36,580	fat%
rs12311114	12/123026656	*ZNF664*	A/C	0.014	0.005	4.70E-03	123,847	BMI
**rs12311114**	**12/123026656**	***ZNF664***	**A/C**	**-0.021**	**0.005**	**3.38E-05**	**77,167**	**WHR**
rs4765528	12/123028207	*ZNF664*	A/T	0.014	0.005	5.41E-03	122,276	BMI
rs4765528	12/123028207	*ZNF664*	A/T	0.026	0.008	2.00E-03	34,681	fat%
**rs4765528**	**12/123028207**	***ZNF664***	**A/T**	**-0.022**	**0.005**	**2.74E-05**	**77,167**	**WHR**
rs11057408	12/123030789	*ZNF664*	T/G	0.022	0.008	5.00E-03	36,463	fat%
**rs11057408**	**12/123030789**	***ZNF664***	**T/G**	**-0.021**	**0.005**	**2.40E-05**	**77,167**	**WHR**
rs11057408	12/123030789	*ZNF664*	T/G	0.016	0.005	1.18E-03	123,709	BMI
rs7978610	12/123034525	*ZNF664*	C/G	0.016	0.005	1.06E-03	123,835	BMI
rs7978610	12/123034525	*ZNF664*	C/G	0.023	0.008	3.00E-03	36,551	fat%
**rs7978610**	**12/123034525**	***ZNF664***	**C/G**	**-0.022**	**0.005**	**1.04E-05**	**77,167**	**WHR**
rs952632	12/123035691	*ZNF664*	A/G	-0.016	0.005	1.07E-03	123,864	BMI
**rs952632**	**12/123035691**	***ZNF664***	**A/G**	**0.022**	**0.005**	**1.40E-05**	**77,167**	**WHR**
rs952632	12/123035691	*ZNF664*	A/G	-0.024	0.008	2.00E-03	36,613	fat%
rs11837287	12/123036012	*ZNF664*	T/C	0.016	0.005	1.00E-03	123,864	BMI
**rs11837287**	**12/123036012**	***ZNF664***	**T/C**	**-0.022**	**0.005**	**1.29E-05**	**77,167**	**WHR**
rs11837287	12/123036012	*ZNF664*	T/C	0.023	0.008	3.00E-03	36,619	fat%
rs7311969	12/123036286	*ZNF664*	T/C	-0.016	0.005	1.09E-03	123,792	BMI
**rs7311969**	**12/123036286**	***ZNF664***	**T/C**	**0.023**	**0.005**	**4.51E-06**	**77,167**	**WHR**
rs7311969	12/123036286	*ZNF664*	T/C	-0.023	0.008	3.00E-03	36,486	fat%
rs7307277	12/123041109	*ZNF664*	A/G	-0.016	0.005	9.66E-04	123,598	BMI
rs7307277	12/123041109	*ZNF664*	A/G	-0.023	0.008	3.00E-03	36,618	fat%
**rs7307277**	**12/123041109**	***ZNF664***	**A/G**	**0.022**	**0.005**	**1.26E-05**	**77,167**	**WHR**

rs7311233	12/123041893	*ZNF664*	A/G	-0.026	0.008	1.00E-03	36,599	fat%
rs7311233	12/123041893	*ZNF664*	A/G	-0.015	0.005	3.30E-03	123,829	BMI
**rs7311233**	**12/123041893**	***ZNF664***	**A/G**	**0.021**	**0.005**	**3.48E-05**	**77,167**	**WHR**
rs12809473	12/123042552	*ZNF664*	A/T	0.017	0.005	9.44E-04	123,864	BMI
rs12809473	12/123042552	*ZNF664*	A/T	0.023	0.008	3.00E-03	36,617	fat%
**rs12809473**	**12/123042552**	***ZNF664***	**A/T**	**-0.022**	**0.005**	**1.52E-05**	**77,167**	**WHR**
rs12827409	12/123042659	*ZNF664*	C/G	-0.023	0.008	3.00E-03	36,618	fat%
**rs12827409**	**12/123042659**	***ZNF664***	**C/G**	**0.022**	**0.005**	**1.52E-05**	**77,167**	**WHR**
rs12827409	12/123042659	*ZNF664*	C/G	-0.017	0.005	9.10E-04	123,864	BMI
rs7135314	12/123044519	*ZNF664*	A/T	-0.023	0.008	3.00E-03	36,618	fat%
**rs7135314**	**12/123044519**	***ZNF664***	**A/T**	**0.022**	**0.005**	**1.42E-05**	**77,167**	**WHR**
rs7135314	12/123044519	*ZNF664*	A/T	-0.016	0.005	9.61E-04	123,864	BMI
rs4765148	12/123044590	*ZNF664*	T/G	0.024	0.008	4.00E-03	34,803	fat%
rs4765148	12/123044590	*ZNF664*	T/G	0.015	0.005	4.09E-03	123,835	BMI
**rs4765148**	**12/123044590**	***ZNF664***	**T/G**	**-0.021**	**0.005**	**3.16E-05**	**77,167**	**WHR**
rs11057409	12/123045284	*ZNF664*	T/C	0.016	0.005	1.17E-03	123,784	BMI
**rs11057409**	**12/123045284**	***ZNF664***	**T/C**	**-0.022**	**0.005**	**1.21E-05**	**77,167**	**WHR**
rs11057409	12/123045284	*ZNF664*	T/C	0.024	0.008	3.00E-03	36,583	fat%
rs7975482	12/123047643	*ZNF664*	A/G	-0.024	0.008	3.00E-03	36,585	fat%
**rs7975482**	**12/123047643**	***ZNF664***	**A/G**	**0.022**	**0.005**	**1.38E-05**	**77,167**	**WHR**
rs7975482	12/123047643	*ZNF664*	A/G	-0.017	0.005	9.91E-04	123,837	BMI
**rs2130382**	**12/123050596**	***ZNF664***	**C/G**	**0.022**	**0.005**	**2.92E-05**	**77,167**	**WHR**
rs2130382	12/123050596	*ZNF664*	C/G	-0.015	0.005	3.56E-03	123,860	BMI
rs2130382	12/123050596	*ZNF664*	C/G	-0.025	0.008	2.00E-03	36,622	fat%
**rs12310367**	**12/123052631**	***ZNF664***	**A/G**	**0.022**	**0.005**	**1.72E-05**	**77,167**	**WHR**
rs12310367	12/123052631	*ZNF664*	A/G	-0.017	0.005	8.35E-04	123,859	BMI
rs12310367	12/123052631	*ZNF664*	A/G	-0.025	0.008	2.00E-03	36,612	fat%
**rs11057412**	**12/123055095**	***ZNF664***	**C/G**	**-0.022**	**0.005**	**1.71E-05**	**77,167**	**WHR**
rs11057412	12/123055095	*ZNF664*	C/G	0.017	0.005	7.97E-04	123,859	BMI
rs11057412	12/123055095	*ZNF664*	C/G	0.030	0.009	1.00E-03	29,056	fat%
rs1187415	12/123057482	*ZNF664*	C/G	-0.023	0.008	4.00E-03	36,551	fat%
**rs1187415**	**12/123057482**	***ZNF664***	**C/G**	**0.023**	**0.005**	**8.41E-06**	**77,167**	**WHR**
rs1187415	12/123057482	*ZNF664*	C/G	-0.017	0.005	7.99E-04	123,849	BMI
rs12303671	12/123058563	*ZNF664*	T/G	-0.024	0.008	3.00E-03	36,615	fat%
rs12303671	12/123058563	*ZNF664*	T/G	-0.017	0.005	8.55E-04	123,859	BMI
**rs12303671**	**12/123058563**	***ZNF664***	**T/G**	**0.022**	**0.005**	**1.92E-05**	**77,167**	**WHR**
**rs7307053**	**12/123060493**	***ZNF664***	**T/C**	**-0.021**	**0.005**	**4.58E-05**	**77,167**	**WHR**
rs7307053	12/123060493	*ZNF664*	T/C	0.026	0.008	2.00E-03	36,617	fat%
rs7307053	12/123060493	*ZNF664*	T/C	0.015	0.005	3.56E-03	123,851	BMI
rs12824567	12/123061156	*ZNF664*	C/G	0.015	0.005	3.40E-03	123,857	BMI
**rs12824567**	**12/123061156**	***ZNF664***	**C/G**	**-0.021**	**0.005**	**3.93E-05**	**77,167**	**WHR**
rs12824567	12/123061156	*ZNF664*	C/G	0.026	0.008	2.00E-03	36,623	fat%
**rs863750**	**12/123071397**	***ZNF664***	**T/C**	**0.020**	**0.005**	**7.67E-05**	**77,167**	**WHR**
**rs10773049**	**12/123072584**	***ZNF664***	**T/C**	**0.018**	**0.005**	**4.50E-04**	**77,167**	**WHR**
rs825453	12/123074711	*ZNF664*	A/T	-0.017	0.005	6.80E-04	77,167	WHR
rs11057418	12/123074929	*ZNF664*	C/G	0.020	0.006	1.10E-03	77,167	WHR
rs11057418	12/123074929	*ZNF664*	C/G	-0.017	0.006	7.50E-03	120,031	BMI
rs11057418	12/123074929	*ZNF664*	C/G	-0.031	0.010	2.00E-03	35,427	fat%
rs2927328	16/80067223	*CMIP*	T/C	-0.018	0.008	2.50E-02	34,832	fat%
rs1471379	16/80068200	*CMIP*	T/C	-0.017	0.008	2.10E-02	36,550	fat%
rs1966957	16/80068580	*CMIP*	C/G	0.018	0.008	1.90E-02	36,560	fat%
rs2927327	16/80069035	*CMIP*	A/T	0.017	0.008	2.00E-02	36,563	fat%
rs2966079	16/80069307	*CMIP*	T/C	0.015	0.007	4.20E-02	36,578	fat%
rs1471152	16/80070137	*CMIP*	T/G	-0.016	0.007	3.10E-02	36,561	fat%

rs2927323	16/80070449	*CMIP*	A/G	0.021	0.008	9.00E-03	36,603	fat%
rs2927323	16/80070449	*CMIP*	A/G	-0.011	0.005	3.50E-02	77,167	WHR
rs2927322	16/80072006	*CMIP*	A/G	0.012	0.005	2.70E-02	77,167	WHR
rs2927322	16/80072006	*CMIP*	A/G	-0.018	0.009	3.90E-02	34,831	fat%
rs2966085	16/80074135	*CMIP*	A/G	-0.018	0.008	1.90E-02	36,463	fat%
rs2317241	16/80077267	*CMIP*	A/G	-0.022	0.008	1.00E-02	34,831	fat%
rs12443634	16/80081775	*CMIP*	A/C	0.013	0.005	2.00E-02	77,167	WHR
rs12443634	16/80081775	*CMIP*	A/C	-0.020	0.009	2.40E-02	34,831	fat%
rs2925979	16/80092291	*CMIP*	T/C	0.013	0.005	1.50E-02	77,167	WHR
rs2925979	16/80092291	*CMIP*	T/C	-0.018	0.009	3.00E-02	34,832	fat%
rs2966093	16/80096121	*CMIP*	A/G	-0.011	0.005	3.10E-02	77,167	WHR
rs2966093	16/80096121	*CMIP*	A/G	0.017	0.008	3.00E-02	36,620	fat%
rs2966094	16/80096138	*CMIP*	A/C	0.012	0.005	2.40E-02	77,167	WHR
rs2966094	16/80096138	*CMIP*	A/C	-0.018	0.008	2.90E-02	36,562	fat%
rs2966095	16/80106959	*CMIP*	A/G	-0.019	0.008	2.20E-02	36,543	fat%
rs889140	19/38580840	*PEPD*	A/G	0.013	0.005	7.98E-03	123,845	BMI
rs889139	19/38581209	*PEPD*	A/G	-0.014	0.005	6.03E-03	123,846	BMI
rs731839	19/38590905	*PEPD*	A/G	0.014	0.005	5.20E-03	123,854	BMI
rs731839	19/38590905	*PEPD*	A/G	0.018	0.008	2.70E-02	34,832	fat%
rs4805885	19/38597963	*PEPD*	T/C	-0.016	0.005	1.65E-03	123,859	BMI
rs8182584	19/38601550	*PEPD*	T/G	-0.016	0.005	1.39E-03	123,858	BMI

The beta expressed in inverse normally transformed BMI units (i.e. interpretable as SD or Z-score), shows the change in BMI per additional effect allele.

***Results that are statistically significant, accounting for the number of independent SNPs, are highlighted in bold.**

EA: Effect Allele
NEA: Non-Effect Allele
EA-Freq: Frequency of Effect Allele

Table S7

D) Association results of nominally significant SNPs with lipid traits in the GLGC Consortium.

SNP	Chr/position	Closest Gene	EA/NEA	Beta	SE	P	N	associated traits
**rs13081028**	**3/52530356**	***STAB1***	**A/G**	**0.018**	**0.005**	**3.53E-04**	**99,900**	**HDL-C**
rs13081028	3/52530356	*STAB1*	A/G	-0.015	0.005	1.00E-03	96,598	TG
**rs9853056**	**3/52530997**	***STAB1***	**T/C**	**-0.018**	**0.005**	**3.08E-04**	**99,900**	**HDL-C**
rs9853056	3/52530997	*STAB1*	T/C	0.015	0.005	9.35E-04	96,598	TG
**rs4282054**	**3/52541105**	***NT5DC2***	**T/C**	**-0.018**	**0.005**	**3.38E-04**	**99,900**	**HDL-C**
rs4282054	3/52541105	*NT5DC2*	T/C	0.015	0.005	8.75E-04	96,598	TG
rs2590838	3/52597126	*PBRM1*	A/G	0.011	0.005	2.54E-02	99,900	HDL-C
rs2590838	3/52597126	*PBRM1*	A/G	-0.011	0.005	2.20E-02	96,598	TG
rs9879090	3/52623305	*PBRM1*	T/C	0.012	0.005	2.96E-02	92,418	TG
rs13083798	3/52624788	*PBRM1*	A/G	-0.010	0.005	2.82E-02	99,877	HDL-C
rs13083798	3/52624788	*PBRM1*	A/G	0.011	0.005	2.30E-02	96,575	TG
rs1108842	3/52695120	*GNL3*	A/C	-0.012	0.005	8.00E-03	99,900	HDL-C
rs1108842	3/52695120	*GNL3*	A/C	0.013	0.005	6.95E-03	96,598	TG
rs4481150	3/52812833	*ITIH3*	T/C	-0.009	0.005	4.50E-02	99,900	HDL-C
rs7636643	3/151532470	*TSC22D2*	A/T	0.021	0.009	7.54E-03	99,883	HDL-C
rs7636643	3/151532470	*TSC22D2*	A/T	-0.025	0.008	3.61E-03	96,582	TG
rs16862607	3/151533988	*TSC22D2*	A/G	0.021	0.009	8.71E-03	99,900	HDL-C
rs16862607	3/151533988	*TSC22D2*	A/G	-0.024	0.008	5.56E-03	96,598	TG
rs16862610	3/151535497	*TSC22D2*	T/C	0.020	0.009	1.32E-02	99,879	HDL-C
rs16862610	3/151535497	*TSC22D2*	T/C	-0.024	0.008	3.73E-03	96,577	TG
rs2060662	3/151536642	*TSC22D2*	C/G	0.020	0.009	1.20E-02	99,900	HDL-C
rs2060662	3/151536642	*TSC22D2*	C/G	-0.024	0.008	4.51E-03	96,598	TG
rs1597466	3/151538251	*TSC22D2*	T/G	-0.020	0.009	1.20E-02	99,900	HDL-C
rs1597466	3/151538251	*TSC22D2*	T/G	0.024	0.008	3.74E-03	96,598	TG
rs7616723	3/151538334	*TSC22D2*	A/G	-0.021	0.009	9.78E-03	99,900	HDL-C
rs7616723	3/151538334	*TSC22D2*	A/G	0.023	0.008	5.66E-03	96,598	TG
rs7617025	3/151538648	*TSC22D2*	A/G	-0.020	0.009	1.01E-02	99,900	HDL-C
rs7617025	3/151538648	*TSC22D2*	A/G	0.024	0.008	4.35E-03	96,598	TG
rs11924390	3/187915793	*KNG1*	T/C	0.010	0.005	3.91E-02	99,900	HDL-C
rs2062632	3/187943875	*KNG1*	T/C	0.012	0.006	4.39E-02	99,900	HDL-C
rs266760	3/187943910	*KNG1*	A/G	-0.024	0.011	2.68E-02	36,612	HDL-C
rs266760	3/187943910	*KNG1*	A/G	0.017	0.009	1.71E-02	33,198	TG
rs1354091	3/187988594	*EIF4A2*	T/G	0.007	0.006	4.47E-02	99,888	HDL-C
rs2066500	3/187990616	*RFC4*	T/C	-0.008	0.006	4.09E-02	99,891	HDL-C
rs3917110	3/188001346	*RFC4*	C/G	0.008	0.006	3.42E-02	99,900	HDL-C
rs16861184	3/188003171	*RFC4*	T/C	-0.008	0.006	4.10E-02	99,900	HDL-C
rs2293243	3/188005431	*RFC4*	A/T	0.009	0.006	3.06E-02	99,900	HDL-C
**rs2954018**	**8/126546335**	***TRIB1***	**T/C**	**0.040**	**0.005**	**1.93E-13**	**99,864**	**HDL-C**
**rs2954018**	**8/126546335**	***TRIB1***	**T/C**	**-0.038**	**0.006**	**2.05E-11**	**95,418**	**LDL-C**
**rs2954018**	**8/126546335**	***TRIB1***	**T/C**	**-0.042**	**0.006**	**3.60E-14**	**100,147**	**TC**
**rs2954018**	**8/126546335**	***TRIB1***	**T/C**	**-0.061**	**0.005**	**2.35E-31**	**96,561**	**TG**
**rs2980880**	**8/126550154**	***TRIB1***	**A/G**	**0.038**	**0.005**	**7.92E-13**	**99,900**	**HDL-C**
**rs2980880**	**8/126550154**	***TRIB1***	**A/G**	**-0.037**	**0.005**	**8.50E-12**	**95,454**	**LDL-C**
**rs2980880**	**8/126550154**	***TRIB1***	**A/G**	**-0.041**	**0.005**	**1.08E-14**	**100,184**	**TC**
**rs2980880**	**8/126550154**	***TRIB1***	**A/G**	**-0.058**	**0.005**	**3.01E-30**	**96,598**	**TG**
**rs2980879**	**8/126550657**	***TRIB1***	**A/T**	**-0.039**	**0.005**	**2.93E-13**	**99,900**	**HDL-C**
**rs2980879**	**8/126550657**	***TRIB1***	**A/T**	**0.038**	**0.005**	**3.12E-12**	**95,454**	**LDL-C**

**rs2980879**	**8/126550657**	***TRIB1***	**A/T**	**0.041**	**0.005**	**9.96E-15**	**100,184**	**TC**
**rs2980879**	**8/126550657**	***TRIB1***	**A/T**	**0.058**	**0.005**	**1.58E-29**	**96,598**	**TG**
**rs2980878**	**8/126550709**	***TRIB1***	**C/G**	**0.043**	**0.005**	**2.22E-15**	**96,908**	**HDL-C**
**rs2980878**	**8/126550709**	***TRIB1***	**C/G**	**-0.036**	**0.006**	**8.88E-11**	**92,503**	**LDL-C**
**rs2980878**	**8/126550709**	***TRIB1***	**C/G**	**-0.039**	**0.006**	**2.93E-12**	**97,148**	**TC**
**rs2980878**	**8/126550709**	***TRIB1***	**C/G**	**-0.058**	**0.005**	**1.16E-28**	**93,562**	**TG**
**rs2980876**	**8/126550876**	***TRIB1***	**T/C**	**0.038**	**0.005**	**1.24E-12**	**99,900**	**HDL-C**
**rs2980876**	**8/126550876**	***TRIB1***	**T/C**	**-0.037**	**0.005**	**8.75E-12**	**95,454**	**LDL-C**
**rs2980876**	**8/126550876**	***TRIB1***	**T/C**	**-0.041**	**0.005**	**1.25E-14**	**100,184**	**TC**
**rs2980876**	**8/126550876**	***TRIB1***	**T/C**	**-0.057**	**0.005**	**1.19E-29**	**96,598**	**TG**
**rs2954026**	**8/126553708**	***TRIB1***	**T/G**	**-0.041**	**0.005**	**2.20E-14**	**99,900**	**HDL-C**
**rs2954026**	**8/126553708**	***TRIB1***	**T/G**	**0.037**	**0.006**	**2.26E-11**	**95,454**	**LDL-C**
**rs2954026**	**8/126553708**	***TRIB1***	**T/G**	**0.041**	**0.006**	**4.37E-14**	**100,184**	**TC**
**rs2954026**	**8/126553708**	***TRIB1***	**T/G**	**0.060**	**0.005**	**1.48E-31**	**96,598**	**TG**
**rs7846466**	**8/126554713**	***TRIB1***	**T/C**	**-0.042**	**0.005**	**6.96E-15**	**96,908**	**HDL-C**
**rs7846466**	**8/126554713**	***TRIB1***	**T/C**	**0.036**	**0.006**	**1.66E-10**	**92,503**	**LDL-C**
**rs7846466**	**8/126554713**	***TRIB1***	**T/C**	**0.039**	**0.006**	**1.53E-12**	**97,148**	**TC**
**rs7846466**	**8/126554713**	***TRIB1***	**T/C**	**0.060**	**0.005**	**4.90E-30**	**93,562**	**TG**
**rs2954032**	**8/126562574**	***TRIB1***	**A/G**	**-0.041**	**0.005**	**3.93E-14**	**99,877**	**HDL-C**
**rs2954032**	**8/126562574**	***TRIB1***	**A/G**	**0.038**	**0.006**	**9.99E-12**	**95,433**	**LDL-C**
**rs2954032**	**8/126562574**	***TRIB1***	**A/G**	**0.041**	**0.006**	**4.70E-14**	**100,161**	**TC**
**rs2954032**	**8/126562574**	***TRIB1***	**A/G**	**0.061**	**0.005**	**6.18E-32**	**96,575**	**TG**
**rs2954033**	**8/126562928**	***TRIB1***	**A/G**	**-0.041**	**0.005**	**4.95E-14**	**99,900**	**HDL-C**
**rs2954033**	**8/126562928**	***TRIB1***	**A/G**	**0.037**	**0.006**	**3.34E-11**	**95,454**	**LDL-C**
**rs2954033**	**8/126562928**	***TRIB1***	**A/G**	**0.042**	**0.006**	**4.47E-14**	**100,184**	**TC**
**rs2954033**	**8/126562928**	***TRIB1***	**A/G**	**0.062**	**0.005**	**3.25E-32**	**96,598**	**TG**
**rs2980859**	**8/126568611**	***TRIB1***	**C/G**	**-0.042**	**0.005**	**2.33E-14**	**99,900**	**HDL-C**
**rs2980859**	**8/126568611**	***TRIB1***	**C/G**	**0.038**	**0.006**	**3.87E-11**	**95,454**	**LDL-C**
**rs2980859**	**8/126568611**	***TRIB1***	**C/G**	**0.040**	**0.006**	**6.34E-13**	**100,184**	**TC**
**rs2980859**	**8/126568611**	***TRIB1***	**C/G**	**0.060**	**0.005**	**5.98E-29**	**96,598**	**TG**
**rs2980888**	**8/126576490**	***TRIB1***	**T/C**	**-0.041**	**0.006**	**7.83E-12**	**99,900**	**HDL-C**
**rs2980888**	**8/126576490**	***TRIB1***	**T/C**	**0.042**	**0.006**	**6.97E-12**	**95,454**	**LDL-C**
**rs2980888**	**8/126576490**	***TRIB1***	**T/C**	**0.048**	**0.006**	**1.56E-15**	**100,184**	**TC**
**rs2980888**	**8/126576490**	***TRIB1***	**T/C**	**0.067**	**0.006**	**1.61E-31**	**96,598**	**TG**
**rs2954038**	**8/126576571**	***TRIB1***	**A/C**	**0.041**	**0.006**	**7.94E-12**	**99,900**	**HDL-C**
**rs2954038**	**8/126576571**	***TRIB1***	**A/C**	**-0.043**	**0.006**	**3.44E-12**	**95,454**	**LDL-C**
**rs2954038**	**8/126576571**	***TRIB1***	**A/C**	**-0.049**	**0.006**	**5.76E-16**	**100,184**	**TC**
**rs2954038**	**8/126576571**	***TRIB1***	**A/C**	**-0.067**	**0.006**	**1.01E-31**	**96,598**	**TG**
**rs11045172**	**12/20361488**	***PDE3A***	**A/C**	**-0.029**	**0.007**	**2.83E-06**	**98,285**	**HDL-C**
rs11045172	12/20361488	*PDE3A*	A/C	0.019	0.007	3.38E-03	94,984	TG
**rs12812995**	**12/20369141**	***PDE3A***	**C/G**	**0.019**	**0.005**	**2.72E-04**	**99,900**	**HDL-C**
rs12812995	12/20369141	*PDE3A*	C/G	-0.012	0.005	1.33E-02	96,598	TG
**rs1444636**	**12/20384150**	***PDE3A***	**C/G**	**0.019**	**0.005**	**1.46E-04**	**99,876**	**HDL-C**
rs1444636	12/20384150	*PDE3A*	C/G	-0.013	0.005	5.18E-03	96,574	TG
**rs10770643**	**12/20385101**	***PDE3A***	**A/G**	**-0.019**	**0.005**	**1.34E-04**	**99,900**	**HDL-C**
rs10770643	12/20385101	*PDE3A*	A/G	0.012	0.005	7.94E-03	96,598	TG
**rs7303397**	**12/20385638**	***PDE3A***	**A/G**	**-0.019**	**0.005**	**1.65E-04**	**99,900**	**HDL-C**
rs7303397	12/20385638	*PDE3A*	A/G	0.012	0.005	1.08E-02	96,598	TG
**rs7955516**	**12/20389303**	***PDE3A***	**A/C**	**-0.018**	**0.005**	**2.37E-04**	**99,890**	**HDL-C**
rs7955516	12/20389303	*PDE3A*	A/C	0.011	0.005	1.42E-02	96,588	TG
rs2120757	12/20393267	*PDE3A*	T/C	-0.016	0.005	6.60E-04	99,900	HDL-C
rs2120757	12/20393267	*PDE3A*	T/C	0.009	0.005	3.73E-02	96,598	TG
rs3943606	12/20399304	*PDE3A*	T/C	0.016	0.005	6.07E-04	99,900	HDL-C

rs3943606	12/20399304	*PDE3A*	T/C	-0.010	0.005	2.95E-02	96,598	TG
rs2009084	12/20402460	*PDE3A*	T/C	-0.017	0.005	5.21E-04	99,900	HDL-C
rs2009084	12/20402460	*PDE3A*	T/C	0.012	0.005	8.39E-03	96,598	TG
rs11057405	12/121347850	*CLIP1*	A/G	-0.026	0.010	3.24E-03	99,179	HDL-C
**rs2454722**	**12/121737171**	***GPR109A***	**A/G**	**-0.031**	**0.006**	**3.19E-07**	**99,179**	**HDL-C**
rs2454722	12/121737171	*GPR109A*	A/G	-0.017	0.007	8.58E-03	99,463	TC
rs2454722	12/121737171	*GPR109A*	A/G	0.018	0.006	5.30E-03	95,877	TG
**rs601339**	**12/121740696**	***GPR109A***	**A/G**	**-0.031**	**0.006**	**5.59E-07**	**99,179**	**HDL-C**
rs601339	12/121740696	*GPR109A*	A/G	-0.018	0.007	5.00E-03	99,463	TC
rs601339	12/121740696	*GPR109A*	A/G	0.017	0.006	8.26E-03	95,877	TG
**rs4759361**	**12/121744233**	***GPR109A***	**A/T**	**0.030**	**0.006**	**1.00E-06**	**99,179**	**HDL-C**
rs4759361	12/121744233	*GPR109A*	A/T	0.019	0.007	3.95E-03	99,463	TC
rs4759361	12/121744233	*GPR109A*	A/T	-0.015	0.006	2.43E-02	95,877	TG
**rs509548**	**12/121747808**	***GPR109A***	**A/T**	**0.031**	**0.006**	**3.40E-07**	**99,179**	**HDL-C**
rs509548	12/121747808	*GPR109A*	A/T	0.017	0.007	8.13E-03	99,463	TC
rs509548	12/121747808	*GPR109A*	A/T	-0.018	0.006	4.72E-03	95,877	TG
rs6488898	12/122769785	*ATP6V0A2*	A/G	0.024	0.010	5.10E-03	95,716	HDL-C
rs11057354	12/122833591	*DNAH10*	A/G	-0.024	0.008	2.37E-03	99,894	HDL-C
rs11057354	12/122833591	*DNAH10*	A/G	0.016	0.008	3.74E-02	96,592	TG
**rs12317176**	**12/122970671**	***DNAH10***	**T/C**	**-0.032**	**0.005**	**9.29E-10**	**99,900**	**HDL-C**
rs12317176	12/122970671	*DNAH10*	T/C	0.018	0.005	1.44E-03	95,454	LDL-C
rs12317176	12/122970671	*DNAH10*	T/C	0.015	0.005	8.26E-03	100,184	TC
**rs12317176**	**12/122970671**	***DNAH10***	**T/C**	**0.026**	**0.005**	**3.13E-07**	**96,598**	**TG**
**rs7301953**	**12/122971824**	***DNAH10***	**A/G**	**0.030**	**0.005**	**1.50E-08**	**99,900**	**HDL-C**
rs7301953	12/122971824	*DNAH10*	A/G	-0.018	0.006	1.19E-03	95,454	LDL-C
rs7301953	12/122971824	*DNAH10*	A/G	-0.017	0.005	4.10E-03	100,184	TC
**rs7301953**	**12/122971824**	***DNAH10***	**A/G**	**-0.026**	**0.005**	**6.12E-07**	**96,598**	**TG**
**rs10846579**	**12/122973356**	***DNAH10***	**T/C**	**0.032**	**0.005**	**9.57E-10**	**99,900**	**HDL-C**
rs10846579	12/122973356	*DNAH10*	T/C	-0.018	0.005	1.15E-03	95,454	LDL-C
rs10846579	12/122973356	*DNAH10*	T/C	-0.016	0.005	7.01E-03	100,184	TC
**rs10846579**	**12/122973356**	***DNAH10***	**T/C**	**-0.026**	**0.005**	**2.48E-07**	**96,598**	**TG**
**rs11057394**	**12/122973629**	***DNAH10***	**T/C**	**-0.031**	**0.005**	**1.54E-09**	**99,900**	**HDL-C**
rs11057394	12/122973629	*DNAH10*	T/C	0.018	0.005	8.30E-04	95,454	LDL-C
rs11057394	12/122973629	*DNAH10*	T/C	0.016	0.005	4.85E-03	100,184	TC
**rs11057394**	**12/122973629**	***DNAH10***	**T/C**	**0.026**	**0.005**	**1.79E-07**	**96,598**	**TG**
**rs12809125**	**12/122973944**	***DNAH10***	**A/G**	**-0.032**	**0.005**	**8.16E-10**	**99,900**	**HDL-C**
rs12809125	12/122973944	*DNAH10*	A/G	0.018	0.005	1.06E-03	95,454	LDL-C
rs12809125	12/122973944	*DNAH10*	A/G	0.016	0.005	6.00E-03	100,184	TC
**rs12809125**	**12/122973944**	***DNAH10***	**A/G**	**0.026**	**0.005**	**1.74E-07**	**96,598**	**TG**
**rs7133378**	**12/122975455**	***DNAH10***	**A/G**	**0.031**	**0.005**	**3.72E-09**	**99,900**	**HDL-C**
rs7133378	12/122975455	*DNAH10*	A/G	-0.018	0.006	1.91E-03	95,454	LDL-C
rs7133378	12/122975455	*DNAH10*	A/G	-0.015	0.006	1.14E-02	100,184	TC
**rs7133378**	**12/122975455**	***DNAH10***	**A/G**	**-0.023**	**0.005**	**5.49E-06**	**96,598**	**TG**
**rs9971695**	**12/122979444**	***DNAH10***	**A/G**	**0.032**	**0.005**	**6.73E-10**	**99,900**	**HDL-C**
rs9971695	12/122979444	*DNAH10*	A/G	-0.018	0.005	1.06E-03	95,454	LDL-C
rs9971695	12/122979444	*DNAH10*	A/G	-0.016	0.005	6.26E-03	100,184	TC
**rs9971695**	**12/122979444**	***DNAH10***	**A/G**	**-0.026**	**0.005**	**1.61E-07**	**96,598**	**TG**
**rs3802999**	**12/122980051**	***DNAH10***	**T/C**	**0.032**	**0.005**	**5.97E-10**	**99,900**	**HDL-C**
rs3802999	12/122980051	*DNAH10*	T/C	-0.018	0.005	1.39E-03	95,454	LDL-C
rs3802999	12/122980051	*DNAH10*	T/C	-0.015	0.005	8.26E-03	100,184	TC
**rs3802999**	**12/122980051**	***DNAH10***	**T/C**	**-0.026**	**0.005**	**1.77E-07**	**96,598**	**TG**
**rs4930721**	**12/122983842**	***DNAH10***	**T/C**	**0.030**	**0.005**	**2.07E-08**	**99,900**	**HDL-C**
rs4930721	12/122983842	*DNAH10*	T/C	-0.018	0.006	8.92E-04	95,454	LDL-C
rs4930721	12/122983842	*DNAH10*	T/C	-0.018	0.005	2.25E-03	100,184	TC

**rs4930721**	**12/122983842**	***DNAH10***	**T/C**	**-0.027**	**0.005**	**1.74E-07**	**96,598**	**TG**
**rs12298484**	**12/122984627**	***DNAH10***	**T/C**	**0.031**	**0.005**	**1.32E-09**	**99,846**	**HDL-C**
rs12298484	12/122984627	*DNAH10*	T/C	-0.018	0.005	1.22E-03	95,400	LDL-C
rs12298484	12/122984627	*DNAH10*	T/C	-0.016	0.005	5.48E-03	100,130	TC
**rs12298484**	**12/122984627**	***DNAH10***	**T/C**	**-0.027**	**0.005**	**7.52E-08**	**96,544**	**TG**
rs11057396	12/122985015	*DNAH10*	A/C	-0.020	0.009	7.07E-03	36,612	HDL-C
rs11057396	12/122985015	*DNAH10*	A/C	0.018	0.008	3.59E-02	33,198	TG
**rs11057397**	**12/122985681**	***DNAH10***	**T/C**	**0.031**	**0.005**	**7.47E-10**	**99,900**	**HDL-C**
rs11057397	12/122985681	*DNAH10*	T/C	-0.018	0.005	8.44E-04	95,454	LDL-C
rs11057397	12/122985681	*DNAH10*	T/C	-0.016	0.005	4.11E-03	100,184	TC
**rs11057397**	**12/122985681**	***DNAH10***	**T/C**	**-0.027**	**0.005**	**3.79E-08**	**96,598**	**TG**
**rs9863**	**12/122987406**	***CCDC92***	**T/C**	**-0.025**	**0.005**	**1.35E-06**	**99,900**	**HDL-C**
rs9863	12/122987406	*CCDC92*	T/C	0.017	0.006	1.57E-03	95,454	LDL-C
rs9863	12/122987406	*CCDC92*	T/C	0.016	0.006	4.01E-03	100,184	TC
**rs9863**	**12/122987406**	***CCDC92***	**T/C**	**0.023**	**0.005**	**2.94E-06**	**96,598**	**TG**
**rs4930723**	**12/122989553**	***CCDC92***	**C/G**	**0.032**	**0.005**	**5.02E-10**	**99,900**	**HDL-C**
rs4930723	12/122989553	*CCDC92*	C/G	-0.018	0.005	8.32E-04	95,454	LDL-C
rs4930723	12/122989553	*CCDC92*	C/G	-0.016	0.005	5.14E-03	100,184	TC
**rs4930723**	**12/122989553**	***CCDC92***	**C/G**	**-0.027**	**0.005**	**7.24E-08**	**96,598**	**TG**
**rs11057401**	**12/122993259**	***CCDC92***	**A/T**	**0.030**	**0.005**	**2.19E-08**	**96,797**	**HDL-C**
rs11057401	12/122993259	*CCDC92*	A/T	-0.018	0.006	1.20E-03	92,393	LDL-C
rs11057401	12/122993259	*CCDC92*	A/T	-0.017	0.005	2.75E-03	97,032	TC
**rs11057401**	**12/122993259**	***CCDC92***	**A/T**	**-0.027**	**0.005**	**1.53E-07**	**93,446**	**TG**
**rs4930726**	**12/122994284**	***CCDC92***	**T/C**	**-0.032**	**0.005**	**6.15E-10**	**99,900**	**HDL-C**
rs4930726	12/122994284	*CCDC92*	T/C	0.018	0.005	9.30E-04	95,454	LDL-C
rs4930726	12/122994284	*CCDC92*	T/C	0.016	0.005	5.49E-03	100,184	TC
**rs4930726**	**12/122994284**	***CCDC92***	**T/C**	**0.027**	**0.005**	**8.13E-08**	**96,598**	**TG**
**rs2178663**	**12/122999858**	***CCDC92***	**T/C**	**0.030**	**0.005**	**1.90E-08**	**96,908**	**HDL-C**
rs2178663	12/122999858	*CCDC92*	T/C	-0.018	0.006	1.35E-03	92,503	LDL-C
rs2178663	12/122999858	*CCDC92*	T/C	-0.017	0.005	3.27E-03	97,148	TC
**rs2178663**	**12/122999858**	***CCDC92***	**T/C**	**-0.027**	**0.005**	**1.59E-07**	**93,562**	**TG**
**rs4405410**	**12/123001741**	***CCDC92***	**A/T**	**0.032**	**0.005**	**6.28E-10**	**99,900**	**HDL-C**
rs4405410	12/123001741	*CCDC92*	A/T	-0.018	0.005	9.06E-04	95,454	LDL-C
rs4405410	12/123001741	*CCDC92*	A/T	-0.016	0.005	5.20E-03	100,184	TC
**rs4405410**	**12/123001741**	***CCDC92***	**A/T**	**-0.027**	**0.005**	**7.87E-08**	**96,598**	**TG**
**rs7961449**	**12/123003168**	***CCDC92***	**A/T**	**0.032**	**0.005**	**6.29E-10**	**99,900**	**HDL-C**
rs7961449	12/123003168	*CCDC92*	A/T	-0.018	0.005	9.64E-04	95,454	LDL-C
rs7961449	12/123003168	*CCDC92*	A/T	-0.016	0.005	5.59E-03	100,184	TC
**rs7961449**	**12/123003168**	***CCDC92***	**A/T**	**-0.027**	**0.005**	**6.45E-08**	**96,598**	**TG**
**rs7964945**	**12/123003621**	***CCDC92***	**A/T**	**0.022**	**0.007**	**4.53E-04**	**99,900**	**HDL-C**
rs7964945	12/123003621	*CCDC92*	A/T	-0.025	0.008	8.13E-04	95,454	LDL-C
rs7964945	12/123003621	*CCDC92*	A/T	-0.023	0.007	3.99E-03	100,184	TC
rs7964945	12/123003621	*CCDC92*	A/T	-0.022	0.007	1.89E-03	96,598	TG
**rs3867146**	**12/123003958**	***CCDC92***	**A/G**	**-0.031**	**0.008**	**1.64E-04**	**99,900**	**HDL-C**
rs3867146	12/123003958	*CCDC92*	A/G	0.024	0.008	3.07E-03	96,598	TG
**rs7132655**	**12/123004926**	***CCDC92***	**C/G**	**0.032**	**0.005**	**1.03E-09**	**96,908**	**HDL-C**
rs7132655	12/123004926	*CCDC92*	C/G	-0.018	0.005	1.26E-03	92,503	LDL-C
rs7132655	12/123004926	*CCDC92*	C/G	-0.016	0.005	6.64E-03	97,148	TC
**rs7132655**	**12/123004926**	***CCDC92***	**C/G**	**-0.027**	**0.005**	**1.06E-07**	**93,562**	**TG**
**rs4765219**	**12/123006063**	***CCDC92***	**A/C**	**0.033**	**0.005**	**4.36E-10**	**99,871**	**HDL-C**
rs4765219	12/123006063	*CCDC92*	A/C	-0.018	0.005	9.41E-04	95,427	LDL-C
rs4765219	12/123006063	*CCDC92*	A/C	-0.016	0.005	6.26E-03	100,151	TC
**rs4765219**	**12/123006063**	***CCDC92***	**A/C**	**-0.027**	**0.005**	**1.06E-07**	**96,565**	**TG**
**rs7958691**	**12/123006696**	***CCDC92***	**T/G**	**0.032**	**0.005**	**5.59E-10**	**99,900**	**HDL-C**

rs7958691	12/123006696	*CCDC92*	T/G	-0.018	0.005	7.51E-04	95,454	LDL-C
rs7958691	12/123006696	*CCDC92*	T/G	-0.016	0.005	5.45E-03	100,184	TC
**rs7958691**	**12/123006696**	***CCDC92***	**T/G**	**-0.027**	**0.005**	**8.78E-08**	**96,598**	**TG**
**rs7305864**	**12/123007833**	***CCDC92***	**C/G**	**-0.032**	**0.005**	**1.17E-09**	**99,686**	**HDL-C**
rs7305864	12/123007833	*CCDC92*	C/G	0.019	0.005	6.75E-04	95,243	LDL-C
rs7305864	12/123007833	*CCDC92*	C/G	0.016	0.005	4.16E-03	99,970	TC
**rs7305864**	**12/123007833**	***CCDC92***	**C/G**	**0.026**	**0.005**	**1.79E-07**	**96,384**	**TG**
**rs6488913**	**12/123011522**	***CCDC92***	**C/G**	**0.033**	**0.005**	**3.88E-10**	**99,900**	**HDL-C**
rs6488913	12/123011522	*CCDC92*	C/G	-0.018	0.005	7.62E-04	95,454	LDL-C
rs6488913	12/123011522	*CCDC92*	C/G	-0.016	0.005	5.61E-03	100,184	TC
**rs6488913**	**12/123011522**	***CCDC92***	**C/G**	**-0.027**	**0.005**	**7.33E-08**	**96,598**	**TG**
rs7312404	12/123012681	*CCDC92*	A/G	-0.019	0.009	1.03E-02	36,612	HDL-C
rs7312404	12/123012681	*CCDC92*	A/G	0.018	0.008	3.21E-02	33,198	TG
**rs11831913**	**12/123012851**	***CCDC92***	**T/C**	**-0.031**	**0.005**	**7.79E-09**	**99,900**	**HDL-C**
rs11831913	12/123012851	*CCDC92*	T/C	0.019	0.005	8.65E-04	95,454	LDL-C
rs11831913	12/123012851	*CCDC92*	T/C	0.017	0.005	2.80E-03	100,184	TC
**rs11831913**	**12/123012851**	***CCDC92***	**T/C**	**0.028**	**0.005**	**8.76E-08**	**96,598**	**TG**
**rs6488914**	**12/123013794**	***CCDC92***	**C/G**	**0.031**	**0.005**	**1.01E-08**	**99,873**	**HDL-C**
rs6488914	12/123013794	*CCDC92*	C/G	-0.019	0.006	7.33E-04	95,427	LDL-C
rs6488914	12/123013794	*CCDC92*	C/G	-0.018	0.005	2.26E-03	100,157	TC
**rs6488914**	**12/123013794**	***CCDC92***	**C/G**	**-0.028**	**0.005**	**1.04E-07**	**96,571**	**TG**
**rs7973683**	**12/123015176**	***CCDC92***	**A/C**	**0.032**	**0.005**	**3.46E-10**	**99,900**	**HDL-C**
rs7973683	12/123015176	*CCDC92*	A/C	-0.018	0.005	9.07E-04	95,454	LDL-C
rs7973683	12/123015176	*CCDC92*	A/C	-0.016	0.005	6.12E-03	100,184	TC
**rs7973683**	**12/123015176**	***CCDC92***	**A/C**	**-0.027**	**0.005**	**5.91E-08**	**96,598**	**TG**
**rs4765127**	**12/123026120**	***ZNF664***	**T/G**	**0.033**	**0.005**	**2.89E-10**	**99,787**	**HDL-C**
rs4765127	12/123026120	*ZNF664*	T/G	-0.018	0.005	9.36E-04	95,342	LDL-C
rs4765127	12/123026120	*ZNF664*	T/G	-0.016	0.005	5.07E-03	100,069	TC
**rs4765127**	**12/123026120**	***ZNF664***	**T/G**	**-0.028**	**0.005**	**1.80E-08**	**96,483**	**TG**
**rs12311114**	**12/123026656**	***ZNF664***	**A/C**	**0.031**	**0.005**	**8.49E-09**	**99,820**	**HDL-C**
rs12311114	12/123026656	*ZNF664*	A/C	-0.019	0.006	8.25E-04	95,377	LDL-C
rs12311114	12/123026656	*ZNF664*	A/C	-0.017	0.005	2.73E-03	100,103	TC
**rs12311114**	**12/123026656**	***ZNF664***	**A/C**	**-0.028**	**0.005**	**9.13E-08**	**96,517**	**TG**
**rs4765528**	**12/123028207**	***ZNF664***	**A/T**	**0.031**	**0.005**	**5.41E-09**	**99,711**	**HDL-C**
rs4765528	12/123028207	*ZNF664*	A/T	-0.019	0.005	6.23E-04	95,266	LDL-C
rs4765528	12/123028207	*ZNF664*	A/T	-0.017	0.005	2.67E-03	99,987	TC
**rs4765528**	**12/123028207**	***ZNF664***	**A/T**	**-0.028**	**0.005**	**5.17E-08**	**96,401**	**TG**
**rs11057408**	**12/123030789**	***ZNF664***	**T/G**	**0.033**	**0.005**	**5.93E-10**	**96,711**	**HDL-C**
rs11057408	12/123030789	*ZNF664*	T/G	-0.020	0.005	5.21E-04	92,308	LDL-C
rs11057408	12/123030789	*ZNF664*	T/G	-0.017	0.005	2.84E-03	96,943	TC
**rs11057408**	**12/123030789**	***ZNF664***	**T/G**	**-0.029**	**0.005**	**1.60E-08**	**93,357**	**TG**
**rs7978610**	**12/123034525**	***ZNF664***	**C/G**	**0.033**	**0.005**	**6.36E-10**	**96,878**	**HDL-C**
rs7978610	12/123034525	*ZNF664*	C/G	-0.019	0.006	7.33E-04	92,474	LDL-C
rs7978610	12/123034525	*ZNF664*	C/G	-0.017	0.005	3.49E-03	97,119	TC
**rs7978610**	**12/123034525**	***ZNF664***	**C/G**	**-0.029**	**0.005**	**1.43E-08**	**93,532**	**TG**
**rs952632**	**12/123035691**	***ZNF664***	**A/G**	**-0.032**	**0.005**	**8.59E-10**	**96,908**	**HDL-C**
rs952632	12/123035691	*ZNF664*	A/G	0.018	0.005	1.33E-03	92,503	LDL-C
rs952632	12/123035691	*ZNF664*	A/G	0.016	0.005	6.41E-03	97,148	TC
**rs952632**	**12/123035691**	***ZNF664***	**A/G**	**0.028**	**0.005**	**3.34E-08**	**93,562**	**TG**
**rs11837287**	**12/123036012**	***ZNF664***	**T/C**	**0.032**	**0.005**	**1.25E-09**	**96,908**	**HDL-C**
rs11837287	12/123036012	*ZNF664*	T/C	-0.019	0.006	8.90E-04	92,503	LDL-C
rs11837287	12/123036012	*ZNF664*	T/C	-0.017	0.005	4.90E-03	97,148	TC
**rs11837287**	**12/123036012**	***ZNF664***	**T/C**	**-0.028**	**0.005**	**4.46E-08**	**93,562**	**TG**

**rs7311969**	**12/123036286**	***ZNF664***	**T/C**	**-0.032**	**0.005**	**8.47E-10**	**99,796**	**HDL-C**
rs7311969	12/123036286	*ZNF664*	T/C	0.019	0.005	8.63E-04	95,350	LDL-C
rs7311969	12/123036286	*ZNF664*	T/C	0.016	0.005	4.63E-03	100,076	TC
**rs7311969**	**12/123036286**	***ZNF664***	**T/C**	**0.028**	**0.005**	**3.12E-08**	**96,490**	**TG**
**rs7307277**	**12/123041109**	***ZNF664***	**A/G**	**-0.032**	**0.005**	**1.09E-09**	**99,900**	**HDL-C**
rs7307277	12/123041109	*ZNF664*	A/G	0.019	0.005	7.43E-04	95,454	LDL-C
rs7307277	12/123041109	*ZNF664*	A/G	0.016	0.005	4.40E-03	100,184	TC
**rs7307277**	**12/123041109**	***ZNF664***	**A/G**	**0.027**	**0.005**	**4.57E-08**	**96,598**	**TG**
**rs7311233**	**12/123041893**	***ZNF664***	**A/G**	**-0.030**	**0.005**	**2.17E-08**	**99,874**	**HDL-C**
rs7311233	12/123041893	*ZNF664*	A/G	0.018	0.005	8.64E-04	95,428	LDL-C
rs7311233	12/123041893	*ZNF664*	A/G	0.018	0.005	2.24E-03	100,157	TC
**rs7311233**	**12/123041893**	***ZNF664***	**A/G**	**0.028**	**0.005**	**7.26E-08**	**96,571**	**TG**
**rs12809473**	**12/123042552**	***ZNF664***	**A/T**	**0.032**	**0.005**	**8.55E-10**	**99,900**	**HDL-C**
rs12809473	12/123042552	*ZNF664*	A/T	-0.019	0.005	7.89E-04	95,454	LDL-C
rs12809473	12/123042552	*ZNF664*	A/T	-0.016	0.005	4.69E-03	100,184	TC
**rs12809473**	**12/123042552**	***ZNF664***	**A/T**	**-0.028**	**0.005**	**3.56E-08**	**96,598**	**TG**
**rs12827409**	**12/123042659**	***ZNF664***	**C/G**	**-0.032**	**0.005**	**7.28E-10**	**99,900**	**HDL-C**
rs12827409	12/123042659	*ZNF664*	C/G	0.019	0.005	9.16E-04	95,454	LDL-C
rs12827409	12/123042659	*ZNF664*	C/G	0.016	0.005	5.17E-03	100,184	TC
**rs12827409**	**12/123042659**	***ZNF664***	**C/G**	**0.028**	**0.005**	**3.22E-08**	**96,598**	**TG**
**rs7135314**	**12/123044519**	***ZNF664***	**A/T**	**-0.032**	**0.005**	**1.06E-09**	**99,900**	**HDL-C**
rs7135314	12/123044519	*ZNF664*	A/T	0.019	0.005	5.90E-04	95,454	LDL-C
rs7135314	12/123044519	*ZNF664*	A/T	0.017	0.005	3.44E-03	100,184	TC
**rs7135314**	**12/123044519**	***ZNF664***	**A/T**	**0.028**	**0.005**	**3.27E-08**	**96,598**	**TG**
**rs4765148**	**12/123044590**	***ZNF664***	**T/G**	**0.031**	**0.005**	**1.54E-08**	**99,857**	**HDL-C**
rs4765148	12/123044590	*ZNF664*	T/G	-0.019	0.006	6.18E-04	95,412	LDL-C
rs4765148	12/123044590	*ZNF664*	T/G	-0.018	0.005	1.49E-03	100,140	TC
**rs4765148**	**12/123044590**	***ZNF664***	**T/G**	**-0.029**	**0.005**	**3.41E-08**	**96,554**	**TG**
**rs11057409**	**12/123045284**	***ZNF664***	**T/C**	**0.032**	**0.005**	**8.24E-10**	**99,851**	**HDL-C**
rs11057409	12/123045284	*ZNF664*	T/C	-0.019	0.005	7.96E-04	95,405	LDL-C
rs11057409	12/123045284	*ZNF664*	T/C	-0.017	0.005	3.92E-03	100,134	TC
**rs11057409**	**12/123045284**	***ZNF664***	**T/C**	**-0.028**	**0.005**	**2.23E-08**	**96,548**	**TG**
**rs7975482**	**12/123047643**	***ZNF664***	**A/G**	**-0.032**	**0.005**	**1.04E-09**	**99,875**	**HDL-C**
rs7975482	12/123047643	*ZNF664*	A/G	0.019	0.005	9.15E-04	95,429	LDL-C
rs7975482	12/123047643	*ZNF664*	A/G	0.017	0.005	4.15E-03	100,158	TC
**rs7975482**	**12/123047643**	***ZNF664***	**A/G**	**0.028**	**0.005**	**2.42E-08**	**96,572**	**TG**
**rs2130382**	**12/123050596**	***ZNF664***	**C/G**	**-0.031**	**0.005**	**2.05E-08**	**99,900**	**HDL-C**
rs2130382	12/123050596	*ZNF664*	C/G	0.020	0.006	5.36E-04	95,454	LDL-C
rs2130382	12/123050596	*ZNF664*	C/G	0.018	0.005	1.62E-03	100,184	TC
**rs2130382**	**12/123050596**	***ZNF664***	**C/G**	**0.028**	**0.005**	**5.25E-08**	**96,598**	**TG**
**rs12310367**	**12/123052631**	***ZNF664***	**A/G**	**-0.033**	**0.005**	**3.12E-10**	**99,900**	**HDL-C**
rs12310367	12/123052631	*ZNF664*	A/G	0.019	0.006	7.37E-04	95,454	LDL-C
rs12310367	12/123052631	*ZNF664*	A/G	0.016	0.006	4.29E-03	100,184	TC
**rs12310367**	**12/123052631**	***ZNF664***	**A/G**	**0.028**	**0.005**	**1.21E-08**	**96,598**	**TG**
**rs11057412**	**12/123055095**	***ZNF664***	**C/G**	**0.032**	**0.005**	**4.98E-10**	**99,900**	**HDL-C**
rs11057412	12/123055095	*ZNF664*	C/G	-0.018	0.006	1.02E-03	95,454	LDL-C
rs11057412	12/123055095	*ZNF664*	C/G	-0.016	0.006	5.81E-03	100,184	TC
**rs11057412**	**12/123055095**	***ZNF664***	**C/G**	**-0.028**	**0.005**	**2.32E-08**	**96,598**	**TG**
**rs1187415**	**12/123057482**	***ZNF664***	**C/G**	**-0.032**	**0.005**	**1.33E-09**	**96,870**	**HDL-C**
rs1187415	12/123057482	*ZNF664*	C/G	0.018	0.006	1.33E-03	92,468	LDL-C
rs1187415	12/123057482	*ZNF664*	C/G	0.016	0.006	4.66E-03	97,109	TC
**rs1187415**	**12/123057482**	***ZNF664***	**C/G**	**0.028**	**0.005**	**1.90E-08**	**93,523**	**TG**
**rs12303671**	**12/123058563**	***ZNF664***	**T/G**	**-0.032**	**0.005**	**1.60E-09**	**96,908**	**HDL-C**
rs12303671	12/123058563	*ZNF664*	T/G	0.018	0.006	1.40E-03	92,503	LDL-C

rs12303671	12/123058563	*ZNF664*	T/G	0.016	0.006	5.72E-03	97,148	TC
**rs12303671**	**12/123058563**	***ZNF664***	**T/G**	**0.028**	**0.005**	**3.62E-08**	**93,562**	**TG**
**rs7307053**	**12/123060493**	***ZNF664***	**T/C**	**0.030**	**0.005**	**5.53E-08**	**96,899**	**HDL-C**
rs7307053	12/123060493	*ZNF664*	T/C	-0.018	0.006	1.94E-03	92,494	LDL-C
rs7307053	12/123060493	*ZNF664*	T/C	-0.017	0.006	3.60E-03	97,138	TC
**rs7307053**	**12/123060493**	***ZNF664***	**T/C**	**-0.027**	**0.005**	**1.01E-07**	**93,552**	**TG**
**rs12824567**	**12/123061156**	***ZNF664***	**C/G**	**0.030**	**0.005**	**1.47E-08**	**99,900**	**HDL-C**
rs12824567	12/123061156	*ZNF664*	C/G	-0.019	0.006	6.70E-04	95,454	LDL-C
rs12824567	12/123061156	*ZNF664*	C/G	-0.018	0.006	2.07E-03	100,184	TC
**rs12824567**	**12/123061156**	***ZNF664***	**C/G**	**-0.028**	**0.005**	**3.97E-08**	**96,598**	**TG**
**rs863750**	**12/123071397**	***ZNF664***	**T/C**	**-0.032**	**0.005**	**7.79E-10**	**99,889**	**HDL-C**
rs863750	12/123071397	*ZNF664*	T/C	0.014	0.005	2.79E-03	96,587	TG
**rs10773049**	**12/123072584**	***ZNF664***	**T/C**	**-0.030**	**0.005**	**7.32E-09**	**99,900**	**HDL-C**
rs10773049	12/123072584	*ZNF664*	T/C	-0.012	0.005	2.66E-02	100,184	TC
rs10773049	12/123072584	*ZNF664*	T/C	0.012	0.005	8.76E-03	96,598	TG
**rs825453**	**12/123074711**	***ZNF664***	**A/T**	**0.031**	**0.005**	**2.87E-09**	**99,900**	**HDL-C**
rs825453	12/123074711	*ZNF664*	A/T	0.011	0.005	2.72E-02	100,184	TC
rs825453	12/123074711	*ZNF664*	A/T	-0.013	0.005	5.15E-03	96,598	TG
**rs11057418**	**12/123074929**	***ZNF664***	**C/G**	**-0.027**	**0.007**	**5.19E-05**	**94,822**	**HDL-C**
**rs11057418**	**12/123074929**	***ZNF664***	**C/G**	**0.025**	**0.006**	**1.41E-04**	**91,518**	**TG**
**rs2927328**	**16/80067223**	***CMIP***	**T/C**	**-0.020**	**0.005**	**2.71E-05**	**98,409**	**HDL-C**
rs2927328	16/80067223	*CMIP*	T/C	0.017	0.005	8.01E-03	95,070	TG
**rs1471379**	**16/80068200**	***CMIP***	**T/C**	**-0.023**	**0.005**	**4.68E-06**	**96,852**	**HDL-C**
rs1471379	16/80068200	*CMIP*	T/C	0.012	0.005	4.48E-02	93,505	TG
**rs1966957**	**16/80068580**	***CMIP***	**C/G**	**0.023**	**0.005**	**2.67E-06**	**98,409**	**HDL-C**
rs1966957	16/80068580	*CMIP*	C/G	-0.013	0.005	2.52E-02	95,070	TG
**rs2927327**	**16/80069035**	***CMIP***	**A/T**	**0.023**	**0.005**	**3.33E-06**	**98,409**	**HDL-C**
rs2927327	16/80069035	*CMIP*	A/T	-0.012	0.005	2.85E-02	95,070	TG
**rs2966079**	**16/80069307**	***CMIP***	**T/C**	**0.020**	**0.005**	**7.92E-06**	**98,409**	**HDL-C**
**rs1471152**	**16/80070137**	***CMIP***	**T/G**	**-0.023**	**0.005**	**3.15E-06**	**98,313**	**HDL-C**
rs1471152	16/80070137	*CMIP*	T/G	0.012	0.005	2.70E-02	94,971	TG
**rs2927324**	**16/80070322**	***CMIP***	**T/C**	**0.021**	**0.005**	**4.79E-06**	**98,386**	**HDL-C**
**rs2927323**	**16/80070449**	***CMIP***	**A/G**	**0.022**	**0.005**	**9.20E-06**	**98,409**	**HDL-C**
rs2927323	16/80070449	*CMIP*	A/G	-0.017	0.005	3.33E-03	95,070	TG
**rs2927322**	**16/80072006**	***CMIP***	**A/G**	**-0.025**	**0.006**	**2.00E-06**	**98,409**	**HDL-C**
rs2927322	16/80072006	*CMIP*	A/G	0.017	0.005	1.01E-02	95,070	TG
**rs2966085**	**16/80074135**	***CMIP***	**A/G**	**-0.020**	**0.005**	**7.10E-06**	**98,256**	**HDL-C**
rs2966085	16/80074135	*CMIP*	A/G	0.015	0.005	1.07E-02	94,917	TG
**rs2317241**	**16/80077267**	***CMIP***	**A/G**	**-0.025**	**0.005**	**1.71E-06**	**98,409**	**HDL-C**
rs2317241	16/80077267	*CMIP*	A/G	0.018	0.005	1.27E-03	95,070	TG
**rs12443634**	**16/80081775**	***CMIP***	**A/C**	**-0.032**	**0.006**	**2.01E-09**	**98,409**	**HDL-C**
**rs12443634**	**16/80081775**	***CMIP***	**A/C**	**0.022**	**0.005**	**2.00E-04**	**95,070**	**TG**
**rs2925979**	**16/80092291**	***CMIP***	**T/C**	**-0.035**	**0.005**	**2.09E-11**	**98,409**	**HDL-C**
**rs2925979**	**16/80092291**	***CMIP***	**T/C**	**0.022**	**0.005**	**8.97E-05**	**95,070**	**TG**
**rs2966093**	**16/80096121**	***CMIP***	**A/G**	**0.023**	**0.005**	**1.02E-05**	**98,369**	**HDL-C**
rs2966093	16/80096121	*CMIP*	A/G	-0.012	0.005	3.74E-02	95,031	TG
**rs2966094**	**16/80096138**	***CMIP***	**A/C**	**-0.025**	**0.005**	**1.63E-06**	**98,409**	**HDL-C**
rs2966094	16/80096138	*CMIP*	A/C	0.014	0.005	2.12E-02	95,070	TG
**rs2927307**	**16/80101333**	***CMIP***	**A/G**	**0.017**	**0.005**	**1.09E-04**	**98,409**	**HDL-C**
**rs2966095**	**16/80106959**	***CMIP***	**A/G**	**-0.022**	**0.005**	**1.79E-05**	**98,409**	**HDL-C**
**rs2966097**	**16/80107209**	***CMIP***	**T/C**	**0.023**	**0.006**	**1.72E-05**	**88,953**	**HDL-C**
rs2966097	16/80107209	*CMIP*	T/C	-0.015	0.006	1.28E-02	85,615	TG
**rs889140**	**19/38580840**	***PEPD***	**A/G**	**0.020**	**0.005**	**1.71E-05**	**98,409**	**HDL-C**
rs889140	19/38580840	*PEPD*	A/G	-0.013	0.005	1.07E-02	95,070	TG

**rs889139**	**19/38581209**	***PEPD***	**A/G**	**-0.020**	**0.005**	**2.19E-05**	**98,386**	**HDL-C**
rs889139	19/38581209	*PEPD*	A/G	0.013	0.005	1.33E-02	95,047	TG
**rs731839**	**19/38590905**	***PEPD***	**A/G**	**0.025**	**0.005**	**1.09E-06**	**98,409**	**HDL-C**
**rs731839**	**19/38590905**	***PEPD***	**A/G**	**-0.020**	**0.005**	**2.15E-04**	**95,070**	**TG**
**rs4805885**	**19/38597963**	***PEPD***	**T/C**	**-0.025**	**0.005**	**6.81E-07**	**98,409**	**HDL-C**
rs4805885	19/38597963	*PEPD*	T/C	0.016	0.005	2.54E-03	95,070	TG
**rs8182584**	**19/38601550**	***PEPD***	**T/G**	**-0.025**	**0.005**	**3.19E-07**	**98,409**	**HDL-C**
rs8182584	19/38601550	*PEPD*	T/G	0.015	0.005	3.62E-03	95,070	TG

For these traits the effect size is in SD units, based on standard error-weighted meta-analysis. ***Results that are statistically significant, accounting for the number of independent SNPs are highlighted in bold.**

EA: Effect Allele

NEA: Non-Effect Allele

EA-Freq: Frequency of Effect Allele
