# Supplementary material for: Novel Loci for Adiponectin Levels and Their Influence on Type 2 Diabetes and Metabolic Traits: A Multi-Ethnic Meta-Analysis of 45,891 Individuals
Source: PLoS Genet. 2012 Mar 29;8(3):e1002607. doi: 10.1371/journal.pgen.1002607 (PMC3315470; doi:10.1371/journal.pgen.1002607)
Supplement: Text S1 — Supplemental data include description of study cohorts and funding. (DOCX) [file pgen.1002607.s011.docx]

**Supplemental Data**

**Novel Loci for Adiponectin Levels and their Influence on Type 2 Diabetes and Metabolic Traits: A Multi-Ethnic Meta-Analysis of 45,891 Individuals**

Supplemental data include description of study cohorts.

**Study cohorts**:

In this study, we included sixteen cohorts from the ADIPOGen Consortium in the discovery phase. An additional eleven independent cohorts were added for the replication studies. The research protocols of all studies were reviewed and approved by institutional ethics review committees at the involved institutes. All participants provided written informed consent.

**Study descriptions.**

1. **Discovery phase cohorts.**

**European cohorts.**

1. **Avon Longitudinal Study of Parents and their Children (ALSPAC).** The ALSPAC is a population-based birth cohort study, which started in early 1990’s in the county of Avon, U.K^1^. More than 14,000 mothers were enrolled during pregnancy in 1991 and 1992. Data was collected from the mothers during pregnancy, and their children have been followed at regular intervals during their childhood. The analysis was restricted to 2,692 samples with adiponectin measurements.
2. **Baltimore Longitudinal Study of Aging (BLSA).** The BLSA is an observational study that began in 1958 to trace the effects of aging on normal community-residing adults^2^. The study consists of volunteers recruited primarily from the Washington, DC, and Baltimore, MD, areas. In total, 597 samples out of 1,230 participants with available genome-wide data and adiponectin measurements were used in this study.
3. **Caucasian Cohort Lausannoise Study** **(CoLaus).** The CoLaus is a population-based study of the population registry of the city of Lausanne, Switzerland. Only individuals with four grandparents of European origin were included in this study. This study aimed to examine the epidemiological and genetic determinants of cardiovascular risk factors and metabolic syndrome. This study has been described in detail previously^3^. A total of 5,261 individuals with adiponectin measurements were included in the current analysis.
4. **Erasmus Rucphen Family (ERF).** The ERF is a family-based and genetically-isolated study cohort from the Rucphen region located in the southwest of the Netherlands and has been described in detail previously^4^. The selection of subjects in the ERF cohort was based on pedigree structure and not on health status. All ERF participants are descendants from 22 couples with at least 6 children born in the year 1850. Of those 1,817 individuals for whom phenotypic and genotypic information was available, are included in our meta-analysis.
5. **Framingham Heart Study** (**FHS).** The FHS was initiated in 1948 and is comprised of 5,209 participants from Framingham, MA (US), who have undergone examinations every other year to evaluate cardiovascular disease and related risk factors. The Offspring cohort was recruited in 1971 and includes 5,124 children of the Original cohort and the children’s spouses^5^. Participants from the Offspring cohort have attended exams roughly every four years. Adiponectin levels were measured using specimens collected at the 7th exam from the Offspring cohort. The current analysis includes 2,223 individuals with available phenotypic and genotypic information.
6. **Genetic Etiology of Metabolic Syndrome (GEMS).** The GEMS study is a case-control study for dyslipidemia, as previously described^6^. Our study was restricted to 1,780 samples with available genotypes and adiponectin measurements.
7. **Invecchaire in Chianti (InCHIANTI).** InCHIANTI has been described in detail previously^7; 8^. This is a population-based study of individuals living in the Chianti geographic area (Tuscany, Italy) that was initiated to understand the causes of walking difficulties in older individuals. 1,097 participants with available adiponectin and genotype data were included in the present analysis.
8. **The Collaborative Health Research in the Region of Augsburg (KORA F3).** This study comprises three population-representative surveys from Augsburg and surrounding counties of Southern Germany to describe time trends of classical cardiovascular risk factors^9^. All study participants underwent a standardized face-to-face interview by certified medical staff and a standardized medical examination including blood draw and anthropometric measurements. The 1,644 subjects for the KORA GWA analysis (the KORA S3/F3 500K study) were chosen from KORA F3. In total, 1,636 participants were included in this analysis.
9. **MICROS Study of Population Microisolates in South Tyrol.** The MICROS study has been described in detail previously.^10^ As part of the genomic healthcare program “GenNova,” an extensive survey was carried out during 2001–2003 in three villages of the Val Venosta (South Tyrol, Italy), on the populations of Stelvio, Vallelunga, and Martello. This survey aimed at characterizing the genetic epidemiology of Mendelian and complex diseases^11^.This analysis incorporates 1,194 participants with available adiponectin and genotypic data.
10. **TwinsUK Study.** TUK is a population-based sample of British twins, which is representative of the general United Kingdom population, and is has been extensively phenotyped for aging-related traits^12; 13^. They were genotyped at two stages as called TUK1 and TUK23 in our study. 968 individuals from TUK1 and 1,229 samples from TUK23 are included in this meta-analysis.
11. **Cardiovascular Health Study (CHS**). The CHS has been described in detail previously^14^. The CHS is a population-based cohort study of risk factors for coronary heart disease (CHD) and stroke in adults ≥65 years conducted across four field centers. The original cohort predominantly of European descent (n = 5,201 individuals) was recruited in 1989-1990 from random samples of the Medicare eligibility lists. Subsequently, an additional predominantly African-American cohort of 687 individuals was enrolled, resulting in a total sample of 5,888. This analysis includes 2,718 white individuals with genotype data and adiponectin measurements.
12. **Cardiovascular Risk in Young Finns (YFS).** This population-based follow-up study started in 1980 (http://med.utu.fi/cardio/youngfinnsstudy/). A subsequent 27-year-follow-up study was conducted in 2007 (ages 30-45 years) ^15^. 2,424 individuals with available adiponectin measurements were integrated in this analysis.
13. **Helsinki Birth Cohort Study (HBCS).** This study is a population-based birth cohort consisting of individuals born in the Helsinki area during the years1924-33 (n=7,086) and 1934-44 (n=8,760)^16^. This study combines the information from various Finnish national health-care registries. This analysis is limited to 1,723 individuals with available genotype and adiponectin data.
14. **Dietary, Lifestyle, and Genetic determinants of Obesity and Metabolic syndrome (DILGOM).** The DILGOM is a study about dietary, lifestyle, and genetic determinants of obesity and metabolic syndrome in a population-based cohort of men and women aged 25 to 44 years sampled from Finland as a part of FINRISK 2007 study^17^. Data from 687 individuals is included in this meta-analysis.
15. **Fenland:** The Fenland Study is an ongoing, population-based cohort study (started in 2005) designed to investigate the association between genetic and lifestyle environmental factors and the risk of obesity, insulin sensitivity, hyperglycemia and related metabolic traits in men and women aged 30 to 55 years. Potential volunteers were recruited from General Practice sampling frames in the Fenland, Ely and Cambridge areas of the Cambridgeshire Primary Care Trust in the UK. Exclusion criteria for the study were: prevalent diabetes, pregnant and lactating women, inability to participate including terminal illness, psychotic illness, or inability to walk unaided. All participants had measurements done at the MRC Epidemiology Unit Clinical Research Facilities in Ely, Wisbech and Cambridge. Participants attended after an overnight fast for a detailed clinical examination, and blood samples were collected. The Local Research Ethics Committee granted ethical approval for the study and all participants gave written informed consent^18^. Data from 1,396 participants with available adiponectin measurements and genotype data were included in this analysis.

II. **Replication phase cohorts:**

**Phase 1:**

1. **HABC**

The HABC described in detail above. The current study sample consists of 1661 white participants who attended the second exam in 1998-1999 with available adiponectin measurements and genotype data were included in this analysis.

1. **Nurses’ Health Study (NHS):** The NHS has been described in detail previously.^22^ The NHS study was established in 1976 when 121,700 female registered nurses aged 30–55 years and residing in 11 large U.S. states completed a mailed questionnaire on their medical history and lifestyle. Data from 1,585 participants with available adiponectin measurements and genotype data were included in this analysis.
2. **Health Professionals Follow-Up Study (HPFS):** The HPFS has been described in detail previously.^22^ The HPFS is a prospective cohort study of 51,529 U.S. male health professionals aged 40 to 75 years at study initiation in 1986. Information about health and disease is assessed biennially by a self-administered questionnaire. 683 individuals with available genotypic and adiponectin data were included in the current analysis.
3. **Leiden Longevity Study (LLS)**: The LLS has been described in detail previously.^23^ It is family based study consists of 1671 offspring of 421 nonagenarians sibling pairs of Dutch descent, and their 744 partners. 1,921 individuals with available genotypic and adiponectin data were included in the current analysis.
4. **Genetics, Arthrosis , and Progression) study (GARP).** The GARP study has been described in detail previously.^24^ It aimed at identifying determinants of osteoarthritis and the progression of this disease. The study is based on sibships of white Dutch ancestry with clinical- and radiographically-confirmed osteoarthritis at two or more joint sites of the hand, spine (cervical or lumbar), knee or hip. 149 individuals with available genotypic and adiponectin data were included in the current analysis.
5. **ERF2.** A subset of 320 individuals from this cohort, as described above, is included in this analysis.
6. **Atherosclerosis Risk in Communities (ARIC)**. The ARIC Study is a population-based, longitudinal study. A total of 15,792 participants aged 45-64 were recruited in 1987-89 using probability-based sampling from four US communities: Forsyth County, NC; Jackson, MS; northwest suburbs of Minneapolis, MN; and Washington County, MD^25^. Genotype and adiponectin measurements were available for 303 white individuals selected for the subcohort of a case-cohort study of T2D conducted with ARIC.

**Phase 2:**

1. **KORA F3.** The KORA F3 population-based cohort is representative of the general population of Southern Germany. All study participants underwent a standardized face-to-face interview by certified medical staff and a standardized medical examination including blood draw and anthropometric measurements^26^. 1,466 Individuals who were not part of the GWAS sample were part of the replication sample.
2. **Salzburg Atherosclerosis Prevention Program in subjects at High Individual Risk (SAPHIR).** The SAPHIR is an observational study conducted in the years 1999-2002 involving 1,710 healthy, unrelated subjects.^27^ Study participants were recruited by health screening programs in large companies in and around the city of Salzburg. The aim of this study was to investigate the genetic determinants of metabolic phenotypes. This study has been described in detail previously.^28^ De-novo genotyping has been done the mentioned individuals.
3. **THISEAS.** THISEAS is a case- control study designed to investigate the association between genetic and lifestyle environmental factors and the risk of coronary artery disease in men and women aged >30 years. The control group consists of individuals with no history of cardiovascular disease, while cases are individuals with coronary artery disease. Hematological, biochemical and anthropometric measurements were conducted in all participants. Dietary assessment and physical activity data were collected through face-to-face interview by well-trained scientists. Metabochip was used for DNA analysis. Exclusion criteria for the control group were a history of cardiovascular disease, cancer and/or other inflammatory disease. This analysis is limited to 738 individuals with available adiponectin measurements and metabochip data.

**Cohorts Not of European Descent**

1. **Cardiovascular Health Study (CHS**). The CHS has been described in detail previously^14^. The CHS is a population-based cohort study of risk factors for CHD and stroke in adults ≥65 years conducted across four field centers. The original cohort of predominantly European individuals (n=5,201) was recruited in 1989-1990 from random samples of the Medicare eligibility lists; subsequently, an additional predominantly African-American cohort of 687 individuals was enrolled, resulting in a total sample of 5,888. This analysis includes 724 African-American individuals with genotype data and adiponectin measurements.
2. **Cleveland Family Study (CFS).** The CFS is a family-based, longitudinal study designed to characterize the genetic and non-genetic risk factors for sleep apnea and related traits.^19^ In *total*, 2534 individuals (46% African American) from 352 families were studied on up to 4 occasions over a period of 16 years (1990-2006). The total sample included index probands (n=275) who were recruited from 3 area hospital sleep centers if they had a confirmed diagnosis of sleep apnea and at least 2 first-degree relatives available to be studied. In the first 5 years of the study, neighborhood control probands (n=87) with at least 2 living relatives available for study were selected at random from a list provided by the index family. All available first-degree relatives and spouses of the case and control probands were recruited. Second-degree relatives, including half-sibs, aunts, uncles and grandparents, were also included if they lived near the first degree relatives (cases or controls), or if the family had been found to have two or more relatives with sleep apnea. Data from 356 participants with available adiponectin measurements and genotype data were included in this analysis.
3. **The Multiethnic Study of Atherosclerosis (MESA).** The MESA is a longitudinal cohort study of 4 ethnic groups to include African, Chinese and Hispanic Americans, as well as non-Hispanic Whites. Details about the study design for the MESA have been published.^20^ In brief, between July 2000 and August 2002, 6,814 men and women who were 45 to 84 years old and were free of clinically apparent cardiovascular disease (CVD) were recruited from six United States communities. Individuals with a history of physician-diagnosed heart attack, angina, heart failure, stroke or TIA, or having undergone an invasive procedure for cardiovascular disease (CABG, angioplasty, valve replacement or pacemaker placement) were excluded from participation.  Enrolled participants returned for follow-up clinic visits approximately 2, 4 and 6 years after the baseline clinic visit. Data from 353 participants with available adiponectin measurements and genotype data were included in this analysis.
4. **Jackson Heart Study (JHS).** The JHS is a large, population-based observational study evaluating the etiology of cardiovascular, renal and respiratory diseases.^21^ Data and biologic materials have been collected from a probability community-based sample of African American adults composed of 5301 participants, including 1499 members of 291 families. Participants were enrolled from the three counties that make up the Jackson, Mississippi metropolitan area. Relatives of selected participants were recruited to develop a large, nested family cohort. The JHS participants, aged 21 – 94 years, were recruited and examined at baseline (2000-2004) by certified technicians according to standardized protocols. Clinic visits and interviews occurred approximately every three years. Participants provided extensive medical and social history, had an array of physical and biochemical measurements and diagnostic procedures, and provided genomic DNA. Participants have a high prevalence of diabetes, hypertension, obesity, and related disorders. DNA samples were obtained from 4726 JHS participants. Annual follow-up interviews and cohort surveillance are ongoing. Data from 1,662 participants with available adiponectin measurements and genotype data were included in this analysis.
5. **Cebu Longitudinal Health and Nutrition Survey (CLHNS)**. The CLHNS is an on-going community-based study that began in 1983. The baseline survey randomly recruited 3,327 mother-child pairs from 17 urban and 16 rural areas from the Metropolitan Cebu area, the Philippines. Overnight fasting blood samples for biomarkers and DNA were obtained at the 2005 survey. Data from 1,776 participants with available adiponectin measurements and genotype data were included in this analysis.
6. **Dynamics of Health, Aging and Body Composition (HABC)**: The HABC study is a prospective cohort study investigating the associations between body composition, weight-related health conditions, and incident functional limitation in older adults.  Health ABC enrolled well-functioning, community-dwelling men and women aged 70-79 years between April 1997 and June 1998.  Participants were recruited from a random sample of white and all black Medicare eligible residents in the Pittsburgh, PA, and Memphis, TN, metropolitan areas.  Participants have undergone annual exams and semi-annual phone interviews.  Data from 1,137 participants with available adiponectin measurements and genotype data were included in this analysis.

**Additional Cohorts:**

1. **MRC Ely.** The MRC Ely Study is a population-based cohort randomly selected from people living in Ely and surrounding villages (East Anglia, UK), an ethnically homogenous European ancestry population. The study design, methods and measurements of the three phases have been described in detail elsewhere. The current analyses included 1,700 men and women, aged 35-79 years, from phase 3. Height and weight were measured according to standardized protocols. Ethical permission was granted by the Cambridgeshire Research Ethics Committee, and study participants provided written informed consent.  Data was available in 1592 subjects for HDL-C and 724 for adiponectin.^29^
2. **The London Life Sciences Prospective Population (LOLIPOP).** LOLIPOP is a population based study including Indian Asian and European white men and women recruited from the lists of 58 General Practitioners in West London.  6586 white subjects were used in the analysis of GPR109A R311C and HDL-C, adiponectin was not available.^30; 31^

**References**

1. Timpson, N.J., Tobias, J.H., Richards, J.B., Soranzo, N., Duncan, E.L., Sims, A.M., Whittaker, P., Kumanduri, V., Zhai, G., Glaser, B., et al. (2009). Common variants in the region around Osterix are associated with bone mineral density and growth in childhood. Hum Mol Genet 18, 1510-1517.

2. Shock, N.W., Greulich, R.C., Andres, R.A., Arenberg, D., Costa, P.T., and Lakatta, E.W. (1984). Normal Human Aging: The Baltimore Longitudinal Study of Aging. NIH Publication No 84-2450.

3. Firmann, M., Mayor, V., Vidal, P.M., Bochud, M., Pecoud, A., Hayoz, D., Paccaud, F., Preisig, M., Song, K.S., Yuan, X., et al. (2008). The CoLaus study: a population-based study to investigate the epidemiology and genetic determinants of cardiovascular risk factors and metabolic syndrome. BMC Cardiovasc Disord 8, 6.

4. Aulchenko, Y.S., Heutink, P., Mackay, I., Bertoli-Avella, A.M., Pullen, J., Vaessen, N., Rademaker, T.A., Sandkuijl, L.A., Cardon, L., Oostra, B., et al. (2004). Linkage disequilibrium in young genetically isolated Dutch population. Eur J Hum Genet 12, 527-534.

5. Dawber, T.R., Kannel, W.B., and Lyell, L.P. (1963). An approach to longitudinal studies in a community: the Framingham Study. Ann N Y Acad Sci 107, 539-556.

6. Stirnadel, H., Lin, X., Ling, H., Song, K., Barter, P., Kesaniemi, Y.A., Mahley, R., McPherson, R., Waeber, G., Bersot, T., et al. (2008). Genetic and phenotypic architecture of metabolic syndrome-associated components in dyslipidemic and normolipidemic subjects: the GEMS Study. Atherosclerosis 197, 868-876.

7. Ferrucci, L., Bandinelli, S., Benvenuti, E., Di Iorio, A., Macchi, C., Harris, T.B., and Guralnik, J.M. (2000). Subsystems contributing to the decline in ability to walk: bridging the gap between epidemiology and geriatric practice in the InCHIANTI study. J Am Geriatr Soc 48, 1618-1625.

8. Melzer, D., Perry, J.R., Hernandez, D., Corsi, A.M., Stevens, K., Rafferty, I., Lauretani, F., Murray, A., Gibbs, J.R., Paolisso, G., et al. (2008). A genome-wide association study identifies protein quantitative trait loci (pQTLs). PLoS Genet 4, e1000072.

9. Lowel, H., Doring, A., Schneider, A., Heier, M., Thorand, B., and Meisinger, C. (2005). The MONICA Augsburg surveys--basis for prospective cohort studies. Gesundheitswesen 67 Suppl 1, S13-18.

10. Pattaro, C., Marroni, F., Riegler, A., Mascalzoni, D., Pichler, I., Volpato, C.B., Dal Cero, U., De Grandi, A., Egger, C., Eisendle, A., et al. (2007). The genetic study of three population microisolates in South Tyrol (MICROS): study design and epidemiological perspectives. BMC Med Genet 8, 29.

11. Pattaro, C., Aulchenko, Y.S., Isaacs, A., Vitart, V., Hayward, C., Franklin, C.S., Polasek, O., Kolcic, I., Biloglav, Z., Campbell, S., et al. (2009). Genome-wide linkage analysis of serum creatinine in three isolated European populations. Kidney Int 76, 297-306.

12. Andrew, T., Hart, D.J., Snieder, H., de, L.M., Spector, T.D., and MacGregor, A.J. (2001). Are twins and singletons comparable? A study of disease-related and lifestyle characteristics in adult women. Twin Res 4, 464-477.

13. Richards, J.B., Rivadeneira, F., Inouye, M., Pastinen, T.M., Soranzo, N., Wilson, S.G., Andrew, T., Falchi, M., Gwilliam, R., Ahmadi, K.R., et al. (2008). Bone mineral density, osteoporosis, and osteoporotic fractures: a genome-wide association study. Lancet 371, 1505-1512.

14. Fried, L.P., Borhani, N.O., Enright, P., Furberg, C.D., Gardin, J.M., Kronmal, R.A., Kuller, L.H., Manolio, T.A., Mittelmark, M.B., Newman, A., et al. (1991). The Cardiovascular Health Study: design and rationale. Ann Epidemiol 1, 263-276.

15. Raitakari, O.T., Juonala, M., Ronnemaa, T., Keltikangas-Jarvinen, L., Rasanen, L., Pietikainen, M., Hutri-Kahonen, N., Taittonen, L., Jokinen, E., Marniemi, J., et al. (2008). Cohort profile: the cardiovascular risk in Young Finns Study. Int J Epidemiol 37, 1220-1226.

16. Eriksson, J.G. (2005). Early growth and adult health outcomes--lessons learned from the Helsinki Birth Cohort Study. Matern Child Nutr 1, 149-154.

17. Inouye, M., Silander, K., Hamalainen, E., Salomaa, V., Harald, K., Jousilahti, P., Mannisto, S., Eriksson, J.G., Saarela, J., Ripatti, S., et al. (2010). An immune response network associated with blood lipid levels. PLoS Genet 6.

18. Speliotes, E.K., Willer, C.J., Berndt, S.I., Monda, K.L., Thorleifsson, G., Jackson, A.U., Allen, H.L., Lindgren, C.M., Luan, J., Magi, R., et al. (2010). Association analyses of 249,796 individuals reveal 18 new loci associated with body mass index. Nat Genet 42, 937-948.

19. Buxbaum, S.G., Elston, R.C., Tishler, P.V., and Redline, S. (2002). Genetics of the apnea hypopnea index in Caucasians and African Americans: I. Segregation analysis. Genet Epidemiol 22, 243-253.

20. Bild, D.E., Bluemke, D.A., Burke, G.L., Detrano, R., Diez Roux, A.V., Folsom, A.R., Greenland, P., Jacob, D.R., Jr., Kronmal, R., Liu, K., et al. (2002). Multi-ethnic study of atherosclerosis: objectives and design. Am J Epidemiol 156, 871-881.

21. Taylor, H.A., Jr., Wilson, J.G., Jones, D.W., Sarpong, D.F., Srinivasan, A., Garrison, R.J., Nelson, C., and Wyatt, S.B. (2005). Toward resolution of cardiovascular health disparities in African Americans: design and methods of the Jackson Heart Study. Ethn Dis 15, S6-4-17.

22. Qi, L., Cornelis, M.C., Kraft, P., Jensen, M., van Dam, R.M., Sun, Q., Girman, C.J., Laurie, C.C., Mirel, D.B., Hunter, D.J., et al. (2010). Genetic variants in ABO blood group region, plasma soluble E-selectin levels and risk of type 2 diabetes. Hum Mol Genet 19, 1856-1862.

23. Schoenmaker, M., de Craen, A.J., de Meijer, P.H., Beekman, M., Blauw, G.J., Slagboom, P.E., and Westendorp, R.G. (2006). Evidence of genetic enrichment for exceptional survival using a family approach: the Leiden Longevity Study. Eur J Hum Genet 14, 79-84.

24. Riyazi, N., Meulenbelt, I., Kroon, H.M., Ronday, K.H., Hellio le Graverand, M.P., Rosendaal, F.R., Breedveld, F.C., Slagboom, P.E., and Kloppenburg, M. (2005). Evidence for familial aggregation of hand, hip, and spine but not knee osteoarthritis in siblings with multiple joint involvement: the GARP study. Ann Rheum Dis 64, 438-443.

25. (1989). The Atherosclerosis Risk in Communities (ARIC) Study: design and objectives. The ARIC investigators. Am J Epidemiol 129, 687-702.

26. Keating, B.J., Tischfield, S., Murray, S.S., Bhangale, T., Price, T.S., Glessner, J.T., Galver, L., Barrett, J.C., Grant, S.F., Farlow, D.N., et al. (2008). Concept, design and implementation of a cardiovascular gene-centric 50 k SNP array for large-scale genomic association studies. PLoS One 3, e3583.

27. Heid, I.M., Wagner, S.A., Gohlke, H., Iglseder, B., Mueller, J.C., Cip, P., Ladurner, G., Reiter, R., Stadlmayr, A., Mackevics, V., et al. (2006). Genetic architecture of the APM1 gene and its influence on adiponectin plasma levels and parameters of the metabolic syndrome in 1,727 healthy Caucasians. Diabetes 55, 375-384.

28. Musunuru, K., Lettre, G., Young, T., Farlow, D.N., Pirruccello, J.P., Ejebe, K.G., Keating, B.J., Yang, Q., Chen, M.H., Lapchyk, N., et al. (2010). Candidate gene association resource (CARe): design, methods, and proof of concept. Circ Cardiovasc Genet 3, 267-275.

29. Forouhi, N.G., Luan, J., Hennings, S., and Wareham, N.J. (2007). Incidence of Type 2 diabetes in England and its association with baseline impaired fasting glucose: the Ely study 1990-2000. Diabet Med 24, 200-207.

30. Kooner, J.S., Chambers, J.C., Aguilar-Salinas, C.A., Hinds, D.A., Hyde, C.L., Warnes, G.R., Gomez Perez, F.J., Frazer, K.A., Elliott, P., Scott, J., et al. (2008). Genome-wide scan identifies variation in MLXIPL associated with plasma triglycerides. Nat Genet 40, 149-151.

31. Chambers, J.C., Elliott, P., Zabaneh, D., Zhang, W., Li, Y., Froguel, P., Balding, D., Scott, J., and Kooner, J.S. (2008). Common genetic variation near MC4R is associated with waist circumference and insulin resistance. Nat Genet 40, 716-718.

32. Hivert, M.F., Manning, A.K., McAteer, J.B., Florez, J.C., Dupuis, J., Fox, C.S., O'Donnell, C.J., Cupples, L.A., and Meigs, J.B. (2008). Common variants in the adiponectin gene (ADIPOQ) associated with plasma adiponectin levels, type 2 diabetes, and diabetes-related quantitative traits: the Framingham Offspring Study. Diabetes.

33. Richards, J.B., Valdes, A.M., Burling, K., Perks, U.C., and Spector, T.D. (2007). Serum adiponectin and bone mineral density in women. J Clin Endocrinol Metab 92, 1517-1523.
